# Multiple target drug cocktail design for attacking the core network markers of four cancers using ligand-based and structure-based virtual screening methods

**DOI:** 10.1186/1755-8794-8-S4-S4

**Published:** 2015-12-09

**Authors:** Yung-Hao Wong, Chih-Lung Lin, Ting-Shou Chen, Chien-An Chen, Pei-Shin Jiang, Yi-Hua Lai, Lichieh Julie Chu, Cheng-Wei Li, Jeremy JW Chen, Bor-Sen Chen

**Affiliations:** 1 Laboratory of Control and Systems Biology, Department of Electrical Engineering, National Tsing Hua University, Hsinchu 30013, Taiwan; 2 Biomedical Technology and Device Research Laboratories, Industrial Technology Research Institute, Hsinchu, Taiwan, ROC; 3 Institute of Biomedical Science, National Chung Hsing University, Taiwan 40227, Republic of China; 4 Molecular Medicine Research Center, Chang Gung University, Taoyuan, Taiwan,ROC

**Keywords:** Multiple drug targets, core network marker, ligand-based CADD, structure-based CADD, virtual screening, pharmacophore, systems biology

## Abstract

**Background:**

Computer-aided drug design has a long history of being applied to discover new molecules to treat various cancers, but it has always been focused on single targets. The development of systems biology has let scientists reveal more hidden mechanisms of cancers, but attempts to apply systems biology to cancer therapies remain at preliminary stages. Our lab has successfully developed various systems biology models for several cancers. Based on these achievements, we present the first attempt to combine multiple-target therapy with systems biology.

**Methods:**

In our previous study, we identified 28 significant proteins--i.e., common core network markers--of four types of cancers as house-keeping proteins of these cancers. In this study, we ranked these proteins by summing their carcinogenesis relevance values (CRVs) across the four cancers, and then performed docking and pharmacophore modeling to do virtual screening on the NCI database for anti-cancer drugs. We also performed pathway analysis on these proteins using Panther and MetaCore to reveal more mechanisms of these cancer house-keeping proteins.

**Results:**

We designed several approaches to discover targets for multiple-target cocktail therapies. In the first one, we identified the top 20 drugs for each of the 28 cancer house-keeping proteins, and analyzed the docking pose to further understand the interaction mechanisms of these drugs. After screening for duplicates, we found that 13 of these drugs could target 11 proteins simultaneously. In the second approach, we chose the top 5 proteins with the highest summed CRVs and used them as the drug targets. We built a pharmacophore and applied it to do virtual screening against the Life-Chemical library for anti-cancer drugs. Based on these results, wet-lab bio-scientists could freely investigate combinations of these drugs for multiple-target therapy for cancers, in contrast to the traditional single target therapy.

**Conclusions:**

Combination of systems biology with computer-aided drug design could help us develop novel drug cocktails with multiple targets. We believe this will enhance the efficiency of therapeutic practice and lead to new directions for cancer therapy.

## Background

Cancer is the leading cause of human death worldwide. It is a complex set of diseases, and people have tried to reveal its underlying mechanisms to guide the development of novel therapy strategies. In the last two decades, cancer researchers have generated an abundance of knowledge about cancer, and revealed the etiology of various cancers at DNA, RNA, and protein levels [1]. Weinberg et al. summarized the first and next generation of cancer hallmarks to expand the current understanding of the basic mechanisms of cancer [2,3]. Recently, due to the scale up in high throughput data, availability of integrated OMICS data, and various advanced statistical analysis methods, many novel systems biology approaches have been employed to reveal the deeper underlying systematic mechanisms of various cancers [4-6].

Traditional computer-aided drug design (CADD) focuses on a single target for therapy, such as Src, FAK, and EGFR in the case of cancer [7,8]. Researchers have used virtual screening with *de novo *methods to develop small molecules that in most cases inhibit these targets (although sometimes they are agonists), and accordingly reduce the expression of these proteins to kill cancer cells. CADD has a long history and many successful examples. CADD methods can be divided into structure-based and ligand-based methods [9]. Methods in the former category analyze both the structures of the target protein and the small molecule inhibitors to design drugs: examples include the docking method and molecular dynamics simulations. On the other hand, methods in the latter category use only the structures of the small molecule inhibitors (drugs) to do statistical calculations to determine the relationship between a drug's IC_50_ and its corresponding molecular properties: examples include HypoGen pharmacophore modeling, COMFA (and COMSIA) [10], and many other machine learning and regression methods [11,12].

One of the main differences between Western and Chinese medical philosophy is that the former focuses on single targets, while the latter focuses on multiple targets simultaneously [13]. Systems biology reconstructs the regulatory relationships within genetic, metabolic, and protein-protein interaction networks. These biological networks are highly complex, so robustness and sensitivity are their key system-level features [14,15]. The intertwined nature of these networks shows us that inhibiting a single protein directly is not the only way to depress its expression. Systems biology will helps us to identify several protein targets to be inhibited simultaneously: due to their network behaviour, this multi-target approach will produce the same or better effect, than focusing on a single target protein. Also, recent research has demonstrated that to inhibit protein-protein interactions (PPI) is another novel anti-cancer strategy [16]. Inspired by the above ideas, based on the result of our previous systems biology studies [17], we have developed a novel multi-target cocktail therapy to focus on common core network markers of four different cancers. Our strategy is different from Dr. Ho's famous cocktail therapy for AIDS [18], which is not targeted therapy. Our method is to apply traditional CADD methods simultaneously to multiple drug targets. We regard this as a great advance in novel anti-cancer strategies.

Theoretical biological background: Recently, PPI-based analysis seems to have become a novel strategy or cancer target drug therapy and the development of precision medicine [16,19]. Unlike traditional target drug design, which focuses on the inhibition or activation of a single target protein, usually a receptor or enzyme, PPI-based drug design involves inhibition of PPIs interface that mediate many important biological processes by small molecule; it is a novel and creative approach to drug discovery, especially for anti-cancer. Many clinical and elementary biological researches have concluded that the identification of PPI nodes and hubs that are significant for cell transformation functions in cancer. These PPIs related to cancers have become important targets for cancer therapy. Progress on technologies in the identification of PPI modulators and the clinical validation of the PPI pairs has made anti-cancer therapeutics by interfering with PPIs a reality [16,19].

So, to identify PPI interface and PPI targets are regarded as the future topics for next generation anticancer strategies. Nevertheless, the cancer PPI networks are always highly complex and differ between cancer subtypes. Hence, we put concentration on common PPI network markers and their PPIs with a critical carcinogenesis relevance value (CRV), which is an estimate of the PPI evolution during the carcinogenesis process, to focus on the conservation of house-keeping proteins and their PPI interface characteristics as important targets shared by different cancers. This allows us to not only find out the crucial common pathways of different cancers in carcinogenesis, but also discover novel PPI targets for cancer therapy. Specific network markers can be regarded to represent specific PPI targets for each cancer. To target PPIs in both common core network markers and specific network markers simultaneously may provide new directions for anticancer therapy strategies.

Theoretical, mathematical, and statistical method: The traditional methods to find hubs or driven proteins in the gene regulatory or PPI networks of cancers is different from our method. In our previous study [17], we compared between the networks of each cancer and its corresponding normal PPI, which were obtained by the AIC (akaike information criterion) order detection and Student t-test methods from microarray expression data of patients and normal people, respectively, to get the PPI differential network in order to reveal PPI alternations during the tumorigenesis process. And then, we developed a carcinogenesis relevance value (CRV) for each protein in the PPI differential network based on the total alternations of PPI interaction abilities with other proteins to approve the critical PPI changes during tumorigenesis process. In the end, we obtained the core and specific network markers by using the intersection and difference of these 4 cancers with top CRVs. These novel core and specific network markers could provide possible PPI targets for small-molecule drugs to interfere and then destroy tumors. Calculations and estimations were using real microarray expression data. The maximum likelihood parameter estimation method and AIC model order selection method are well-known and widely used system identification methods from experimental data. During these estimation and learning processes, the PPI interaction mathematical model can derive the most probable PPI network for cancer and normal patients from large amount of microarray data and big databases to interpret the hidden biological mechanisms. The above paragraphs are adapted from our previous study [17] to make this paper be a complete paper.

In our previous study, we analyzed various cancers--specifically, bladder, colorectal, liver, and lung cancers--through regression modeling, microarray data, maximum likelihood parameter estimations, and big database mining. Based on known PPI information and gene expression data from normal and patient samples, we built a cancer PPI network [17]. Here, we focus on not only the PPI networks of single cancer types but also on common core network markers of four different cancers through the intersection of their respective PPI networks. Cancer is a complex disease, and so we tried to reveal house-keeping proteins significant in different cancers, i.e. the common core network markers shared by the different cancers. As the first trial to develop multiple-target therapy, cancer house-keeping proteins (common core network markers) may be a good choice. There are two main parts of this research. One is to find the core network markers of four cancers by systems biology approach, and the other is to attach these network markers by ligand-based and structure-based CADD (computer-aided drug design) methods. The first part has been published on Journal of Theoretical Biology [17]. The whole work of this research is the Part-II, i.e., using the CADD methods to attach the network biomarkers. To make this paper complete, so we described how to get these network biomarkers in our previous research [17].

## Materials and methods

Identification of the core network markers of four cancers (28 proteins) - A brief review of our previous methods

In our previous study [17], we used the systems biology approach to study network markers of various cancers. Firstly, microarray data, PPI databases and PPI interaction models were employed to construct the PPI networks of normal and cancer cells by the maximum likelihood parameter estimation method (see Additional file 1). The AIC system order detection method (Additional file 2) was employed to prune false-positive PPIs to obtain real PPI networks of normal and cancer cells: in other words, we used the reverse engineering method to construct the PPI networks of normal and cancer cells. Then, the differential PPI network--obtained by contrasting the cancer PPI network and normal PPI network--was used to investigate PPI variations of each protein in the differential PPI network due to carcinogenesis. Finally, the carcinogenesis value (CRV) based on PPI variations was proposed to evaluate the significance of each protein for carcinogenesis in the differential PPI network. Proteins with a significant CRV (p-value<0.01) were considered to be significant for the progress of the cancer. The complete mathematical model is described as follows.

After organizing the cancer microarray data and PPI data, we used a PPI model, the maximum likelihood parameter estimation method and a model order detection method together to prune each candidate PPIN by the corresponding microarray data to approach the actual PPIN of each cancer. Here, the PPIs of a target protein *i *in the candidate PPIN can be depicted by the following protein association model:

(1) $$x_{i}\left[n\right]=\overset{M_{i}}{\underset{j=1}{\sum }}\alpha_{ij}x_{j}\left[n\right]+\omega_{i}\left[n\right]$$

where $x_{i}\left[n\right]$ represents the expression level of the target protein *i *for the sample *n*;$x_{j}\left[n\right]$ represents the expression level of the *j*-th protein interacting with the target protein *i *for the sample *n*; $\alpha_{ij}$denotes the association ability between the target protein *i *and its *j*-th interactive protein; $M_{i}$ represents the number of proteins interacting with the target protein *i*; and $\omega_{i}\left[n\right]$ represents the stochastic noise due to other factors or model uncertainty. The biological meaning of equation (1) is that the expression level of the target protein *i *is associated with the expression levels of the proteins interacting with it. Consequently, a protein association (interaction) model for each protein in the protein pool can be built.

After constructing equation (1) for the PPI model of each protein in the candidate PPIN, we used the maximum likelihood estimation method [20] to identify the association parameters in (1) by microarray data as follows (see Additional file 1):

(2) $$x_{i}\left(n\right)=\overset{M_{i}}{\underset{j=1}{\sum }}{\hat{\alpha }}_{ij}x_{j}\left(n\right)+w_{i}\left(n\right)$$

where ${\hat{\alpha }}_{ij}$ is identified by the maximum likelihood estimation method.

Once the association parameters for all proteins in the candidate PPI network were identified for each protein, the true protein associations were determined by pruning the false positive PPIs. Akaike Information Criterion (AIC) [20] and a Student's t-test [21] were employed to achieve model order selection for the pruning of false positive protein associations in ${\hat{\alpha }}_{ij}$ (see Additional file 2).

After the AIC order detection and use of the Student's t-test to determine *p*-values of ${\hat{\alpha }}_{ij}$, the false positive PPIs ${\hat{\alpha }}_{ij}$ in (2) were pruned away and only significant PPIs were refined as follows:

(3) $$x_{i}\left(n\right)=\overset{{M_{i}}^{\prime }}{\underset{j=1}{\sum }}{\hat{\alpha }}_{ij}x_{j}\left(n\right)+{w_{i}}^{\text{'}}\left(n\right),\;i=1,2......M$$

where ${M_{i}}^{\prime }\leq M_{i}$ denotes the number of true PPIs, with the target protein *i*, i.e., a number of $M_{i}-{M_{i}}^{\prime }$ (or false positives) are pruned in the PPIs of target protein *i*. One protein by one protein (i.e., i=1,2,...,M for all proteins in refined PPIN in (3)) results in refined PPIN

(4) X(n)=AX(n)+w(n)

where $X\left(n\right)=\left[\begin{array}{c} x_{1}\left(n\right)\\ x_{2}\left(n\right)\\ \;\vdots \\ x_{M}\left(n\right) \end{array}\right]$, $A=\left[\begin{array}{ccc} {\hat{\alpha }}_{11} & \ldots & {\hat{\alpha }}_{1M}\\ \vdots & \ddots & \vdots \\ {\hat{\alpha }}_{M1} & \cdots \; & {\hat{\alpha }}_{MM} \end{array}\right]$, $w\left(n\right)=\left[\begin{array}{c} {w_{1}}^{\text{'}}\left(n\right)\\ {w_{2}}^{\text{'}}\left(n\right)\\ \;\vdots \\ {w_{M}}^{\text{'}}\left(n\right) \end{array}\right]$

where the interaction matrix *A *denotes the PPIs.

If there is no PPI between protein *i *and protein *j *or it is pruned by AIC order detection due to the false positive PPIs in the refined PPIN, then ${\hat{\alpha }}_{ij}\text{ = 0}$. In general,${\hat{\alpha }}_{ij}\text{ = }{\hat{\alpha }}_{ji}$, but if this is not the case, the larger one was chosen as ${\hat{\alpha }}_{ij}\text{ = }{\hat{\alpha }}_{ji}$to avoid the situation ${\hat{\alpha }}_{ij}\neq {\hat{\alpha }}_{ji}$. The above PPIN construction method was employed to construct the refined PPINs for cancer and non-cancer cells of bladder, colorectal, liver, and lung cancer, respectively.

The interaction matrices A of refined PPINs in (4) for cancer and non-cancer cells of the four cancers were constructed, respectively, as follows:

$A_{C}^{k}=\left[\begin{array}{ccc} {\hat{\alpha }}_{11,C}^{k} & \ldots & {\hat{\alpha }}_{1M,C}^{k}\\ \vdots & \ddots & \vdots \\ {\hat{\alpha }}_{M1,C}^{k} & \cdots \; & {\hat{\alpha }}_{MM,C}^{k} \end{array}\right]$,$A_{N}^{k}=\left[\begin{array}{ccc} {\hat{\alpha }}_{11,N}^{k} & \ldots & {\hat{\alpha }}_{1M,N}^{k}\\ \vdots & \ddots & \vdots \\ {\hat{\alpha }}_{M1,N}^{k} & \cdots \; & {\hat{\alpha }}_{MM,N}^{k} \end{array}\right]$ (5)

where k = bladder cancer, colorectal cancer, liver cancer, and lung cancer; $A_{C}^{k}$ and$A_{N}^{k}$ denote the interaction matrices of refined PPIN of the *k-*th cancer and non-cancer, respectively; *M *is the number of proteins in the refined PPIN. Therefore, the protein association model for CPPIN and NPPIN in the *k-*th cancer and non-cancer can be represented by the following equations according to (4) and (5):

(6) $$\begin{array}{c} x_{C}^{k}\left(n\right)=A_{C}^{k}x_{C}\left(n\right)+w_{C}^{k}\left(n\right)\\ x_{N}^{k}\left(n\right)=A_{N}^{k}x_{N}\left(n\right)+w_{N}^{k}\left(n\right) \end{array}$$

where *k *= bladder cancer, colorectal cancer, liver cancer, and lung cancer;

$x_{C}^{k}\left(n\right)={\left[x_{1C}^{k}\;x_{2C}^{k}\;\cdots \;x_{MC}^{k}\right]}^{T}$and$x_{N}^{k}\left(n\right)={\left[x_{1N}^{k}\;x_{2N}^{k}\;\cdots \;x_{MN}^{k}\right]}^{T}$ denote the vectors of expression levels;$w_{C}^{K}\left(n\right)$ and $w_{N}^{K}\left(n\right)$indicate the noise vectors of PPINs in the *k-*th cancer and non-cancer cells, respectively.

The different matrix $A_{C}^{k}\text{ - }A_{N}^{k}$ of differential PPI network between CPPIN and NPPIN in the *k-*th cancer is defined as:

(7) $$D^{k}=\left[\begin{array}{ccc} d_{\text{11}}^{k} & \ldots & d_{1M}^{k}\\ \vdots & \ddots & \vdots \\ d_{M1}^{k} & \cdots \; & d_{MM}^{k} \end{array}\right]=\left[\begin{array}{ccc} {{\hat{\alpha }}^{k}}_{11,C}-{{\hat{\alpha }}^{k}}_{11,N} & \ldots & {{\hat{\alpha }}^{k}}_{1M,C}-{{\hat{\alpha }}^{k}}_{1M,N}\\ \vdots & \ddots & \vdots \\ {{\hat{\alpha }}^{k}}_{M1,C}-{{\hat{\alpha }}^{k}}_{M1,N} & \cdots \; & {\hat{\alpha }}_{MM,C}^{k}-{\hat{\alpha }}_{MM,N}^{k} \end{array}\right]$$

where *k *= bladder cancer, colorectal cancer, liver cancer, and lung cancer; $d_{ij}^{k}$ denotes the PPI variation between the *i*-th protein and the j-th protein of differential PPI network by comparing CPPIN with NPPIN in the *k*-th type of cancer; the matrix $D^{k}$ indicates the difference in network structure between CPPIN and NPPIN in the *k-*th type of cancer. In order to investigate carcinogenesis from the difference matrix $D^{k}$ between CPPIN and NPPIN of the *k-*th cancer in (7), a score named the carcinogenesis relevance value (CRV) was presented to quantify the correlation of PPI variations of each protein in $D^{k}$ with the significance of carcinogenesis as follows [22]:

(8) $$CRV^{k}=\left[\begin{array}{c} CRV_{1}^{k}\\ \vdots \\ CRV_{i}^{k}\\ \vdots \\ CRV_{M}^{k} \end{array}\right]$$

where $CRV_{i}^{k}=\overset{M}{\underset{j=1}{\sum }}\left|d_{ij}^{k}\right|$, and *k *= bladder cancer, colorectal cancer, liver cancer, and lung cancer.

The $CRV_{i}^{k}$ in (8) quantifies the differential extent of PPI variations of the  i-th protein (the absolute sum of the *i-*th row of $D^{k}$ in (7)) and $CRV_{}^{k}$can differentiate CPPIN from NPPIN in the *k-*th cancer. In other words, the $CRV_{i}^{k}$ in (8) could calculate the total PPI variations of the *i-*th protein by comparing the network structure differences between the cancer and non-cancer networks, which can be used to check which proteins are involved with the *k-*th cancer.

In order to investigate which proteins are more likely involved in the *k-*th cancer, we needed to calculate the corresponding empirical *p*-value to determine the statistical significance of $CRV_{i}^{k}$. To determine the observed *p*-value of each $CRV_{i}^{k}$, we repeatedly permuted the network structure of the candidate PPIN of the *k-*th type of cancer as a random network of the *k-*th cancer. Each protein in the random network of the *k-*th type of cancer will have its own CRV to generate a distribution of $CRV_{i}^{k}$ for *k *= bladder, colorectal, liver, and lung cancer. Although there was random disarrangement of the network structure, the linkages of each protein were maintained, i.e., the proteins with which a particular protein interacted were permuted without changing the total number of protein interactions. This procedure was repeated 100,000 times and the corresponding *p*-value was calculated as the fraction of random network structure in which $CRV_{i}^{k}$is at least as large as the CRV of the real network structure. According to the distributions of $CRV_{i}^{k}$ of random networks, the $CRV_{i}^{k}$ in (8) with a *p*-value of less than or equal to 0.01 was regarded as a significant CRV and the corresponding protein was determined to be a significant protein in the carcinogenesis of the *k*-th cancer: a protein with a *p*-value > 0.01 was removed from the list of significant proteins in carcinogenesis (in other words, if the *p*-value of $CRV_{i}^{k}$ > 0.01, then the *i*-th protein was removed from $CRV_{}^{k}$ in (8) and the remainders in $CRV_{}^{k}$ with *p*-values of CRVs less than 0.01 were significant proteins of the *k-*th cancer). Based on the *p*-value of the CRVs for all proteins (i=1,2,...,M) and the four types of cancer (*k *= bladder, colorectal, liver, and lung cancer), we generated four lists of significant proteins (Additional file 3: Table S1) for the cancers according to the CRV and the statistical assessment of each significant protein in $CRV_{}^{k}$ in (8). As shown in Table S1, we found 107 significant proteins in bladder cancer, 110 significant proteins in liver cancer, 60 significant proteins in colorectal cancer, and 86 proteins in lung cancer. These proteins have significant PPI changes between the CPPIN and NPPIN in the carcinogenic process for their corresponding cancer and we suspect that they may play important roles in carcinogenesis, warranting further investigation.

The intersection of these significant proteins in the four cancers and their PPIs is known as the core network markers, while the differences of these significant proteins are the unique significant proteins of each cancer and their PPIs in each of the cancers are known as the specific network markers for each cancer. We found that there were 28 significant proteins that could be classified as a core network marker and 26, 4, 24, and 13 significant proteins that were specific network markers of bladder, colorectal, liver, and lung cancer, respectively. The core network and specific network markers for the cancers are described in our previous paper [17]. This insight into the carcinogenic mechanisms of common core and specific network markers in different cancers will be discussed in detail in the following section.

**Figure 1:**
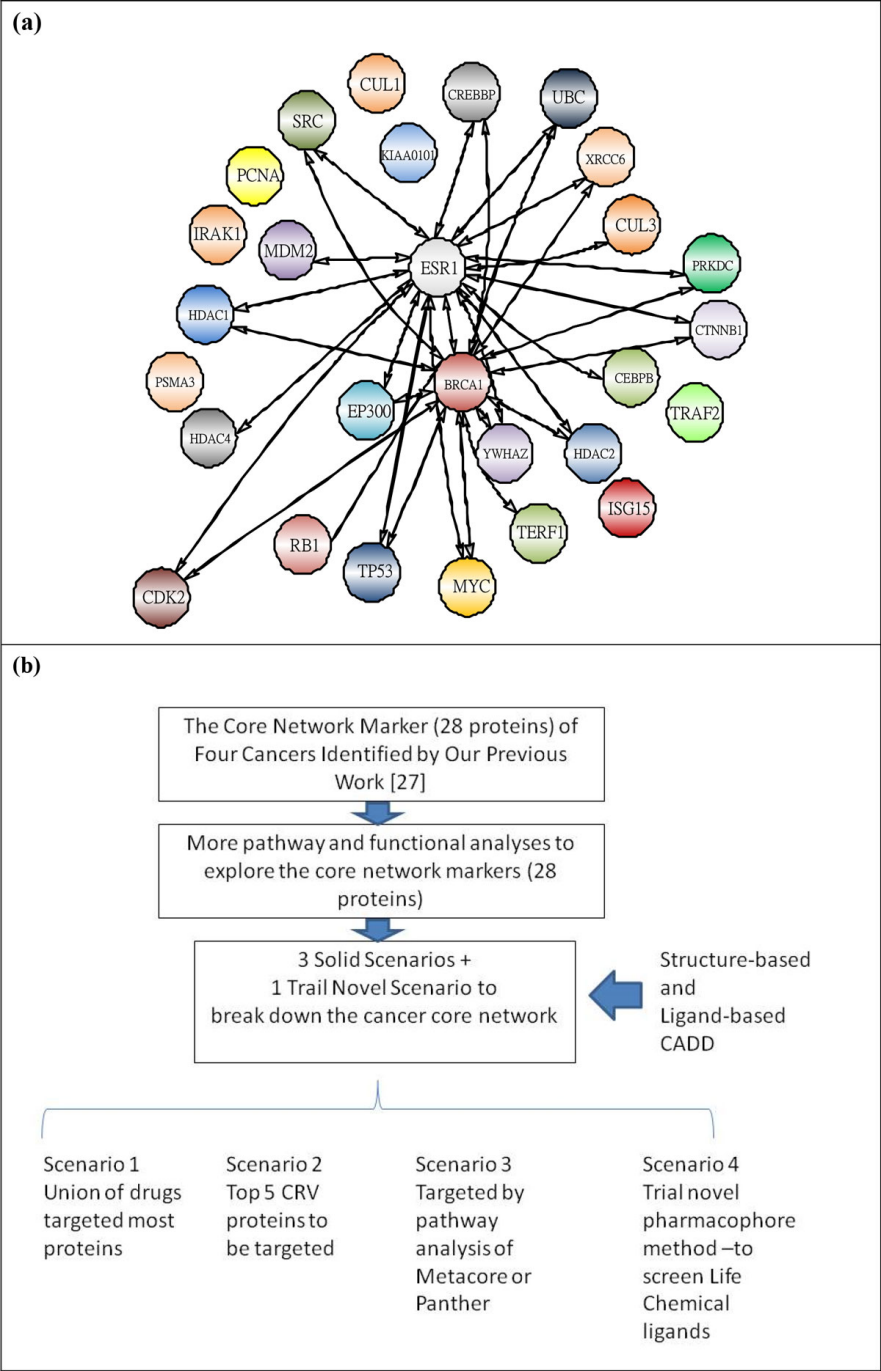
**Core network marker and system flow chart**. (1a) This is the PPI network of the 28 proteins of the core network marker of four cancers. We see both ESR1 and BRCA1 are most highly connected with other proteins. (1b) We use the CADD method to target the 28 core proteins obtained by previous systems biology method. We use both structure-based and ligand-based CADD with pathway and functional analyses to develop 4 scenarios to break down the core network.

The 28 significant proteins in Figure 1(a) (see also Table 1) are significant proteins shared by the four cancers, and these proteins and their PPIs form the common core network markers of these four cancers. The significant proteins outside of these 28 are specific network markers, distinct for each cancer. Finally, based on these common core network markers and specific network markers, we investigated the mechanisms behind the carcinogenesis process with the help of databases (for example, GO database [23], DAVID database [24,25], and KEGG pathway database [26,27]) to find multiple network targets for cancer therapy. Unlike conventional theoretical methods that generate a single mathematical model for a cancer network for detailed theoretical analysis, ours is a systems biology approach to cancer network markers based on real microarray data through reverse engineering, theoretical statistical methods, and data mining in combination with big databases. These features made our method novel and helped produce the significant findings of our paper. The above paragraphs are adapted from our previous study [17] to make this paper to be a complete paper.

**Table 1:** Structure information of 28 core proteins.

No	Protein	PDB id	Resolution[Å]	Chains	Length	Lignd	Full Name
1	BRCA1	4IFI	2.20	A	214	BAAT peptide	Breast cancer type 1 susceptibility protein
2	CDK2	3QQK	1.86	A	306	X02	Cyclin-dependent kinase 2
3	CEBPB	*					CCAAT/enhancer-binding protein beta
4	CREBBP	4A9K	1.81	A, B	119	TYL	CREB-binding protein
5	CTNNB1	3TX7	2.76	A	527	P6L	Catenin (cadherin-associated protein), beta 1
6	CUL1	4F52	3.00	A, C	282	n.a.	Cullin 1
7	CUL3	4EOZ	2.40	B, D	346	n.a.	Cullin-3
8	EP300	4BHW	2.80	A, B	578	01K	E1A binding protein p300
9	ESR1	1UOM	2.28	A	254	PTI	Estrogen receptor 1
10	HDAC1	4BKX	3.00	B	482	n.a.	Histone deacetylase 1
11	HDAC2	4LY1	1.57	A, B, C	369	20Y	Histone deacetylase 2
12	HDAC4	2VQM	1.80	A	413	HA3	Histone deacetylase 4
13	IRAK1	*					Interleukin-1 receptor-associated kinase 1
14	ISG15	3SDL	2.29	C, D	164	n.a.	ISG15 ubiquitin-like modifier
15	KIAA0101	*					KIAA0101
16	MDM2	4MDN	1.90	A	94	Y30	Mouse double minute 2 homolog
17	MYC	1NKP	1.80	A, D	88	n.a.	V-myc myelocytomatosis viral oncogene homolog
18	PCNA	3WGW	2.80	A, B	261	T2B	Proliferating cell nuclear antigen
19	PRKDC	3KGV	6.60	A-F, O, P-T, X-Y	4128	n.a.	Protein kinase, DNA-activated, catalytic polypeptide
20	PSMA3	*					Proteasome subunit alpha type-3
21	RB1	3POM	2.50	A, B	352	n.a.	Retinoblastoma 1
22	SRC	2SRC	1.50	A	452	ANP	Proto-oncogene tyrosine-protein kinase Src
23	TERF1	3BQO	2.00	A	211	TIN2 peptide	Telomeric repeat-binding factor 1
24	TP53	1TSR	2.20	A, B, C	219	n.a.	Tumor protein p53
25	TRAF2	1D0A	2.00	A-F	168	OX40L peptide	TNF receptor-associated factor 2
26	UBC	4FJV	2.05	B, D	86	n.a.	Ubiquitin C
27	XRCC6	1JEQ	2.70	A	609	n.a.	X-ray repair cross-complementing 6
28	YWHAZ	4HKC	2.20	A	250	alpha-4 peptide	14-3-3 protein zeta/delta

*3D structures prepared by homology model, I-Tasser.

### Several scenarios to break down the cancer core network

In this study, we used several different approaches to break down the cancer core network. This is the key feature of this research, which confers it novelty. In contrast to traditional single target therapy, we used multiple-target cocktail therapy to attack common core network markers, i.e., to inhibit several key proteins simultaneously instead of inhibiting only one single target. We assume that precisely targeting the core network would reduce the activity of the cancer. Based on this assumption, we believe that attacking core network markers simultaneously will be more efficient than attacking only one target. The first important problem is how to choose the group of key proteins to target. The first two novel approaches below to break down the cancer core network are based on the systems biology approach employed in our previous study [17]. We developed the third approach by performing pathway analysis using Panther [28] and MetaCore (GeneGo Inc.) [29]. In addition, we used a 'confusion pharmacophore model' to develop the fourth novel trial approach.

**In the first approach**, we chose the compounds with highest docking scores for each of the 28 significant proteins. Then we removed the redundant compounds, and analyzed which compounds can target more than two proteins simultaneously. The basic strategy is to minimize the number of compounds necessary to target in order to break down the core network. Since there are 28 proteins in this core network, even choosing only one ligand to inhibit each of these 28 proteins would necessitate 28 ligands, which may be unsuitable for wet-lab validation.

**In the second approach**, we used the summation of the CRVs (Table 2 and Table 3) as the criterion to choose the key proteins to inhibit. In our previous study, we showed that CRV quantifies the extent of a protein's association with other proteins, and thus it is optimal to target proteins with the highest summation of CRVs. This strategy depends on the cancer being targeted; in the present study, we focused on the common core network markers of the four cancers, so we chose the highest summation of CRVs across the four cancers.

**Table 2:** (a): CRVs of the 28 core proteins of each of the 4 cancers [17].

Protein Name Nam	CRV of Bladder Cancer	CRV of Liver Cancer	CRV of Colorectal Cancer	CRV of Lung Cancer	CRV SUM of 4 Cancers	Protein Name	CRV SUM (Sorted)
BRCA1	11.5863	6.8236	10.8636	12.5188	41.7923	UBC	496.3222

CDK2	16.7366	14.069	14.4739	9.942	55.2215	TP53	79.4445

CEBPB	5.2303	4.433	5.4262	7.2717	22.3612	KIAA0101	71.9887

CREBBP	12.0995	9.5856	11.5906	11.1892	44.4649	HDAC1	70.5646

CTNNB1	7.6796	9.5993	9.304	4.8015	31.3844	CDK2	55.2215

CUL1	13.2669	11.2802	5.5283	8.5044	38.5798	CUL3	53.8054

CUL3	13.0117	12.9519	16.3169	11.5249	53.8054	MYC	53.3347

EP300	12.1078	13.2218	8.3187	7.1278	40.7761	PCNA	52.9045

ESR1	5.6189	10.3758	6.3873	8.6696	31.0516	HDAC2	47.6069

HDAC1	19.2879	19.5736	11.7823	19.9208	70.5646	CREBBP	44.4649

HDAC2	11.0938	9.7752	18.9463	7.7916	47.6069	BRCA1	41.7923

HDAC4	5.8659	5.8397	9.7845	6.2225	27.7126	EP300	40.7761

IRAK1	4.1157	6.646	7.0177	5.1777	22.9571	CUL1	38.5798

ISG15	4.4856	6.0239	5.8124	4.943	21.2649	YWHAZ	35.9252

KIAA0101	15.8188	16.7663	19.1403	20.2633	71.9887	CTNNB1	31.3844

MDM2	4.5647	4.753	11.5648	6.2816	27.1641	ESR1	31.0516

MYC	13.0423	10.7821	20.0595	9.4508	53.3347	SRC	28.3907

PCNA	13.3217	15.1438	9.6282	14.8108	52.9045	TRAF2	27.7637

PRKDC	4.0781	5.9369	8.5589	5.6736	24.2475	HDAC4	27.7126

PSMA3	5.8022	7.7978	8.0875	5.2831	26.9706	MDM2	27.1641

RB1	6.8922	5.5531	5.9763	8.3205	26.7421	PSMA3	26.9706

SRC	8.8026	4.0767	9.1407	6.3707	28.3907	RB1	26.7421

TERF1	5.4642	4.9046	6.2595	8.0708	24.6991	XRCC6	25.7358

TP53	19.5883	18.7422	25.9329	15.1811	79.4445	TERF1	24.6991

TRAF2	9.2003	4.7703	7.365	6.4281	27.7637	PRKDC	24.2475

UBC	158.5321	137.284	80.3851	120.121	496.3222	IRAK1	22.9571

XRCC6	5.2871	9.5585	6.0231	4.8671	25.7358	CEBPB	22.3612

YWHAZ	8.7995	12.6421	7.9038	6.5798	35.9252	ISG15	21.2649

**Table 3:** (b) Top 5 ligands for the top 5 CRV proteins.

UBC(4FJV)		TP53(1TSR)		KIAA0101		HDAC1(4BKX)		CDK2(3QQK)	
**NCI** **Drug**	**LibDock Score**	**NCI** **Drug**	**LibDock Score**	**NCI** **Drug**	**LibDock Score**	**NCI** **Drug**	**LibDock Score**	**NCI** **Drug**	**LibDock Score**

719481	122.887	673172	126.112	655102	165.722	627865	134.685	680359	108.336
633409	114.187	695409	125.821	669588	164.036	625439	134.097	678636	101.875
672968	111.867	682236	117.725	407811	158.565	647638	125.505	669299	101.27
734999	111.578	695405	115.63	704564	148.676	2426	124.722	679065	101.006
688121	111.501	667504	114.326	698687	147.054	707841	117.076	376791	100.92

**In the third approach**, we employed many new and valuable pathway analyses. Wet-lab cancer researchers can choose the pathway they want to focus on as the therapy target. There are many different combinations dependent on different conditions of cancers, so we do not list all the possibilities. Here, we demonstrate a single example, and others can develop their own scenarios following this example with the help of our docking results. (Additional file 4: Table S2)

**The fourth novel approach **is to develop confusion and individual pharmacophore models of core network markers (28 proteins) to do virtual screening using the compounds stored in the Life Chemical database.

We highlight the different approaches in this section, because they form the crux of this study. Please see Figure (1b). In the following sections, we describe the related methods necessary for each of the approaches.

### More pathway and functional analyses

A. Panther

In our previous studies, we performed pathway analysis using the DAVID database. Here, we expand on the previous analysis by performing more pathway and functional analyses in order to develop a more efficient strategy for multiple target therapy. This approach will allow us to accumulate more information on possible therapy strategies. The 28 proteins comprising the cancer core network markers were analyzed for their molecular functions, molecular processes, and subcellular localizations by the PANTHER (Protein ANalysis THrough Evolutionary Relationships) classification system [28]. PANTHER was designed to classify proteins (and their genes) in order to facilitate high-throughput analysis. It has a friendly user interface, you only need to input the gene list and set up the parameters, you will get the results you want. Please see Additional file 5: Figure S5.1 and the PANTHER website [28]

B. MetaCore

MetaCore includes a manually annotated database of gene interactions and metabolic reactions obtained from the scientific literature, including the most newly updated ones. The enrichment analysis of the biological process was based on the hypergeometric distribution algorithm and relevant pathway maps. The mathematical foundation of MetaCore is shown in supplementary materials.

More pathway and functional analyses are fundamental to learning more about the hidden mechanisms of cancer networks. So, to interpret the results for the third approach in a meaningful way, this topic must be explained and described beforehand. The novelty of this paper is in its combination of systems biology with computer-aided drug design. Our research opens many new directions for multiple-target drug design; we describe only a portion of our results in this paper, for illustrative purposes. Our results clearly show that our approaches show great promise for future research to target special pathways through the design of drugs having multiple targets.

Metacore is also user-friendly software. The various manuals and training materials can be download from public website [30,31], and due to the copyright concern, we do not copy too many material here. Please follow the instructions in these manuals, and you can very well analysis. The mathematical foundation of Metacore is shown in Additional file 6. Of course, the more deeply you understand the underlying statistics meaning, you can do better analysis.

### Protein and ligand structures

The following section illustrates the computer-aided drug design (CADD) strategy applied to target the 28 common core network proteins. The first through third approaches need both the 3D structures of proteins and ligands, while the fourth pharmacophore approach only needs the structures of the ligands. The first thing we need is thus the 3D structures of the proteins. At this stage, 24 of them are available in the well-known PDB database, and we can download them directly, while there are four proteins (CEBPB, IRAK1, KIAA0101 and PSMA3) whose 3D structures have not been solved at this stage (Table 1). We used the NCI (46872 ligands) and Life Chemical (31742 ligands) drug libraries with the filter "anti-cancer". The Developmental Therapeutics Program NCI/NIH offers the NCI-60 cell line screening. The users can download the 3D ligands (drugs) structures from the website, and after the virtual screening, you can request them send you these drugs for free. Life Chemical is a commercial company, you have to pay to get these drugs [32]. All drug structures were prepared and minimized by Discovery Studio 3.5 (DS3.5). People seldom do the virtual screening on multiple targets since it is computationally intense. We performed this work on an IBM server with 160 cores and 1 TB of memory.

### Homology model and binding site prediction

Proteins do not always have 3D structures available in the PDB database. Since only amino acid sequence data without corresponding structures were available for four of the proteins targeted in our study (CEBPB, IRAK1, KIAA0101 and PSMA3) we used homology modeling to predict the structures for these proteins. Among many famous software and webservers, the I-Tasser webserver developed by Zhang et al. [33] is the most well-known homology modeling webserver. We used this server to perform homology modeling of our proteins of interest that lacked available 3D structures. For some well-studied protein targets, structures with embedded ligands are available, so the exact binding site for the docking experiments is known. However, binding site information was unavailable for most proteins, so we used the COACH webserver [34], also developed by Zhang et al., to predict the binding sites. The detailed binding site information is shown in Additional File 7. I-TASSER and COACH are both user friendly webserver. You only need to input the sequence of protein residue into the I-TASSER, and set up the parameters, it will give you the predicted 3D protein structures. Users input the 3D protein structures into the COACH and set up the parameters, and then it will give you the predicted binding sites (Additional file 5: Figure S5.2, S5.3).

### Docking

After prepared the 3D structures of proteins and ligands, and find out the binding site, we can perform docking for virtual screening. We used DS 3.5 to perform docking simulations, and then ranked the docking results based on LibDock score. We chose the top 20 compounds for further analysis, such as the construction of pharmacophore models. The DS 3.5 is also user friendly software, users can download the manual from website, please see the example for parameter setting of docking. Please see Additional file 5: Figure S5.4

### Pharmacophore Model

A. HypoGen method and virtual screening

There are three stages in the generation of the HypoGen model: constructive phase, subtractive phase and optimization phase. (1) Constructive phase: Active ligands within a given range of the maximum activity are chosen initially. The two most active ligands are used to enumerate the pharmacophores. (2) Subtractive phase: After the constructive phase, a database with a large number of pharmacophore structures is generated. The purpose of the subtractive phase is to identify the inactive pharmacophores and eliminate them from the database. (3) Optimization phase: The simulated annealing method is used to modify the scoring function of each hypothesis to be tested. After optimization, the HypoGen method generates the top 10 hypotheses. This study is our first attempt to combine systems biology with CADD. Pharmacophore modeling is one of the most powerful approaches in CADD. Here, we developed a novel prototype pharmacophore model, which is different from the traditional one. As mentioned above, this is a novel idea, and the pharmacophore model is just a prototype: it will require further modification in future studies. In traditional pharmacophore modeling, the IC_50 _value for each drug under investigation is necessary, but our model does not require them. Instead, we linearly transformed LibDock scores to generate putative IC_50 _values, and used these values to build the pharmacophore. We combined the top three ligands for each of the 28 proteins to make a ligand pool, and then used this ligand pool to construct a common pharmacophore; this is qualitatively different from building 28 individual pharmacophores. We build the Hypogen model by the DS 3.5, and it is also user friendly. However, to build a correct Hypogen model is a time consuming work, you need to try the different combination of compounds. For a skill expert with normal computational resource, it often needs at least one month to build the correct model. We show the user interface for detailed parameter setting for your reference. Please refer to Additional file 5 : Figure S5.5.

B. PharmaGist

PharmaGist is another method to construct ligand-based pharmacophores. Because it does not require IC_50 _values of ligands [35], it is easier to construct pharmacophores by PharmaGist than by the HypoGen model. For each protein, we constructed one PharmaGist model: these models can be used to do virtual screening in the future. PharmaGist is also a user friendly webserver. Please see Additional file 5: Figure S5.6.

### Cocktail multiple-target strategy: A novel model combined with systems biology and CADD

In summary, we used systems biology to construct the common core network marker of four different cancers, which contains 28 proteins as the house-keeping proteins shared by the different cancers. Then we used the docking and pharmacophore methods to perform virtual screening on these 28 core proteins and get the top compounds for each protein. In contrast to traditional single target methods, we suggest using a combination of the top compounds to perform cell proliferation experiments. We have provided an example for each of our first three approaches. Our research provides a novel direction for target therapy for cancers. Wet-lab biomedical scientists can combine the top ligands for each of the 28 core proteins based on their experimental conditions and the pathways that they want to focus on. As an early stage project, we have taken a conservative approach, only using the NCI anti-cancer drug library. As these drugs have proven anti-cancer activity, our cocktail of multiple drugs can be expected to slightly or moderately enhance the therapeutic effect under the right wet-lab conditions. Collaboration with and input from wet-lab experts would permit the modification and optimization of our model, and the scope could expand to include other drug libraries in the virtual screening.

### Biological Experimental Validations

The detailed protocol for the biological experiment in this Part, please refer to our previous work [36]. And the results please refer to Additional file 8. Recently, our team members who are part of wet-lab experiments have found that: if we used more than one drug to attack more than one target at the same time, it will decrease the IC_50 _of the drug. Our team has shown that if we used Gefitinib (Iressa) and L4 (one drug from the LOPAC drug library) to inhibit Src and EGFR at the same time, we achieve lower cell viability.

## Results and Discussion

### Review of the results of our previous methods

Our previous study identified 28 core proteins in the common core network marker for four cancers [17]. These are the proteins intersecting between the four cancers' PPI networks with the top CRVs. Figure 1(a) shows the PPI information of each common core network marker. As stated previously, these 28 core proteins could be responsible for the house-keeping mechanisms of these four cancers, so using the minimum possible number of ligands to target the maximum possible number of the 28 proteins could be the most efficient strategy to attack the cancers.

### Several approaches to break the cancer core network marker

a. We chose the top 20 compounds with the highest docking scores for each of the 28 significant proteins. Of a total of 560 ligands (Additional file 4: Table S2), we found there were 13 ligands that target 2 or 3 proteins simultaneously (Table 4(a)) after redundancy analysis. These 13 ligands combined 11 target proteins (Table 5new 3(b)). We used the 13 ligands as the minimum package for breaking the core network by inhibiting these 11 proteins. While this does not target all 28 proteins, the main purpose here was to minimize the number of ligands at this first stage trial. (As we have said, this is the first trial of our primary model.) Of course, according to our docking results of these 28 proteins, there are thousands of possible drug combinations that can be used based on different analysis methods as per a given researcher's scope. It is quite possible to develop combinations to target all the 28 proteins based on our docking results.

**Table 4:** NCI drugs target more than one protein.

NO.	NCI Drug	Protein Name	LibDock Score
1	**625439**	**KIAA0101**	**145.708**
	
	**625439**	**HDAC1(4BKX)**	**134.097**
	
	**625439**	**HDAC4(2VQM)**	**149.324**

2	**645378**	**CEBPB(2E_42)**	**158.637**
	
	**645378**	**KIAA0101**	**140.103**

3	**668448**	**BRCA1(4IFI)**	**192.069**
	
	**668448**	**PSMA3**	**166.516**

4	**668577**	**MYC(1NKP)**	**125.603**
	
	**668577**	**PSMA3**	**180.11**

5	**682094**	**TERF1(3BQO)**	**123.287**
	
	**682094**	**YWHAZ(4HKC)**	**182.534**

6	**687363**	**MYC(1NKP)**	**145.138**
	
	**687363**	**YWHAZ(4HKC)**	**184.649**

7	**695175**	**TRAF2(1D0A)**	**101.267**
	
	**695175**	**PSMA3**	**148.83**

8	**695409**	**TP53(1TSR)**	**125.821**
	
	**695409**	**PSMA3**	**164.906**

9	**704565**	**KIAA0101**	**137.38**
	
	**704565**	**HDAC4(2VQM)**	**153.564**

10	**719660**	**MYC(1NKP)**	**134.021**
	
	**719660**	**YWHAZ(4HKC)**	**200.903**

11	**724305**	**CEBPB(2E_42)**	**167.724**
	
	**724305**	**HDAC4(2VQM)**	**141.195**

12	**726771**	**BRCA1(4IFI)**	**142.956**
	
	**726771**	**HDAC4(2VQM)**	**148.366**

13	**742856**	**MYC(1NKP)**	**140.093**
	
	**742856**	**TP53(1TSR)**	**100.787**

**Table 5:** The proposed 13 drugs totally target the following 11 proteins

**No**.	Protein Name
**1**	**BRCA1(4IFI)**

**2**	**CEBPB(2E42)**

**3**	**ISG15(3SDL)**

**4**	**MDM2(4MDN)**

**5**	**PCNA(3WGW)**

**6**	**PRKDC(3KGV)**

**7**	**PSMA3**

**8**	**SRC(2SRC)**

**9**	**TERF1(3BQO)**

**10**	**TP53(1TSR)**

**11**	**XRCC6(1JEQ)**

b. In our previous research, we observed the CRVs of the 28 core proteins for each of the four cancers (Table 2). In this study, we summed up the 4 CRVs for each of the 28 proteins. After ranking these summations, we listed the top 5 CRV summations (Table 2), and suggested them to be the therapeutic targets for breaking cancer core network marker. According to our previous systems biology analysis, we believe that inhibiting proteins with the highest CRVs is a highly efficient way to attack cancers. As the first trial, our combination of proteins with the top 5 CRVs should simply be taken as a proof of concept: one can develop combinations according to one's particular needs based on our CRVs.

c. We have also listed pathway analyses using Panther and MetaCore on the core network proteins. Other scientists can use this information to choose the pathways that they want to target, and then choose the best combination of drugs from our virtual screening results. For instance, from the pathway analysis results using Panther (Table 6), researchers interested in targeting the p53 pathway can choose between EP300, CREBBP, HDAC1, HDA2, TRAF2, CDK2, MDM2, and TP53 as the therapy targets. For the above scenario, researchers can choose the top drugs from Additional file 4: Table S2. MetaCore gives us more information for the purpose of target therapy. The top three modules mapped to our 28 core proteins are listed in Table 7. Taking the first map/module (DNA damage_ATM/ATR regulation of G1/S checkpoint) as an example, we see CDK2, p53, Ubiquitin, BRCA1, c-Myc, PCNA, and MDM2 are related to this module. As above, we can use the drugs in Additional file 4 : Table S2 to choose the optimal combination of drugs to attack this module.

**Table 6:** the pathway analysis results of Panther.

Rank	Pathway title	Count	Gene
1	p53 pathway	8	EP300,CREBBP,HDAC1,HDA2,TRAF2,CDK2,MDM2,TP53
2	Wnt signaling pathway	7	EP300,CREBBP,HDAC1,MYC,HDAC2,CTNN1,TP53
3	p53 pathway feedback loops 2	6	MYC,RB1,CTNNB1,CDK2,MDM2,TP53
4	Parkinson disease	4	YWHAZ, CUL1,PSMA3,SRC
4	Gonadotropin releasing hormone receptor pathway	4	EP300,CREBBP,CTNNB1, SRC
5	Huntington disease	3	EP300,CREBBP,TP53
6	Apoptosis signaling pathway	2	TRAF2,TP53
6	Angiogenesis	2	CTNNB1, SRC
6	P53 pathway feedback loops 1	2	MDM2, TP53
6	Cadherin signaling pathway	2	CTNNB1, SRC
6	Transcription regulation by bZIP transcription factor	2	EP300, CREBBP
6	TGF-beta signaling pathway	2	EP300, CREBBP
7	Interleukin signaling pathway	1	MYC
7	Alzheimer disease-presenilin pathway	1	CTNNB1
7	Integrin signalling pathway	1	SRC
7	Insulin/IGF pathway-protein kinase B signaling cascade	1	MDM2
7	Hypoxia response via HIF activation	1	CREBBP
7	Ubiquitin proteasome pathway	1	MDM2
7	p53 pathway by glucose deprivation	1	TP53

**Figure 2:**
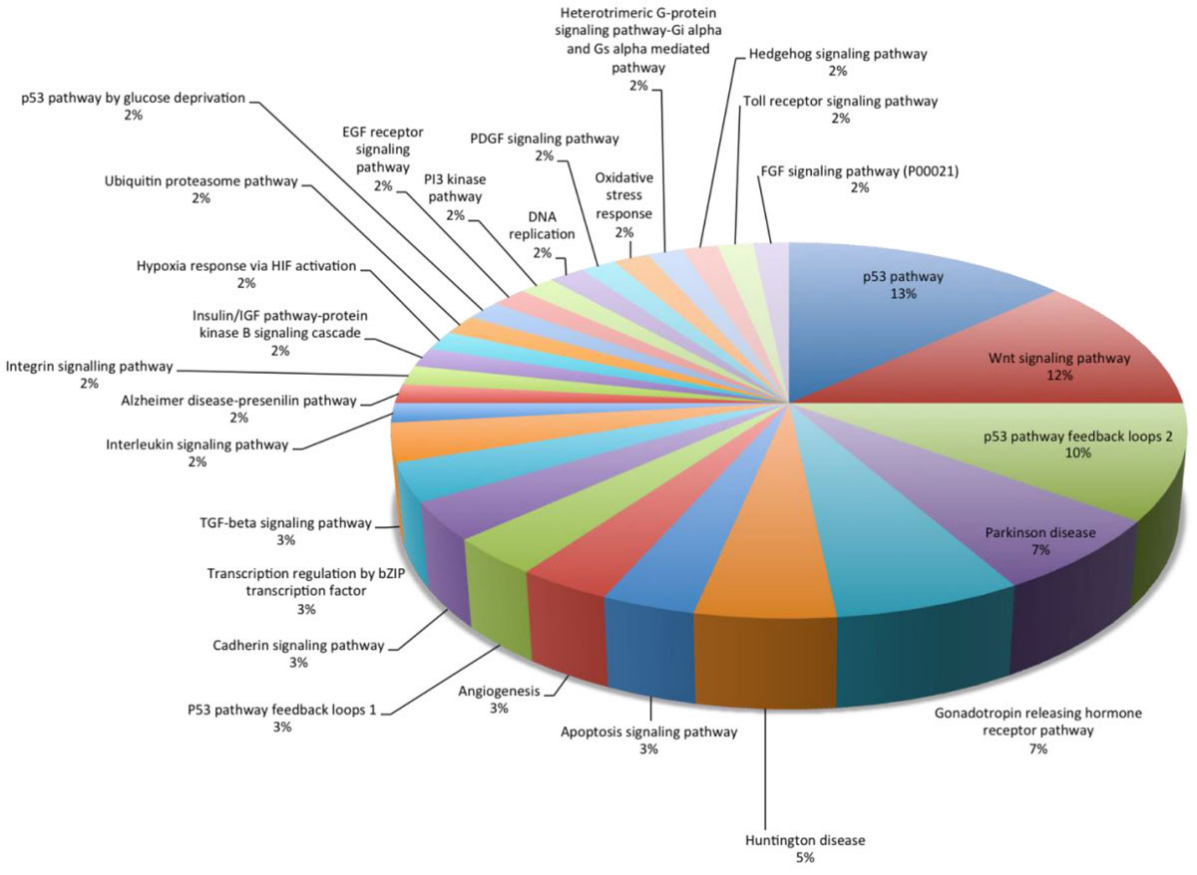
**Pathway analysis by Panther**. We see the 28 proteins of core network marker can hit many important cancer related pathways, such as p53, Wnt signalling, p53 feedback loops 2, apoptosis, *etc*. As we have described above, this study is only a prototype model. By the Panther analysis, we could know to inhibit the right proteins which hit the key pathways and is one of the important strategies in the real clinical situation. This result offers another reference for doctors to design the best combination of multiple inhibitors. We will do more deep analysis in the future if more clinical doctors could offer the clinical data to us. The preliminary results also could help us exclude the cancer-unrelated pathways at the first stage, such as Hungtingon disease, alzeheimer, *etc*.

Please see their statistical distribution in Figure 2.

**Table 7:** Top three modules/maps given by Metacore.

No	Processes	Map of core proteins
1	**DNA damage_ATM/ATR regulation of G1/S checkpoint: **It is the highest scoring map (i.e., the map with the lowest p value). ATM/ATR regulates both the checkpoints of the G1/S and S/G2. DNA damage checkpoints pathways arrest or delay the progression of cell cycle in response to the DNA damage. Eukaryotic cell cycle have four phases, G1 (G indicating gap), S (Synthesis), G2 (**Gap 2**), and M (Mitosis), and one outside, G0 (Gap 0).	CDK2, p53, Ubiquitin, BRCA1, c-Myc, PCNA, MDM2

2	**Transcription_P53 signaling pathway: **It is the second highest scoring map (i.e., the map with the second lowest p-value). The Tumor protein p53, also known as p53 ortransformation-related protein 53 (TRP53), plays a significant role in shielding the genome integrity. While being activated, p53 will bind to the enhancer/promoter regions of downstream target genes. And then it regulates the transcription of these genes, through which it initiates cellular processes that responsible for lots of its tumor-suppressor related functions. It is not surprising that core network of 4 cancers are related to the p53 signaling pathway.	CDK2, p53, CBP, p300, Rb protein, MDM2, Beta-catenin

3	**DNA damage_BRCA1 as a transcription regulator: **It is the third highest scoring map (i.e., the map with the third lowest p-value). Activation of breast cancer susceptibility gene 1 (BRCA1) by DNA damage occurs via activating the ataxia telangiectasia mutated serine/threonine protein kinase (ATM) or serine/threonine-protein kinase ATR (ataxia telangiectasia and Rad3 related protein kinase). These protein kinases can either directly or indirectly phosphorylate BRCA1 (by cell cycle checkpoint kinase 2 (Chk2)). BRCA1 acts a significant role in the DNA repair process by expediting cellular response upon DNA repair. There are numerous DNA repair pathways (see map 427 Role of BRCA1 and BRCA2 in DNA repair). We know that DNA repair is highly related to various cancers.	p53, ESR1 (nuclear), BRCA1, c-Myc, PCNA, Rb protein

### More pathway and functional analysis

A. Panther

The results of Panther are shown in Figure 2 and listed in Table 6. We see the 28 core network markers hit many important cancer-related pathways, such as p53, Wnt signaling, p53 feedback loops 2, apoptosis, *etc*. As we have described above, this study is a prototype model. Inhibition of the right proteins hitting the key pathways is an important strategic consideration in real clinical situations. Our results offer another reference for doctors to design the best combination of multiple inhibitors. In the future, using clinical data from doctors will help us perform deeper analysis. These preliminary results also could help us exclude pathways unrelated to cancer at the first stage, such as those related to Huntington's disease, Alzheimer's disease, *etc*.

B. MetaCore

The results of MetaCore analysis are described below. We have described the top three maps/modules (Table 7).

**Figure 3:**
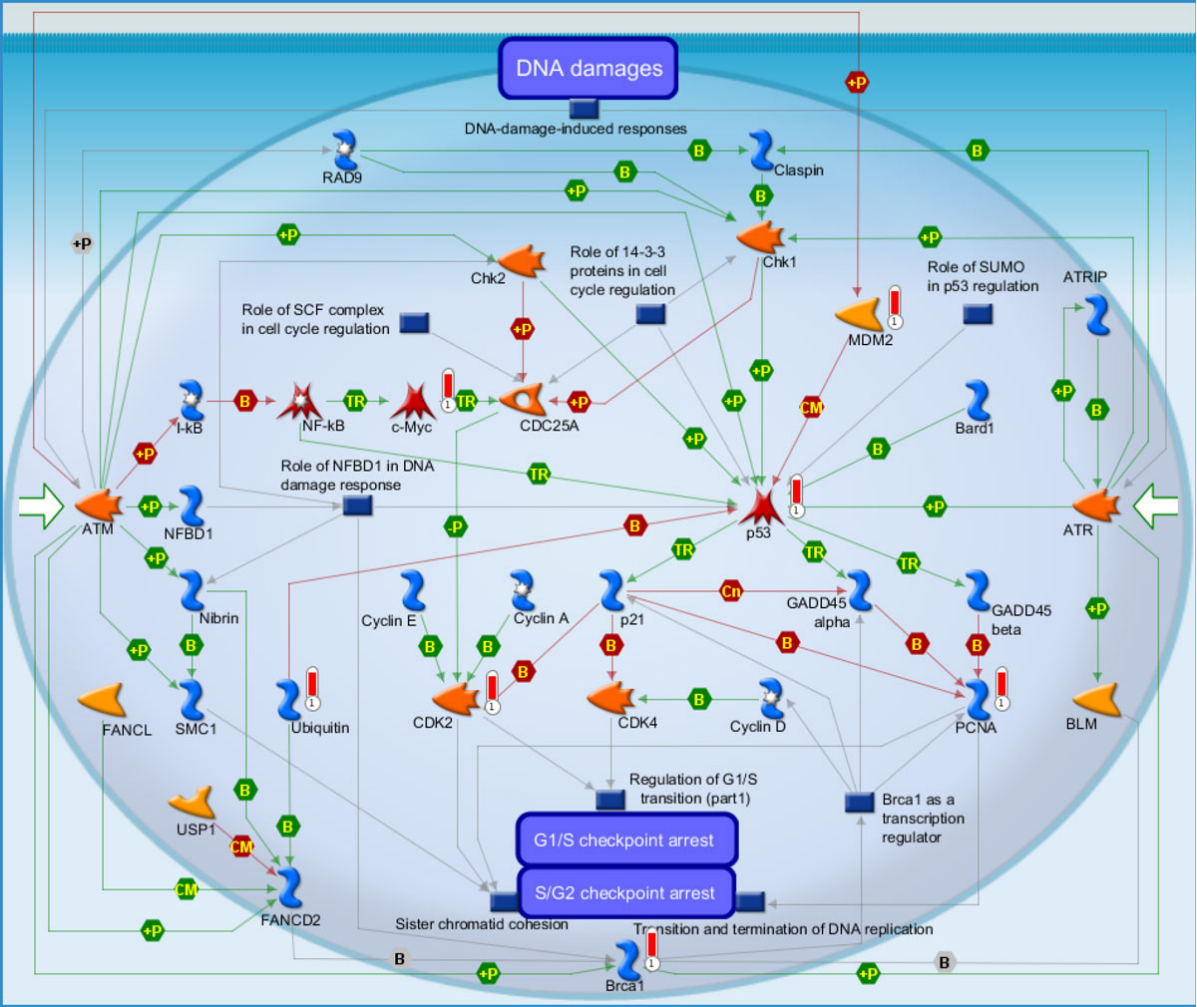
**DNA damage_ATM/ATR regulation of G1/S checkpoint**. It is the highest scoring map (i.e., the map with the lowest p value). ATM/ATR regulates both the checkpoints of the G1/S and S/G2. DNA damage checkpoints pathways arrest or delay the progression of cell cycle in response to the DNA damage. Eukaryotic cell cycle have four phases, G1 (G indicating gap), S (Synthesis), G2 (Gap 2), and M (Mitosis), and one outside, G0 (Gap 0).

**Figure 4:**
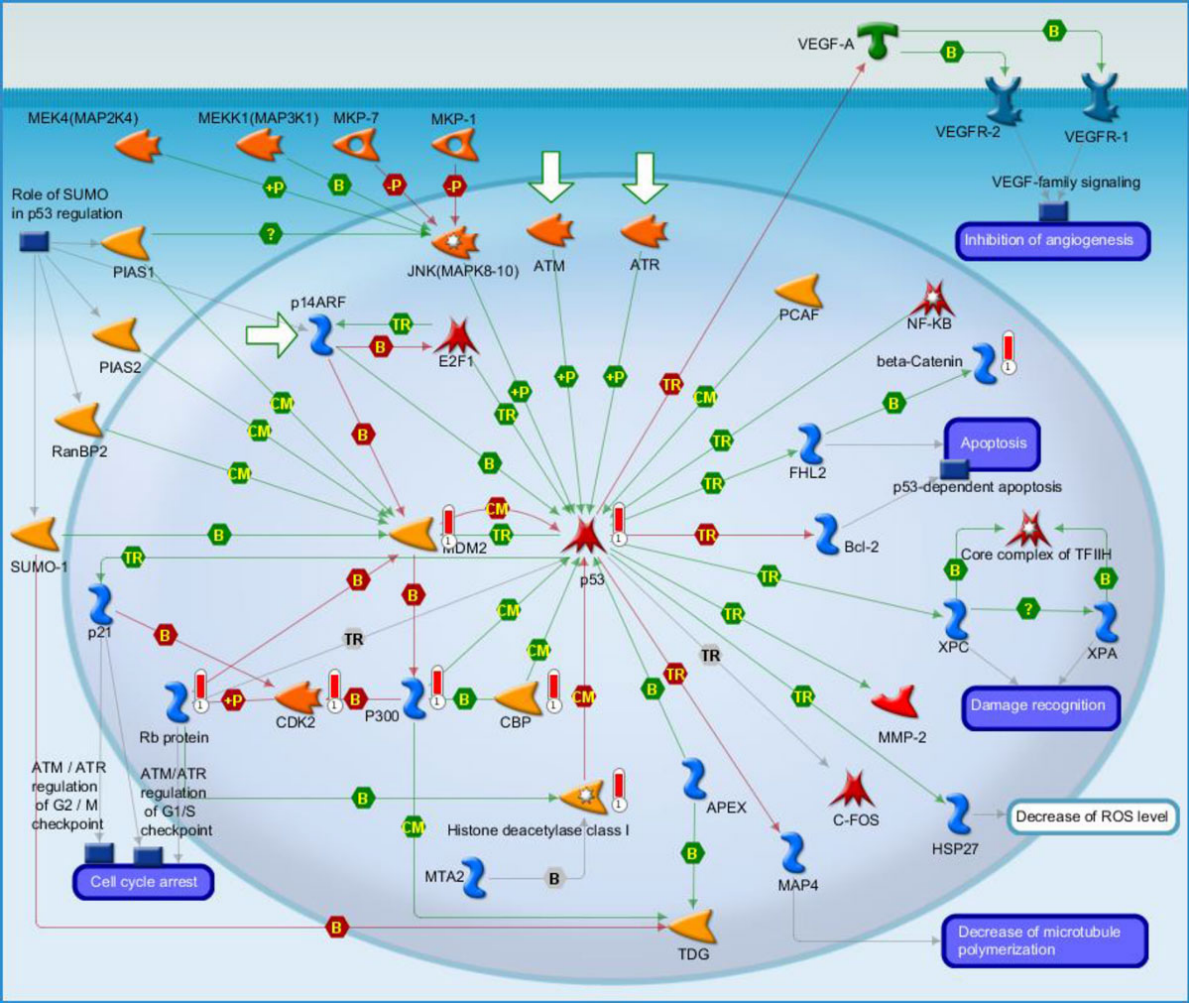
**Transcription_P53 signaling pathway**. It is the second highest scoring map (i.e., the map with the second lowest p-value). The Tumor protein p53, also known as p53 ortransformation-related protein 53 (TRP53), plays a significant role in shielding the genome integrity. While being activated, p53 will bind to the enhancer/promoter regions of downstream target genes. And then it regulates the transcription of these genes, through which it initiates cellular processes that responsible for lots of its tumor-suppressor related functions. It is not surprising that core network of 4 cancers are related to the p53 signaling pathway.

**Figure 5:**
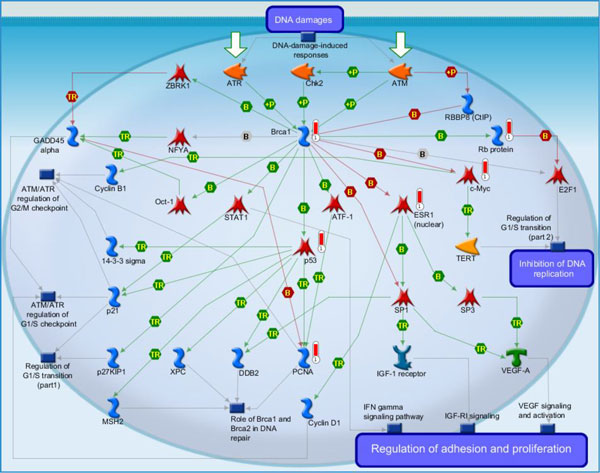
**DNA damage_Brca1 as a transcription regulator**. It is the third highest scoring map (i.e., the map with the third lowest p-value). Activation of breast cancer susceptibility gene 1 (BRCA1) by DNA damage occurs via activating the ataxia telangiectasia mutated serine/threonine protein kinase (ATM) or serine/threonine-protein kinase ATR (ataxia telangiectasia and Rad3 related protein kinase). These protein kinases can either directly or indirectly phosphorylate BRCA1 (by cell cycle checkpoint kinase 2 (Chk2)). BRCA1 acts a significant role in the DNA repair process by expediting cellular response upon DNA repair. There are numerous DNA repair pathways (see map 427 Role of BRCA1 and BRCA2 in DNA repair). We know that DNA repair is highly related to various cancers.

(i) DNA damage_ATM/ATR regulation of G1/S checkpoint (Figure 3): It is the highest scoring map (i.e., the map with the lowest p value). ATM/ATR regulates both the checkpoints of the G1/S and S/G2. DNA damage checkpoints pathways arrest or delay the progression of cell cycle in response to the DNA damage. Eukaryotic cell cycle have four phases, G1 (G indicating gap), S (Synthesis), G2 (Gap 2), and M (Mitosis), and one outside, G0 (Gap 0) [37]. When DNA damage occurs, the G1/S checkpoints will inhibit the initiation of replication to prevent cells from entering the S phase. They are related to two pathways of signal transduction, to initiate and maintain the G1/S arrest, respectively [37]. Jiri Bartek *et al*. discussed "The DNA damage response in tumorigenesis and the treatment of cancer" [38]. [The above description is directly cited from the Metacore document.]

(ii) Transcription_P53 signaling pathway (Figure 4): It is the second highest scoring map (i.e., the map with the second lowest p-value). The Tumor protein p53, also known as p53 ortransformation-related protein 53 (TRP53), acts a significant role in shielding the genome integrity. While being activated, p53 will bind to the enhancer/promoter regions of downstream target genes. And then it regulates the transcription of these genes, through which it initiates cellular processes that responsible for lots of its tumor-suppressor related functions [39]. It is not surprising that core network of 4 cancers are related to the p53 signaling pathway. [The above description is directly cited from the Metacore document.]

(iii) DNA damage_Brca1 as a transcription regulator (Figure 5): It is the third highest scoring map (i.e., the map with the third lowest p-value). Activation of breast cancer susceptibility gene 1 (BRCA1) by DNA damage occurs via activating the ataxia telangiectasia mutated serine/threonine protein kinase (ATM) [40] or serine/threonine-protein kinase ATR (ataxia telangiectasia and Rad3 related protein kinase) [41]. These protein kinases can either directly or indirectly phosphorylate BRCA1 (by cell cycle checkpoint kinase 2 (Chk2) [42]). BRCA1 acts a significant role in the DNA repair process by expediting cellular response upon DNA repair. There are numerous DNA repair pathways (see map 427 Role of BRCA1 and BRCA2 in DNA repair) [43]. We know that DNA repair is highly related to various cancers. [The above description is directly cited from the Metacore document.]

* The highest three scoring map (i.e., the map with the three lowest p-value) is based on the enrichment distribution sorted by the 'Statistically significant maps' set. Experimental data from all files is linked to and visualized on the maps as thermometer-like figures. Up-ward thermometers are red and indicate up-regulated signals, and down-ward (blue) ones indicate down-regulated expression levels of the genes. [The above description is directly cited from the Metacore document.]

Statistical analysis results of the three maps are shown in Table 7. These results also help us choose more suitable targets and exclude less suitable ones.

### Protein and ligand structures

Table 1 shows detailed information of these 28 proteins including protein name, PDB ID, resolution, chains, length, ligands, and their full names. Most of these 3D structures were downloaded from the PDB database. 3D structures of CEBPB, IRAK1, KIAA0101 and PRKDC were constructed by homology modeling using I-TASSER. We can see that many protein structures also contained a ligand in a bound conformation. We used the locations of these ligands within the target protein as the docking site. Binding sites of proteins without bound ligands were predicted using COACH.

### Docking

**Figure 6:**
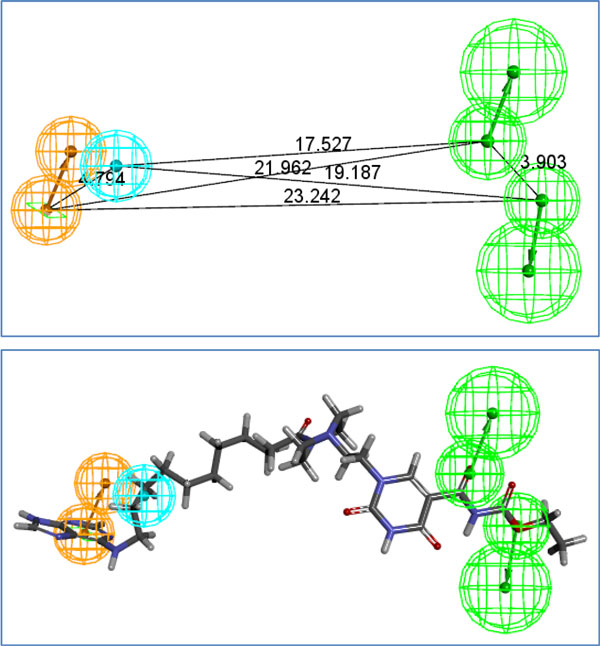
**new 8- The hypogen pharmacophore model built by DS3**.5. We show the distances between the pharmacophores. No. 625869 is the drug in NCI anti-cancer library screened and matched by this pharmacophore.

Figure 6 Additional file 9 shows the docking pose of the top compounds (i.e., those with the highest docking score). Their docking scores ranged from 95 to 241. The analysis also shows the key residues in ligand binding. Our study is the first to perform high-throughput multiple-protein docking, and this work requires large computational resources. Due to space limitations, we have not listed another table for these key residues. The information could be useful for future drug design and these top compounds could be considered as control ligands.

### Pharmacophore

3D pharmacophore modeling is another powerful method to perform virtual screening on large ligand databases. It is as powerful as the docking method, and is always more efficient than docking methods. For example, the large ligands database ZINC [44] has more than 200 million ligands. By calibrating the parameters of the pharmacophore virtual screening, it is possible to virtually screen the whole ZINC database using our IBM server with 160 cores. We have even performed virtual screening for Src inhibitors. However, constructing a proper HypoGen model is also very time-consuming. As an initial study using a novel approach, we used relatively unrestricted conditions to set up a rough HypoGen model. It would be beneficial to do virtual screening on another ligand database in the future.

**Figure 7:**
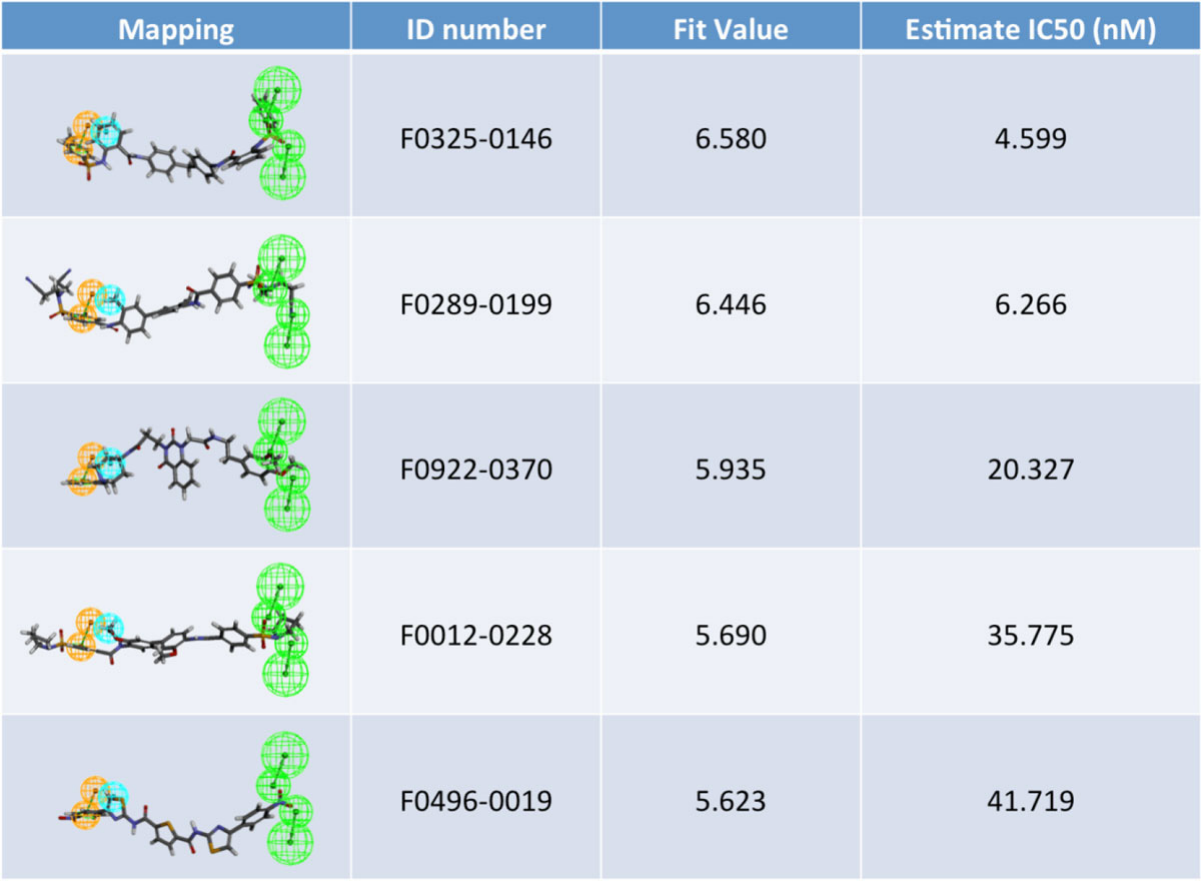
**new 9- Life-Chemical drugs screened out by Hypogen**. These are the top ligands of Life-Chemical drug library screened by our hypogen pharmacophore model. Please see Table 10 for the detailed descriptions of these mapping results.

**Figure 8:**
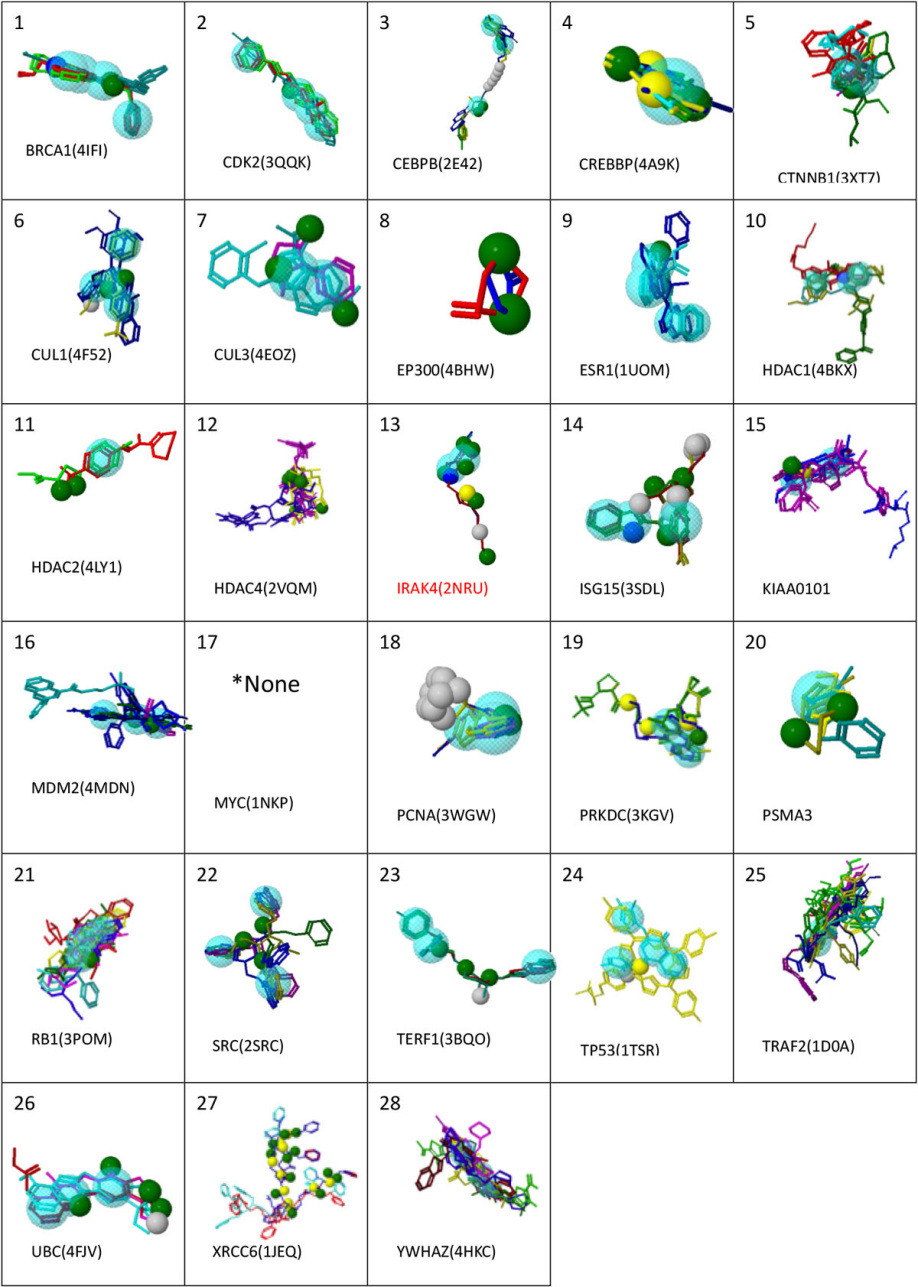
**new 10-PharmGist Pharmacophore Model**. The pharmacophores for each of the 28 significant proteins built by PharmGist. Detailed descriptions of these pharmacophores are in Tables 7-9. [*17:MYC(1NKP)'s pharmacophore could not be generated.]

Figure 7 Additional file 10 lists the total 54 ligands used to construct the HypoGen pharmacophore model. We present the structure of the pharmacophore in Figure 6new, where NSC-625869 refers to the ligand in the NCI library of anti-cancer drugs that was screened and matched by this pharmacophore. The numerical results of the HypoGen model are shown in Tables 8, 9, 10. After having constructed this pharmacophore, we were able to use it to screen against other ligand databases, such as the Life Chemical database. Figure 7 and Table 11new show the compounds in the Life Chemical database screened and matched by this pharmacophore. Compared to the HypoGen model, the pharmacophore model derived by PharmGist is easier to obtain because PharmGist does not require the IC_50 _values of the compounds for computation. We have listed the results in Figure 8 and Table 12.

**Table 8:** Top pharmacophore description

Category	Number of ligands	IC_50_ range (nM)	Feature	Correlation
28 targets	54	2-50000	HBA, HBA, HY, RA	0.92

**Table 9:** Top 10 pharmacophore descriptions

Hypothesis	Total cost	ΔCost	RMS	Correlation (r)	Features
1	211.414	33.787	0.583549	0.924995	HBA, HBA, HY, RA
2	211.745	33.456	0.599912	0.920410	HBA, HBA, HY, RA
3	212.295	32.906	0.606595	0.918758	HBA, HBA, HY, RA
4	212.530	32.671	0.609627	0.917975	HBA, HBA, HY, RA
5	213.564	31.637	0.641692	0.908625	HBA, HBA, HY, RA
6	213.870	31.331	0.651935	0.905497	HBA, HBA, HY, RA
7	214.412	30.789	0.669021	0.900153	HBA, HBA, HY, RA
8	215.039	30.162	0.690558	0.893115	HBA, HBA, HY, RA
9	215.282	29.919	0.694361	0.891929	HBA, HBA, HY, RA
10	215.758	29.443	0.713440	0.885327	HBA, HBA, HY, RA

* Null cost = 245.201; Fixed cost = 202.014; Configuration cost = 19.2545; ΔCost = Null cost - Total cost. All costs are in units of bits.

* HBA, hydrogen bond acceptor; HY, hydrophobic; RA, ring aromatic.

**Table 10:** Experimental and estimation values of IC_50 _for each of the 54 ligands

NCI Drug	IC_50 _(nM)	Error	NCI Drug	IC_50 _(nM)	Error		
	Exp.	Est.			Exp.	Est.	

							
625869	2.0	2.4	1.2	669299	4792.2	5656.4	1.2
668448	66.3	142.2	2.1	641847	5026.9	4480.2	-1.1
698233	256.5	97.5	-2.6	669230	5282.5	4302.8	-1.2
668433	380.2	229.7	-1.7	657381	5358.4	4451.3	-1.2
737026	810.9	366.4	-2.2	643737	5376.8	4463.6	-1.2
654626	1377.0	1868.5	1.4	691369	5509.1	4822.7	-1.1
726771	1877.1	4272.0	2.3	627399	5527.5	4285.1	-1.3
717079	2067.7	4413.1	2.1	627727	5892.9	14154.0	2.4
674086	2234.0	4138.9	1.9	693235	6107.2	5310.1	-1.2
666346	2270.0	5579.5	2.5	675823	6377.0	5049.0	-1.3
740601	2627.9	4280.5	1.6	665675	6591.5	4380.4	-1.5
639795	2742.6	4358.1	1.6	295632	7254.4	4402.1	-1.6
673172	2823.0	4721.0	1.7	668373	7390.1	4343.3	-1.7
641236	2827.4	4418.8	1.6	749	7534.5	21062.2	2.8
76955	2836.7	4936.6	1.7	689447	8003.5	5397.9	-1.5
719481	3033.7	6903.6	2.3	202537	12700.6	21711.5	1.7
406433	3064.1	12371.9	4.0	639906	13142.7	5880.0	-2.2
683481	3344.0	4540.1	1.4	622153	14753.4	5742.3	-2.6
682236	3395.0	4677.1	1.4	622154	15831.2	4771.3	-3.3
661908	3469.7	4310.7	1.2	692400	50000.0	13059.1	-3.8
633409	3661.4	4382.2	1.2	687325	50000.0	14121.3	-3.5
672968	3845.3	4363.1	1.1	689530	50000.0	17997.5	-2.8
680359	4140.3	4290.9	1.0	691200	50000.0	21974.7	-2.3
76519	4334.6	3133.5	-1.4	684143	50000.0	32999.6	-1.5
702125	4732.2	4288.4	-1.1	683630	50000.0	40400.2	-1.2
678636	4732.9	12631.9	2.7	694265	50000.0	51746.7	1.0
682086	4770.1	4479.7	-1.1	695571	50000.0	61122.2	1.2

*Exp= Experimental value; Est=Estimation value by pharmacophore; Error=Est./Exp.

**Table 11:** The Life-Chemical ligands are virtually screened by the hypogen model.

**ID No**.	FitValue	Estimate	HBA_1	HBA_2	HY_3	RA_4
F0325-0146	6.580	4.599	1	1	1	1
F0289-0199	6.446	6.266	1	1	1	1
F0922-0370	5.935	20.327	1	1	1	1
F0012-0228	5.690	35.775	1	1	1	1
F0496-0019	5.623	41.719	1	1	1	1
F0737-0405	5.55006	49.3217	1	1	1	1
F0382-0020	5.53538	51.0174	1	1	1	1
F0325-0148	5.1207	132.555	1	1	1	1
F0737-0312	5.00395	173.442	1	1	1	1
F0725-0356	4.98991	179.137	1	1	1	1
F0301-0263	4.9208	210.04	1	1	1	1
F0473-0314	4.90716	216.74	1	1	1	1
F0922-0913	4.87165	235.207	1	1	1	1
F1601-0068	4.58408	456.055	1	1	1	1
F0463-0195	4.50862	542.605	1	1	1	1
F0866-0317	4.26226	956.832	1	1	1	1
F0537-0936	4.17732	1,163.54	1	1	1	1
F0180-0144	3.96884	1,880.45	1	1	1	1
F0922-0900	3.95736	1,930.82	1	1	1	1

Please see the mapping diagrams in Figure 11.

**Table 12:** The pharmacophores descriptions of PharmGist.

**No**.	Protein	PDB	Score	Features	Spatial Features	R	H	D	A	N	P	Chemicals	Fig
1	BRCA1	4IFI	39.2	7	7	4	0	0	2	0	1	3	9-1
2	CDK2	3QQK	33.1	5	5	4	0	0	1	0	0	3	9-2
3	CEBPB	2E42	40.4	12	12	3	5	0	4	0	0	3	9-3
4	CREBBP	4A9K	29.9	5	5	1	0	2	2	0	0	9	9-4
5	CTNNB1	3TX7	23.7	3	3	2	0	0	1	0	0	7	9-5
6	CUL1	4F52	37.5	7	7	4	1	0	2	0	0	3	9-6
7	CUL3	4EOZ	25.7	5	5	2	0	0	3	0	0	3	9-7
8	EP300	4BHW	3.0	2	2	0	0	0	2	0	0	2	9-8
9	ESR1	1UOM	33.1	5	5	4	0	0	1	0	0	3	9-9
10	HDAC1	4BKX	26.5	4	4	3	0	0	0	0	1	4	9-10
11	HDAC2	4LY1	6.0	3	3	1	0	0	2	0	0	2	9-11
12	HDAC4	2VQM	34.0	7	5	0	0	2	5	0	0	3	9-12
13	IRAK4	2NRU	44.5	13	11	2	1	3	6	0	1	3	9-13
14	ISG15	3SDL	42.7	16	14	3	7	2	3	0	1	3	9-14
15	KIAA0101		26.5	4	4	2	0	1	1	0	0	3	9-15
16	MDM2	4MDN	36.0	5	5	3	0	0	2	0	0	6	9-16
17	MYC	1NKP											9-17
18	PCNA	3WGW	36.0	14	14	3	9	0	2	0	0	3	9-18
19	PRKDC	3KGV	36.7	7	7	3	0	3	1	0	0	3	9-19
20	PSMA3		14.7	3	3	1	0	0	2	0	0	3	9-20
21	RB1	3POM	36.4	4	4	3	0	0	1	0	0	9	9-21
22	SRC	2SRC	35.7	6	6	3	0	0	3	0	0	4	9-22
23	TERF1	3BQO	36.5	7	7	3	1	0	3	0	0	4	9-23
24	TP53	1TSR	33.8	6	6	4	1	1	0	0	0	3	9-24
25	TRAF2	1D0A	30.9	3	3	2	0	0	1	0	0	16	9-25
26	UBC	4FJV	41.6	10	9	3	1	1	5	0	0	3	9-26
27	XRCC6	1JEQ	141.1	22	22	0	0	10	12	0	0	3	9-27
28	YWHAZ	4HKC	34.9	3	3	3	0	0	0	0	0	12	9-28

Their pharmacophore diagrams are shown in Figure 7.

### Biological Experimental Validations

Drug combination- Gefitinib (Iressa) combine with L4 and N4 in three different cells. In the three cell lines, they show that combination drug always get a better efficiency that only single Iressa. Please refer to Additional file 8. At this stage, we have only used a simple pathway diagram to visualize the relationship between EGFR and Src without a large-scale systems biological analysis. These results gave us confidence that multiple drugs attacking multiple targets will achieve better results. Indeed, we have accumulated lots of similar results.

### Novel model combined with both systems biology and CADD

**· General demonstration: **Our method has reversed the normal procedure of CADD with the help of systems biology. In the traditional CADD method, a single validated target, such as Src, EGFR, and FAK, is used as the protein target. The first step is usually virtual screening by docking or the pharmacophore method. The second step is to validate the top compounds from virtual screening with ELISA or Western blot wet-lab experiments. If they are found to bind to the target proteins, then researchers could employ these ligands in cell proliferation experiments. For our novel approach, we choose the top compounds from several targets simultaneously, and then perform the cell proliferations experiment at the beginning. Our hypothesis is that these drug cocktails would destroy the core network, although we have not experimentally validated whether each top compound binds to its corresponding target or not

**· Time complexity versus space complexity: **Our novel approach is some kind like the problem of algorithm about the time complexity and space complexity. To attack each protein one by one is similar to the problem of time complexity. This is the traditional strategy for single target drug design. The traditional method needs to make sure the drugs target to which protein exactly. In the language of computer science, the traditional CADD method can only do serial work but not parallelization work. By the help of our systems biology approach, it turns the origin problem from time complexity to space complexity. That is, you can do parallelization attack to the multiple proteins simultaneously. The powerful of this parallelization attack is that you can do various drugs combination trial at the short time. When you get the better results, you can go back to check each drug in the combination is useful for the proteins individually or not. Honest to speak, there are possible too many uncertainties and inaccuracies in this model, such as the network biomarkers predicted by our systems biology approach, the binding site information, the pharmacophore model or even the predicted 3D protein structures. However, due to the powerful of parallelization attack (cocktail multiple drugs), it is possible to find the useful cocktail combination under the situation of so many uncertainties and inaccuracies. The problem of space complexity we encounter now is that how many drugs we could use at the same time. And this is our feature work.

**· Welcome to asking for our help and cooperation: **We must emphasize that no matter how powerful of our model, the most important thing is to combine with the correct medical knowledge and intuition. After our model gives so many possible combinations of drug, you have to decide a set of best ones with your medical knowledge and intuition to do the wet-lab experiment validation. We already have a lot of these wet-lab results but not published yet. Our teams also have abundant experience on the single target drug design [45-47]. We have also built a webserver of the systems biology model in the research but not opened to public yet. It is welcome for your feedback and cooperation. You are welcome to modify the old version source code of our systems biology model [48]. Please refer to Additional file 8.

**· Novelty and expectation results in the future: **Combination drugs is not a novel idea [49,50]. To design combination drugs by our systems biology and CADD methods is a novel work. We expect many combination drugs will be really useful by the help of model. We would like to enhance and modify our model in the future.

## Availability of this method

We are in the process of building a webserver to identify network biomarkers. At this stage, we can construct the network on request. We also offer a working version of the source code (Additional File 11), and the readers can modify this version of source code to perform their research experiments.

## Conclusions

In this study, we combined systems biology with traditional computational-aided drug design to design drug cocktails to inhibit the 28 proteins that comprise the common core network markers of four cancers. The results of our previous study have led us to believe that those proteins likely represent house-keeping proteins of these four cancers. Wet-lab researchers could use the cocktails of multiple drugs indicated by our analysis as objects of study in experiments on treating the four cancers. Moreover, with the help of sensitivity analysis, we also found the most likely multiple drug targets for each individual cancer.

## Competing interests

The authors declare that they have no competing interests.

## Authors' contributions

BSC and YHW designed and conduct the theory and experiments of systems biology. CWL modified the core network. JC, YHW, CLL and TSC design and perform the CADD. JC, CAC, PSJ and YHL design and perform the wet-lab biological experiments. LJC perform the pathway and functional analysis. BSC and YHW integrated the whole work. YHW drafted the manuscript. All of the authors have approved the publication of the manuscript.

## Supplementary Material

Additional file 1 **Parameter Identification of PPI network by Maximum Likelihood Method**. Click here for file

Additional file 2 **Determination of significant protein associations by AIC and Student's t-test**. Click here for file

Additional file 3 **The identified significant proteins in 4 cancers**. Click here for file

Additional file 4 **Docking results for the top 20 ligands for the 28 proteins studied**. Click here for file

Additional file 5 **new 6: User interface of commercial software and free webserver**. Click here for file

Additional file 6 **new 7: Mathematical foundation of Metacore**. Click here for file

Additional file 7 **new 5: Binding site information for the 28 proteins**. Click here for file

Additional file 8 **new 10: Biological experimental validation**. Click here for file

Additional file 9 **new 8. Docking pose analysis of 28 core proteins**. The docking poses analysis shows the key residues of the proteins which interact with the ligands. The analysis reveals more mechanisms to help us design *de novo *drugs. Click here for file

Additional file 10 **new 9. 54 ligands used for developing the Hypogen model**. Click here for file

Additional file 11 Source code (*.rar). Click here for file

## Declaration

Funding for this publication was provided by the Ministry of Science and Technology, Taiwan through grants MOST-103-2745-E-007-001-ASP, MOST-103-2221-E-038 -013 -MY2, and MOST-104-2218-E-007-021.

This article has been published as part of *BMC Medical Genomics *Volume 8 Supplement 4, 2015: Joint 26th Genome Informatics Workshop and 14th International Conference on Bioinformatics: Medical genomics. The full contents of the supplement are available online at http://www.biomedcentral.com/bmcmedgenomics/supplements/8/S4.
