# Supplementary material for: Multiple target drug cocktail design for attacking the core network markers of four cancers using ligand-based and structure-based virtual screening methods
Source: BMC Med Genomics. 2015 Dec 9;8(Suppl 4):S4. doi: 10.1186/1755-8794-8-S4-S4 (PMC4682379; doi:10.1186/1755-8794-8-S4-S4)
Supplement: Additional file 1 — Parameter Identification of PPI network by Maximum Likelihood Method. [file 1755-8794-8-S4-S4-S1.docx]

**Additional file 1**

## Parameter Identification of PPI network by Maximum Likelihood Method

Equation (1) can be written as the following requiring form

(9)

where denotes the regression vector which can be obtained from microarray data, $\theta_{i}$is the parameter vector to be estimated. Suppose that there are *m* samples, then it is easy to acquired values of for $n\in\left\{\begin{matrix} 1, & \ldots& ,m \end{matrix}\right\}$. In this case, equation (2) for different samples can be represented as the following vector form.

(10) where ,$\theta_{i}=[\alpha_{i1}\cdots\alpha_{iM_{i}}]$

For simplicity, it can be represented as follows.

$X_{i}=\Phi_{i}\cdot\theta_{i}+e_{i}$ (11)

where $e_{i}=[w_{1}(1)\cdots w_{i}(m)]^{T}$.

In equation (11), the noise $e_{i}$ for different samples was regarded as independent random variables of normal distribution with zero mean and unknown variance $\sigma_{i}^{2}$, i.e., $E\left\{e_{i}\right\}=0$, and $\sum_{i}=E\left\{e_{i}e_{i}^{T}\right\}=\sigma_{i}^{2}I$, where *I* is the identity matrix. The probability density function of $e_{i}$is given as follows.

$p(e_{i})=\frac{1}{((2\pi)^{m}\det\Sigma_{i})^{1/2}}\exp(-\frac{1}{2}e_{i}^{T}\sum_{i}^{-1}e_{i})$ (12)

From equation (12), we can obtain the likelihood function

$L(\theta_{i},\sigma_{i}^{2})=p(\theta_{i},\sigma_{i}^{2})=\frac{1}{(2\pi\sigma_{i}^{2})^{m/2}}\exp(-\frac{(X_{i}-\Phi_{i}\theta_{i})^{T}(X_{i}-\Phi_{i}\theta_{i})}{2\sigma_{i}^{2}})$ (13)

Maximum likelihood estimation method aims at finding $\theta_{i}$and $\sigma_{i}^{2}$ to maximize the likelihood function in equation (13). In order to simplify the computation, it is practical to take the logarithm of the likelihood function, which yields the following log-likelihood function:

(14)

where $x_{i}[n]$and are the *n*-th element of$X_{i}$ and$\Phi_{i}$ in (14), respectively.

By the maximum likelihood parameter estimation method, we expect the log-likelihood function to have the maximum at $\theta_{i}=\hat{\theta}_{i}$ and $\sigma_{i}^{2}=\hat{\sigma}_{i}^{2}$. The necessary conditions for the maximum likelihood estimates $\hat{\theta}_{i}$ and $\hat{\sigma}_{i}^{2}$ must conform to the following two equations.

$\begin{matrix} \frac{\partial\log L(\theta_{i},\sigma_{i}^{2})}{\partial\theta_{i}}=0 \\ \frac{\partial\log L(\theta_{i},\sigma_{i}^{2})}{\partial\sigma_{i}^{2}}=0 \end{matrix}$ (15)

The estimated parameters $\hat{\theta}_{i}$ and $\hat{\sigma}_{i}^{2}$ are shown below,

$\hat{\theta}_{i}=(\Phi_{i}^{T}\Phi_{i})^{-1}\Phi_{i}^{T}Y_{i}$ (16)

(17)

where $X_{i}$ and$\Phi_{i}$ can be obtained from the microarray in the rough PPIN. Since there are two data sets of microarray data, two association parameters for cancer and non-cancer were separately identified.
