# Supplementary material for: Multiple target drug cocktail design for attacking the core network markers of four cancers using ligand-based and structure-based virtual screening methods
Source: BMC Med Genomics. 2015 Dec 9;8(Suppl 4):S4. doi: 10.1186/1755-8794-8-S4-S4 (PMC4682379; doi:10.1186/1755-8794-8-S4-S4)
Supplement: Additional file 2 — Determination of significant protein associations by AIC and Student's t-test. [file 1755-8794-8-S4-S4-S2.docx]

**Additional file 2**

## Determination of significant protein associations by AIC and Student’s t-test

When association parameters of all the proteins in rough PPIN were identified as equation (2), significant protein associations were determined by parameter estimates $\hat{\alpha}_{ij}$ of their association abilities. In order to determine whether the association was significant or not, Akaike Information Criterion (AIC) and Student’s t-test, which is used to calculate the *p*-values of the association abilities, are employed to detect the system model order (or the number of model parameters) and determine the significance of our model parameters. The AIC, a method for model order detection, attempts to include both the estimated residual variance and model complexity in one statistic. AIC decreases as residual variance decreases, and increases as the number of parameters increases. As the expected residual variance decreases with increasing parameter numbers for excessive model complexity, a minimum should appear near the correct parameter number. Thus, the AIC criterion, in which estimated parameters were obtained above, was used to select model structure. Due to computation efficiency, it is impractical to compute the AIC statistics for all possible regression models. Here, the stepwise regression method which combines forward selection method and backward elimination method was applied to compute the AIC statistics. Once the estimated association parameters were examined using the AIC model detection criteria, the student’s t-test was employed to calculate the *p*-values for the association abilities under the null hypothesis $H_{0}:\alpha_{ij}=0$to determine the significant protein associations. The *p*-values computed were then adjusted by Bonferroni correction to avoid a lot of spurious positives. The associations which adjusted *p*-value ≤ 0.05 were determined as significant associations and were preserved in the protein association network.

Briefly, we use the AIC method to obtain how many system orders, which mean the numbers of interactions, in the dynamic system of the association abilities (model). We use the above maximum likelihood estimate method to identify the parameter $\hat{\alpha}_{ij}$ and then employ AIC and student t-test to calculate *p-*values of association abilities for determining the significant PPIs for the target protein *i* by pruning the insignificant PPIs.
