# Supplementary material for: Multiple target drug cocktail design for attacking the core network markers of four cancers using ligand-based and structure-based virtual screening methods
Source: BMC Med Genomics. 2015 Dec 9;8(Suppl 4):S4. doi: 10.1186/1755-8794-8-S4-S4 (PMC4682379; doi:10.1186/1755-8794-8-S4-S4)
Supplement: Additional file 3 — The identified significant proteins in 4 cancers. [file 1755-8794-8-S4-S4-S3.docx]

**Additional File 3**

**Table S1 - The identified significant proteins in bladder, colorectal, liver, and lung carcinogenesis.**

* The common proteins of four types of cancer.

# The distinctive proteins from other three cancers.

| **Bladder cancer (N=107)** | | |  | **Liver cancer (N=110)** | | |
| --- | --- | --- | --- | --- | --- | --- |
| **CRV** | **Name** | ***p-*value** |  | **CRV** | **name** | ***p*-value** |
| 158.5321 | UBC* | < 1e-5 |  | 137.284 | UBC* | < 1e-5 |
| 19.5883 | TP53* | < 1e-5 |  | 19.5736 | HDAC1* | < 1e-5 |
| 19.2879 | HDAC1* | < 1e-5 |  | 18.7422 | TP53* | < 1e-5 |
| 16.7366 | CDK2* | < 1e-5 |  | 16.7663 | KIAA0101* | < 1e-5 |
| 15.8188 | KIAA0101* | < 1e-5 |  | 15.8533 | SUMO1 | < 1e-5 |
| 13.3217 | PCNA* | < 1e-5 |  | 15.1438 | PCNA* | < 1e-5 |
| 13.2669 | CUL1* | < 1e-5 |  | 14.0690 | CDK2* | < 1e-5 |
| 13.0423 | MYC* | < 1e-5 |  | 13.2218 | EP300* | < 1e-5 |
| 13.0117 | CUL3* | < 1e-5 |  | 12.9519 | CUL3* | < 1e-5 |
| 12.1078 | EP300* | < 1e-5 |  | 12.6421 | YWHAZ* | < 1e-5 |
| 12.0995 | CREBBP* | < 1e-5 |  | 11.2802 | CUL1* | < 1e-5 |
| 11.6611 | VHL | < 1e-5 |  | 10.8312 | CUL5 | < 1e-5 |
| 11.6233 | CUL2 | < 1e-5 |  | 10.7821 | MYC* | < 1e-5 |
| 11.5863 | BRCA1* | < 1e-5 |  | 10.6794 | AR | < 1e-5 |
| 11.0938 | HDAC2* | < 1e-5 |  | 10.3758 | ESR1* | < 1e-5 |
| 9.2003 | TRAF2* | < 1e-5 |  | 10.2716 | CUL2 | < 1e-5 |
| 8.8026 | SRC* | < 1e-5 |  | 9.7752 | HDAC2* | 0.00002 |
| 8.7995 | YWHAZ* | < 1e-5 |  | 9.5993 | CTNNB1* | 0.00002 |
| 8.6685 | ATM# | < 1e-5 |  | 9.5856 | CREBBP* | 0.00002 |
| 8.6624 | UCHL5 | < 1e-5 |  | 9.5585 | XRCC6* | 0.00002 |
| 8.5157 | RELA | < 1e-5 |  | 9.1997 | HDAC6 | 0.00004 |
| 7.9985 | CSNK2A1 | < 1e-5 |  | 8.7723 | RELA | 0.00007 |
| 7.6796 | CTNNB1* | < 1e-5 |  | 7.7978 | PSMA3* | 0.00010 |
| 7.6209 | SMARCA4 | < 1e-5 |  | 7.7449 | SMARCA4 | 0.00010 |
| 7.1345 | CUL5 | 0.00001 |  | 7.0852 | PARP1 | 0.00016 |
| 7.0345 | AKT1 | 0.00001 |  | 6.8526 | CUL4A | 0.00017 |
| 7.0177 | SMAD3 | 0.00001 |  | 6.8236 | BRCA1* | 0.00017 |
| 6.8922 | RB1* | 0.00002 |  | 6.7171 | PIK3R1 | 0.00019 |
| 6.7340 | IKBKG | 0.00003 |  | 6.6842 | PSMA2 | 0.00022 |
| 6.6287 | RAD21 | 0.00004 |  | 6.6460 | IRAK1* | 0.00023 |
| **Bladder cancer (cont.)** | | |  | **Liver cancer (cont.)** | | |
| **CRV** | **Name** | ***p*-value** |  | **CRV** | **Name** | ***p-*value** |
| 6.5794 | SIRT1 | 0.00004 |  | 6.4883 | TRIM28# | 0.00027 |
| 6.5444 | RAD23A | 0.00004 |  | 6.4146 | PPP2CA | 0.00031 |
| 6.4468 | RPA1 | 0.00005 |  | 6.3572 | UCHL5 | 0.00031 |
| 6.2665 | CAV1 | 0.00009 |  | 6.2256 | FYN | 0.00033 |
| 6.1783 | PML | 0.00010 |  | 6.0239 | ISG15* | 0.00038 |
| 5.9832 | PIN1# | 0.00014 |  | 6.0180 | PTK2 | 0.00038 |
| 5.8659 | HDAC4* | 0.00016 |  | 5.9417 | EGFR | 0.00041 |
| 5.8136 | PSMD10 | 0.00017 |  | 5.9391 | MAPK1 | 0.00041 |
| 5.8022 | PSMA3* | 0.00018 |  | 5.9369 | PRKDC* | 0.00041 |
| 5.7800 | PARP1 | 0.00020 |  | 5.9326 | IKBKG | 0.00042 |
| 5.7285 | TRAF6 | 0.00022 |  | 5.8956 | SMAD2 | 0.00044 |
| 5.6189 | ESR1* | 0.00026 |  | 5.8397 | HDAC4* | 0.00048 |
| 5.5193 | CDKN1A | 0.00035 |  | 5.8021 | SOCS3 | 0.00048 |
| 5.5065 | MAPK1 | 0.00036 |  | 5.7348 | PSMC2 | 0.00053 |
| 5.4992 | PTPN11 | 0.00037 |  | 5.6979 | VHL | 0.00055 |
| 5.4808 | PTK2 | 0.00038 |  | 5.6600 | POLR2A | 0.00057 |
| 5.4642 | TERF1* | 0.00038 |  | 5.5696 | BUB1B | 0.00064 |
| 5.4465 | FYN | 0.00038 |  | 5.5623 | NFKB1 | 0.00064 |
| 5.4175 | CBL | 0.00043 |  | 5.5531 | RB1* | 0.00065 |
| 5.3860 | MCM2 | 0.00049 |  | 5.5034 | ARRB2 | 0.00071 |
| 5.3247 | MAPK3 | 0.00061 |  | 5.4753 | CDKN1A | 0.00071 |
| 5.2871 | XRCC6* | 0.00063 |  | 5.4193 | RUVBL2 | 0.00075 |
| 5.2666 | HIF1A# | 0.00068 |  | 5.3650 | CDK9 | 0.00080 |
| 5.2443 | ZBTB16 | 0.00072 |  | 5.3402 | CDK4# | 0.00082 |
| 5.2303 | CEBPB* | 0.00075 |  | 5.2323 | RAD23A | 0.00099 |
| 5.1927 | EGFR | 0.00081 |  | 5.1866 | PSMA1 | 0.00104 |
| 5.1852 | MSH2# | 0.00081 |  | 5.1507 | CDC20 | 0.00109 |
| 5.1728 | E2F1 | 0.00085 |  | 5.1194 | CAV1 | 0.00115 |
| 5.1682 | NFKB1 | 0.00087 |  | 5.1193 | CCNB1 | 0.00115 |
| 5.1451 | RUVBL2 | 0.00094 |  | 5.0936 | YY1 | 0.00121 |
| 5.1106 | ITCH# | 0.00100 |  | 5.0894 | AKT1 | 0.00122 |
| 5.0836 | STUB1 | 0.00108 |  | 5.0858 | CUL4B | 0.00124 |
| 5.0181 | MGMT | 0.00124 |  | 5.0219 | TRAF6 | 0.00137 |
| 4.9555 | DDB1# | 0.00138 |  | 5.0135 | HDAC3 | 0.00137 |
| 4.9001 | SHC1 | 0.00157 |  | 4.9742 | RPA1 | 0.00143 |
|  |  |  |  |  |  |  |
| **Bladder cancer (cont.)** | | |  | **Liver cancer (cont.)** | | |
| **CRV** | **Name** | ***p*-value** |  | **CRV** | **Name** | ***p*-value** |
| 4.8586 | PTPN6# | 0.00164 |  | 4.9434 | RBBP4# | 0.00152 |
| 4.8562 | HDAC6 | 0.00165 |  | 4.9046 | TERF1* | 0.00163 |
| 4.8206 | CDC27# | 0.00182 |  | 4.8937 | NR3C1 | 0.00166 |
| 4.8138 | CHAF1A# | 0.00182 |  | 4.8138 | PTPN11 | 0.00189 |
| 4.7516 | RARA# | 0.00211 |  | 4.7783 | PSMC3# | 0.00203 |
| 4.6807 | JUP | 0.00250 |  | 4.7703 | TRAF2* | 0.00207 |
| 4.6605 | CHUK# | 0.00263 |  | 4.7530 | MDM2* | 0.00213 |
| 4.6426 | NR4A1# | 0.00277 |  | 4.7299 | GNB2L1# | 0.00223 |
| 4.6161 | CASP8# | 0.00298 |  | 4.7228 | CDT1 | 0.00224 |
| 4.6009 | PSMB5 | 0.00308 |  | 4.7213 | RAF1 | 0.00224 |
| 4.5775 | MCM7 | 0.00321 |  | 4.6929 | HGS# | 0.00232 |
| 4.5727 | UBE2D1 | 0.00325 |  | 4.5666 | LYN | 0.00292 |
| 4.5647 | MDM2* | 0.00331 |  | 4.5359 | PSMD6# | 0.00304 |
| 4.5400 | GSK3B | 0.00347 |  | 4.5338 | PSMA6# | 0.00304 |
| 4.5387 | CDC37# | 0.00347 |  | 4.5098 | STUB1 | 0.00324 |
| 4.5346 | PIAS4# | 0.00348 |  | 4.4813 | RBX1# | 0.00345 |
| 4.5274 | UBE2D3# | 0.00353 |  | 4.4330 | CEBPB* | 0.00390 |
| 4.5167 | CDK9 | 0.00361 |  | 4.4270 | SNCA | 0.00394 |
| 4.5072 | RAF1 | 0.00363 |  | 4.4188 | MGMT | 0.00395 |
| 4.4953 | RPA2# | 0.00381 |  | 4.4114 | CCNA2 | 0.00398 |
| 4.4856 | ISG15* | 0.00389 |  | 4.3524 | UBE2D1 | 0.00443 |
| 4.4437 | ERBB2# | 0.00436 |  | 4.3404 | PSMD10 | 0.00453 |
| 4.4333 | BCL2 | 0.00444 |  | 4.3377 | CDKN2A# | 0.00453 |
| 4.4200 | IKBKB | 0.00460 |  | 4.3368 | BARD1# | 0.00454 |
| 4.4138 | SOCS1# | 0.00465 |  | 4.2604 | CCND1 | 0.00514 |
| 4.3966 | SMAD4# | 0.00479 |  | 4.2135 | MAPK14# | 0.00566 |
| 4.3747 | ITSN1# | 0.00500 |  | 4.2124 | TOP2A# | 0.00567 |
| 4.3516 | POLR2A | 0.00519 |  | 4.2020 | JAK1# | 0.00579 |
| 4.3468 | CCND1 | 0.00526 |  | 4.1882 | JUP | 0.00595 |
| 4.3101 | CSNK2B | 0.00571 |  | 4.1824 | HSPB1# | 0.00602 |
| 4.2893 | PSMD2# | 0.00606 |  | 4.1402 | NFKBIA# | 0.00646 |
| 4.2145 | JUN | 0.00710 |  | 4.1298 | PRKCD# | 0.00656 |
| 4.1837 | AR | 0.00755 |  | 4.1223 | NCOA3# | 0.00665 |
| 4.1501 | TRAF1# | 0.00801 |  | 4.1125 | TERF2IP# | 0.00678 |
| 4.1157 | IRAK1* | 0.00861 |  | 4.1122 | PSMA4# | 0.00678 |
|  |  |  |  |  |  |  |
| **Bladder cancer (cont.)** | | |  | **Liver cancer (cont.)** | | |
| **CRV** | **Name** | ***p*-value** |  | **CRV** | **Name** | ***p*-value** |
| 4.0798 | SOCS3 | 0.00912 |  | 4.0767 | SRC* | 0.00737 |
| 4.0781 | PRKDC* | 0.00912 |  | 4.0556 | RFC4# | 0.00764 |
| 4.0759 | TRAF3# | 0.00914 |  | 4.0447 | MAD2L1 | 0.00781 |
| 4.0575 | CDKN1B# | 0.00950 |  | 4.0104 | PSMC5# | 0.00835 |
| 4.0569 | TOPBP1# | 0.00951 |  | 4.0023 | SET# | 0.00855 |
| 4.0514 | PIK3R1 | 0.00965 |  | 3.9986 | JUN | 0.00858 |
| 4.0396 | YY1 | 0.00991 |  | 3.9947 | E2F1 | 0.00862 |
|  | | |  | 3.9564 | CSNK2B | 0.00930 |
|  |  |  |  | 3.9551 | SMARCA2 | 0.00932 |
|  |  |  |  | 3.9283 | CEBPA# | 0.00983 |
