# Supplementary material for: Multiple target drug cocktail design for attacking the core network markers of four cancers using ligand-based and structure-based virtual screening methods
Source: BMC Med Genomics. 2015 Dec 9;8(Suppl 4):S4. doi: 10.1186/1755-8794-8-S4-S4 (PMC4682379; doi:10.1186/1755-8794-8-S4-S4)
Supplement: Additional file 4 — Docking results for the top 20 ligands for the 28 proteins studied. [file 1755-8794-8-S4-S4-S4.docx]

## Additional File 4

## Table S2- Docking results for the top 20 ligands for the 28 proteins studied

| **BRCA1(4IFI)** | | **CDK2(3QQK)** | | **CEBPB(2E42)** | | **CREBBP(4A9K)** | | **CTNNB1(3XT7)** | | **CUL1(4F52)** | | **CUL3(4EOZ)** | |
| --- | --- | --- | --- | --- | --- | --- | --- | --- | --- | --- | --- | --- | --- |
| NCI  Drug | LibDock Score | NCI  Drug | LibDock Score | NCI  Drug | LibDock Score | NCI  Drug | LibDock Score | NCI  Drug | LibDock Score | NCI  Drug | LibDock Score | NCI  Drug | LibDock Score |
| 668448 | 192.069 | 680359 | 108.336 | 625869 | 194.142 | 49714 | 88.6945 | 669230 | 96.5368 | 682932 | 139.644 | 702125 | 101.882 |
| 726771 | 142.956 | 678636 | 101.875 | 690021 | 171.236 | 668373 | 80.3823 | 665675 | 85.824 | 666346 | 135.442 | 675823 | 87.4137 |
| 639795 | 127.388 | 669299 | 101.27 | 690246 | 168.339 | 749 | 79.471 | 295632 | 81.2577 | 616358 | 130.947 | 689447 | 76.6491 |
| 697231 | 121.815 | 679065 | 101.006 | 724305 | 167.724 | 675278 | 78.1312 | 690569 | 80.9687 | 713370 | 130.826 | 680304 | 73.6548 |
| 629023 | 121.094 | 376791 | 100.92 | 722651 | 167.122 | 674553 | 77.1663 | 516286 | 80.6653 | 682363 | 130.025 | 673517 | 72.195 |
| 617007 | 120.471 | 667673 | 98.2425 | 700431 | 166.671 | 680827 | 77.1398 | 162907 | 80.5391 | 736612 | 124.15 | 697400 | 69.8673 |
| 661197 | 118.59 | 687005 | 97.3677 | 719325 | 165.99 | 690575 | 75.2064 | 639398 | 74.4584 | 716163 | 120.722 | 302988 | 67.4229 |
| 670653 | 116.92 | 370589 | 96.338 | 668604 | 164.562 | 619158 | 75.053 | 623238 | 73.9459 | 722175 | 119.845 | 684059 | 66.8509 |
| 677390 | 116.56 | 668394 | 95.6356 | 651241 | 164.164 | 22709 | 74.878 | 668375 | 73.8292 | 717217 | 115.27 | 669691 | 65.0017 |
| 661832 | 116.022 | 57975 | 95.3726 | 719276 | 162.46 | 686663 | 73.5679 | 666127 | 73.3719 | 35489 | 114.484 | 31664 | 64.6853 |
| 726260 | 115.746 | 667671 | 94.8508 | 669663 | 160.592 | 632947 | 73.25 | 125214 | 73.2725 | 663289 | 114.351 | 9908 | 64.2291 |
| 633643 | 115.339 | 664145 | 94.0772 | 697189 | 160.288 | 715694 | 72.8666 | 684753 | 72.855 | 679514 | 113.932 | 665663 | 63.8744 |
| 656722 | 114.913 | 668455 | 94.003 | 700443 | 160.107 | 1847 | 72.7543 | 3039 | 72.6418 | 630363 | 110.619 | 689741 | 62.0706 |
| 740001 | 114.421 | 685267 | 93.973 | 371682 | 158.801 | 625159 | 72.4409 | 329138 | 71.9422 | 701735 | 109.544 | 701992 | 62.0208 |
| 667476 | 113.576 | 361672 | 93.4335 | 748494 | 158.729 | 675585 | 71.6407 | 715452 | 71.898 | 617824 | 108.937 | 2023 | 61.8505 |
| 658140 | 112.825 | 687003 | 93.3742 | 645378 | 158.637 | 37049 | 70.9569 | 610187 | 71.2869 | 29638 | 108.223 | 677782 | 61.4764 |
| 709899 | 112.247 | 684968 | 92.7346 | 685968 | 158.452 | 694099 | 70.8263 | 656987 | 70.5151 | 689190 | 105.364 | 644582 | 61.4708 |
| 694154 | 111.933 | 676392 | 91.6976 | 697160 | 158.439 | 694100 | 70.8263 | 610186 | 70.4714 | 617970 | 104.381 | 620111 | 60.8005 |
| 717077 | 111.22 | 631964 | 91.2874 | 98535 | 158.036 | 1390 | 70.6411 | 717122 | 70.1788 | 623055 | 100.178 | 688119 | 60.586 |
| 629021 | 111.021 | 693324 | 91.1859 | 10010 | 158.025 | 693886 | 70.4497 | 698948 | 69.6857 | 652565 | 98.2237 | 740122 | 60.0541 |

| **EP300(4BHW)** | | **ESR1(1UOM)** | | **HDAC1(4BKX)** | | **HDAC2(4LY1)** | | **HDAC4(2VQM)** | | **IRAK4(2NRU)** | | **ISG15(3SDL)** | |
| --- | --- | --- | --- | --- | --- | --- | --- | --- | --- | --- | --- | --- | --- |
| NCI  Drug | LibDock Score | NCI  Drug | LibDock Score | NCI  Drug | LibDock Score | NCI  Drug | LibDock Score | NCI  Drug | LibDock Score | NCI  Drug | LibDock Score | NCI  Drug | LibDock Score |
| 153353 | 99.4724 | 657381 | 95.8424 | 627865 | 134.685 | 666854 | 109.551 | 684438 | 155.185 | 654626 | 153.82 | 76519 | 106.129 |
| 9345 | 98.5378 | 627399 | 94.3326 | 625439 | 134.097 | 697191 | 103.809 | 699408 | 154.589 | 697155 | 125.343 | 682086 | 101.495 |
| 146876 | 88.1996 | 693235 | 89.4985 | 647638 | 125.505 | 701745 | 103.664 | 704565 | 153.564 | 60673 | 119.343 | 641847 | 98.9475 |
| 17391 | 86.8089 | 632624 | 88.8011 | 2426 | 124.722 | 700011 | 98.3774 | 673354 | 152.438 | 693118 | 113.554 | 682087 | 96.5367 |
| 643467 | 83.1517 | 645318 | 88.2848 | 707841 | 117.076 | 696561 | 97.9729 | 691703 | 150.675 | 735798 | 111.147 | 102815 | 93.5534 |
| 74518 | 82.9346 | 702754 | 87.6234 | 633063 | 113.575 | 529861 | 96.2877 | 625439 | 149.324 | 677934 | 108.865 | 679185 | 86.1314 |
| 521778 | 81.2581 | 646922 | 87.3882 | 645389 | 113.539 | 698037 | 96.0747 | 668434 | 148.45 | 670053 | 107.441 | 698173 | 83.8733 |
| 626779 | 78.1005 | 691839 | 87.2011 | 265875 | 113.189 | 742553 | 93.3249 | 726771 | 148.366 | 726442 | 106.937 | 711014 | 79.2466 |
| 126766 | 75.3983 | 700991 | 87.0693 | 734996 | 112.454 | 706418 | 92.2939 | 699222 | 146.247 | 157036 | 104.909 | 698168 | 78.9329 |
| 163326 | 71.3625 | 715174 | 86.2038 | 637131 | 112.399 | 624664 | 90.8931 | 618571 | 144.187 | 37881 | 104.764 | 735034 | 78.4084 |
| 110344 | 59.4537 | 674102 | 86.196 | 740107 | 112.171 | 697236 | 90.4856 | 718726 | 142.109 | 735213 | 103.883 | 673343 | 77.9217 |
| 202537 | 56.542 | 677995 | 86.1253 | 645392 | 111.936 | 122870 | 90.2685 | 653419 | 141.798 | 676423 | 102.074 | 711012 | 77.6749 |
| 2654 | 55.7296 | 723380 | 86.0795 | 666063 | 111.783 | 721045 | 90.1792 | 724305 | 141.195 | 703068 | 101.711 | 742410 | 77.4437 |
| 609696 | 53.5665 | 13378 | 86.04 | 635565 | 107.588 | 670681 | 89.876 | 715563 | 140.295 | 708572 | 101.679 | 711010 | 76.74 |
| 622153 | 52.2578 | 637882 | 86.013 | 703134 | 99.4151 | 280594 | 88.9481 | 686479 | 139.759 | 669655 | 100.902 | 72861 | 76.7033 |
| 622154 | 51.6764 | 706028 | 85.7597 | 705953 | 99.2639 | 650907 | 88.9292 | 676817 | 138.369 | 703065 | 100.799 | 640674 | 75.7234 |
| 155080 | 50.6581 | 681075 | 85.5225 | 121129 | 98.973 | 696560 | 88.9042 | 681462 | 137.764 | 703063 | 100.726 | 632935 | 74.488 |
| 744479 | 41.0606 | 367238 | 85.2923 | 668021 | 98.8178 | 691823 | 88.8788 | 735846 | 137.432 | 703067 | 100.177 | 665775 | 74.0952 |
| 617069 | 31.7358 | 609965 | 84.7189 | 624177 | 93.0055 | 716908 | 88.8416 | 643589 | 136.642 | 698983 | 99.4155 | 711018 | 73.9747 |
| 716706 | 27.7423 | 710269 | 84.5874 | 652603 | 92.0072 | 659999 | 88.5542 | 743122 | 136.639 | 657377 | 99.247 | 711026 | 73.6138 |

| **KIAA0101** | | **MDM2(4MDN)** | | **MYC(1NKP)** | | **PCNA(3WGW)** | | **PRKDC(3KGV)** | | **PSMA3** | | **RB1(3POM)** | |
| --- | --- | --- | --- | --- | --- | --- | --- | --- | --- | --- | --- | --- | --- |
| NCI  Drug | LibDock Score | NCI  Drug | LibDock Score | NCI  Drug | LibDock Score | NCI  Drug | LibDock Score | NCI  Drug | LibDock Score | NCI  Drug | LibDock Score | NCI  Drug | LibDock Score |
| 655102 | 165.722 | 674086 | 136.098 | 698686 | 194.107 | 406433 | 122.435 | 684443 | 144.774 | 668577 | 180.11 | 686584 | 130.737 |
| 669588 | 164.036 | 76955 | 125.897 | 668433 | 181.273 | 683481 | 118.427 | 713288 | 137.911 | 717708 | 172.983 | 682292 | 121.514 |
| 407811 | 158.565 | 709896 | 118.466 | 737026 | 168.293 | 661908 | 116.71 | 710559 | 135.815 | 668448 | 166.516 | 154020 | 119.131 |
| 704564 | 148.676 | 688217 | 116.335 | 646614 | 155.611 | 626160 | 115.547 | 660224 | 129.374 | 695409 | 164.906 | 664154 | 118.972 |
| 698687 | 147.054 | 724910 | 116.15 | 350895 | 147.533 | 351521 | 113.94 | 639966 | 126.236 | 727367 | 161.91 | 657190 | 118.092 |
| 664983 | 146.996 | 62485 | 115.317 | 687363 | 145.138 | 685837 | 113.933 | 617309 | 124.846 | 653260 | 161.657 | 631392 | 115.868 |
| 625451 | 146.739 | 676436 | 112.718 | 742856 | 140.093 | 707572 | 112.971 | 725739 | 122.528 | 729648 | 160.321 | 93033 | 115.659 |
| 625439 | 145.708 | 671153 | 111.625 | 737025 | 135.854 | 61900 | 112.606 | 617613 | 122.316 | 671864 | 160.069 | 350378 | 115.175 |
| 623527 | 144.815 | 652623 | 106.658 | 722654 | 134.503 | 714364 | 111.807 | 602077 | 121.588 | 697156 | 158.812 | 675972 | 114.653 |
| 683897 | 142.757 | 716911 | 105.398 | 719660 | 134.021 | 706157 | 111.755 | 625885 | 121.371 | 661423 | 157.044 | 735341 | 114.247 |
| 713288 | 141.277 | 724562 | 105.197 | 668432 | 131.495 | 627732 | 111.442 | 668579 | 119.726 | 720458 | 156.296 | 700411 | 113.865 |
| 645378 | 140.103 | 720388 | 104.213 | 677662 | 129.5 | 692402 | 110.588 | 655900 | 119.679 | 65104 | 155.237 | 734791 | 113.536 |
| 697157 | 139.485 | 626315 | 104.085 | 653867 | 128.864 | 631391 | 110.404 | 726365 | 118.206 | 629733 | 153.839 | 692602 | 113.505 |
| 704565 | 137.38 | 658958 | 102.922 | 691531 | 126.615 | 169517 | 110.394 | 638037 | 117.443 | 683781 | 152.793 | 20534 | 113.482 |
| 668891 | 136.34 | 695619 | 102.816 | 668577 | 125.603 | 661902 | 110.244 | 639018 | 117.167 | 668873 | 149.832 | 691581 | 112.419 |
| 706419 | 136.002 | 59349 | 102.706 | 708447 | 125.33 | 653264 | 109.894 | 647426 | 116.735 | 239400 | 149.493 | 666125 | 112.341 |
| 674709 | 135.976 | 665904 | 100.189 | 613528 | 125.013 | 657989 | 109.79 | 56614 | 115.355 | 695175 | 148.83 | 308848 | 112.193 |
| 695800 | 135.257 | 740033 | 98.8751 | 676779 | 124.621 | 631307 | 109.123 | 650721 | 114.737 | 668871 | 147.284 | 183519 | 112.172 |
| 715546 | 133.378 | 741107 | 98.5621 | 627966 | 122.233 | 661909 | 108.855 | 722664 | 114.52 | 618486 | 146.717 | 627221 | 112.068 |
| 707826 | 133.333 | 645673 | 97.7939 | 633660 | 121.775 | 710117 | 108.484 | 747167 | 114.52 | 743438 | 146.207 | 340555 | 112.032 |

| **SRC(2SRC)** | | **TERF1(3BQO)** | | **TP53(1TSR)** | | **TRAF2(1D0A)** | | **UBC(4FJV)** | | **XRCC6(1JEQ)** | | **YWHAZ(4HKC)** | |
| --- | --- | --- | --- | --- | --- | --- | --- | --- | --- | --- | --- | --- | --- |
| NCI  Drug | LibDock Score | NCI  Drug | LibDock Score | NCI  Drug | LibDock Score | NCI  Drug | LibDock Score | NCI  Drug | LibDock Score | NCI  Drug | LibDock Score | NCI  Drug | LibDock Score |
| 718308 | 153.089 | 698233 | 185.379 | 673172 | 126.112 | 653000 | 127.243 | 719481 | 122.887 | 668437 | 304.621 | 698229 | 213.077 |
| 718013 | 143.95 | 740601 | 129.253 | 695409 | 125.821 | 686501 | 125.097 | 633409 | 114.187 | 668449 | 241.902 | 725986 | 207.038 |
| 717079 | 139.211 | 641236 | 126.044 | 682236 | 117.725 | 715469 | 113.53 | 672968 | 111.867 | 668447 | 225.628 | 719660 | 200.903 |
| 718165 | 138.305 | 682094 | 123.287 | 695405 | 115.63 | 692357 | 111.718 | 734999 | 111.578 | 668571 | 219.59 | 684034 | 195.223 |
| 694702 | 138.112 | 661210 | 121.902 | 667504 | 114.326 | 712006 | 111.448 | 688121 | 111.501 | 745943 | 212.869 | 705931 | 194.333 |
| 717101 | 136.958 | 740555 | 121.085 | 682235 | 107.121 | 652584 | 110.442 | 633410 | 111.241 | 737027 | 211.228 | 722308 | 193.581 |
| 699416 | 136.534 | 642077 | 119.008 | 698974 | 106.267 | 670859 | 107.906 | 715227 | 110.414 | 672428 | 209.12 | 623553 | 193.247 |
| 624953 | 134.051 | 686379 | 118.349 | 649748 | 105.649 | 652583 | 107.681 | 672967 | 110.106 | 676934 | 206.55 | 630197 | 192.757 |
| 681461 | 132.452 | 669392 | 118.299 | 725903 | 104.683 | 731648 | 104.995 | 688122 | 108.735 | 668436 | 204.28 | 697179 | 190.795 |
| 301477 | 131.729 | 735031 | 118.186 | 682576 | 102.005 | 745501 | 103.33 | 637660 | 108.614 | 682348 | 204.077 | 687369 | 189.447 |
| 665308 | 131.549 | 715583 | 115.337 | 649749 | 101.866 | 728316 | 102.949 | 639174 | 108.325 | 668450 | 203.961 | 619027 | 186.674 |
| 681322 | 131.406 | 715585 | 115.326 | 742856 | 100.787 | 676857 | 102.065 | 703028 | 106.902 | 746847 | 199.554 | 698230 | 185.973 |
| 633395 | 131.139 | 306231 | 114.928 | 726149 | 100.551 | 642924 | 101.552 | 703032 | 106.411 | 633663 | 199.306 | 695401 | 185.904 |
| 123127 | 131.125 | 677586 | 114.861 | 649551 | 100.453 | 695175 | 101.267 | 176323 | 106.386 | 707766 | 196.19 | 642601 | 184.834 |
| 704581 | 130.413 | 697230 | 114.805 | 721386 | 97.6704 | 732429 | 100.448 | 716853 | 105.48 | 684748 | 194.115 | 687363 | 184.649 |
| 682935 | 130.243 | 715588 | 114.639 | 742858 | 97.2967 | 276361 | 99.0878 | 1760 | 105.122 | 668438 | 191.729 | 625849 | 184.476 |
| 631687 | 129.232 | 715587 | 113.822 | 622170 | 96.775 | 730045 | 98.7211 | 732175 | 104.867 | 699190 | 189.461 | 618938 | 183.837 |
| 661213 | 128.908 | 655438 | 113.477 | 700214 | 96.6241 | 625349 | 98.4057 | 249910 | 104.712 | 698684 | 188.822 | 730631 | 183.827 |
| 672445 | 128.723 | 641223 | 112.788 | 734002 | 96.4564 | 668579 | 98.1835 | 13597 | 104.199 | 668439 | 187.449 | 680418 | 183.132 |
| 652617 | 128.669 | 677089 | 112.106 | 628445 | 96.045 | 743407 | 97.991 | 11930 | 104.031 | 651806 | 184.194 | 682094 | 182.534 |
