# Supplementary material for: Multiple target drug cocktail design for attacking the core network markers of four cancers using ligand-based and structure-based virtual screening methods
Source: BMC Med Genomics. 2015 Dec 9;8(Suppl 4):S4. doi: 10.1186/1755-8794-8-S4-S4 (PMC4682379; doi:10.1186/1755-8794-8-S4-S4)
Supplement: Additional file 5 — new 6: User interface of commercial software and free webserver. [file 1755-8794-8-S4-S4-S5.docx]

## Additional File 5

## S5 -User Interface of commercial software and free webserver

## S5.1:PANTHER user interface


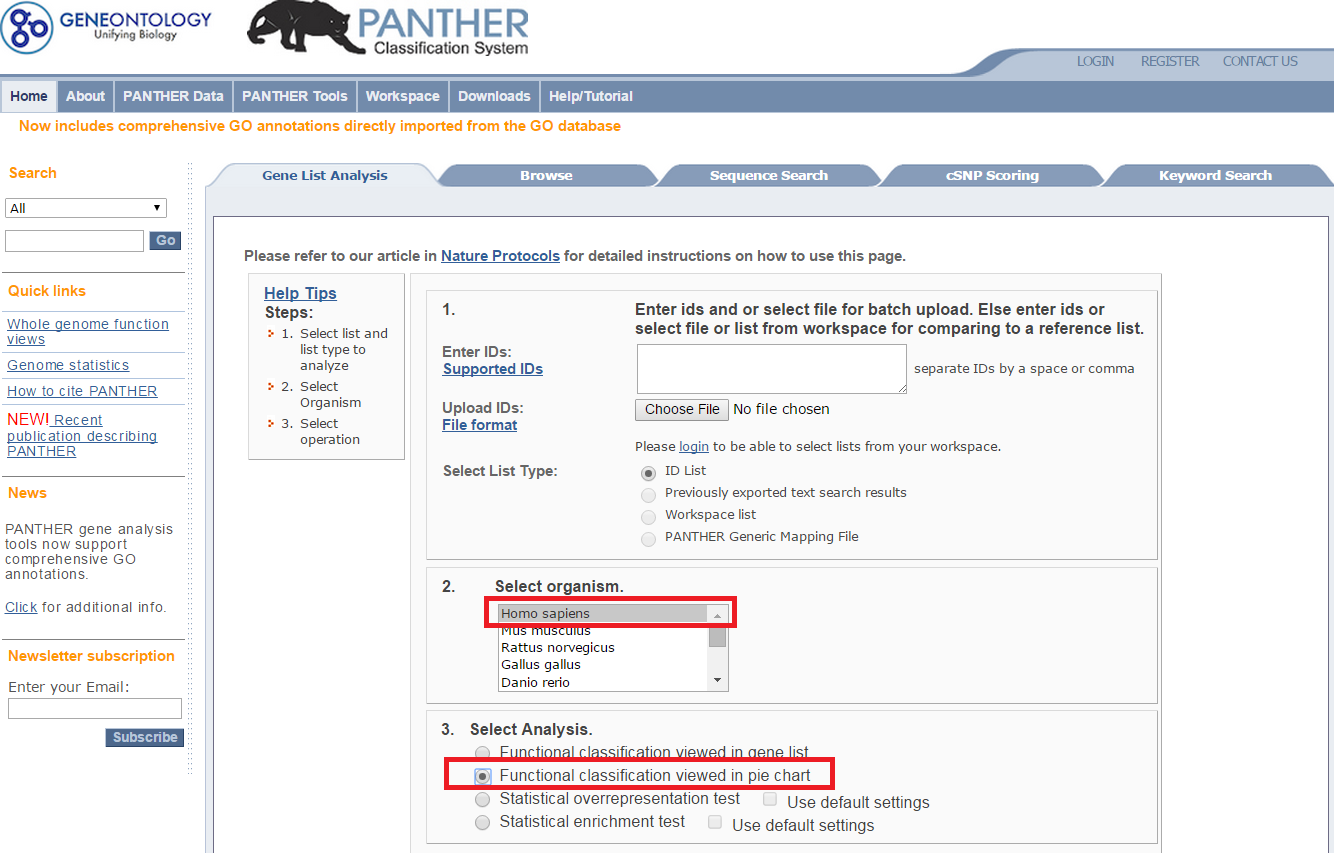


**Description:** We paste 28 genes on the first box. Set the organism parameter to be Homo sapiens. Use the pie chart to see the results.

## S5.2: I-TASSER user interface


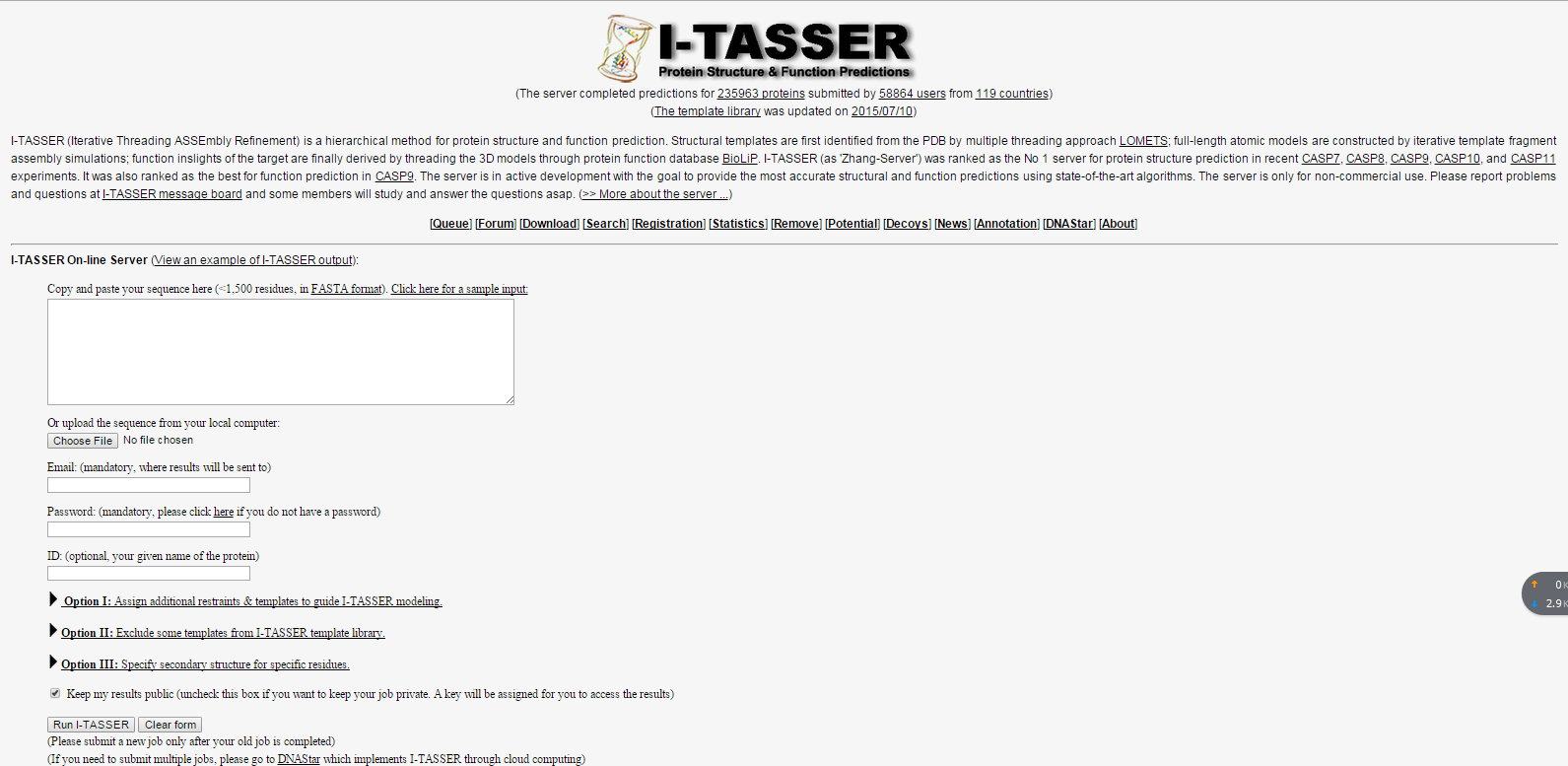


**Description:** We used the I-TASSER V4.2 which was released on 2014/10/27. We just copy and paste the protein sequence in the box. There are three options, and we do not use these options at this stage.

## S5.3: COACH user interface


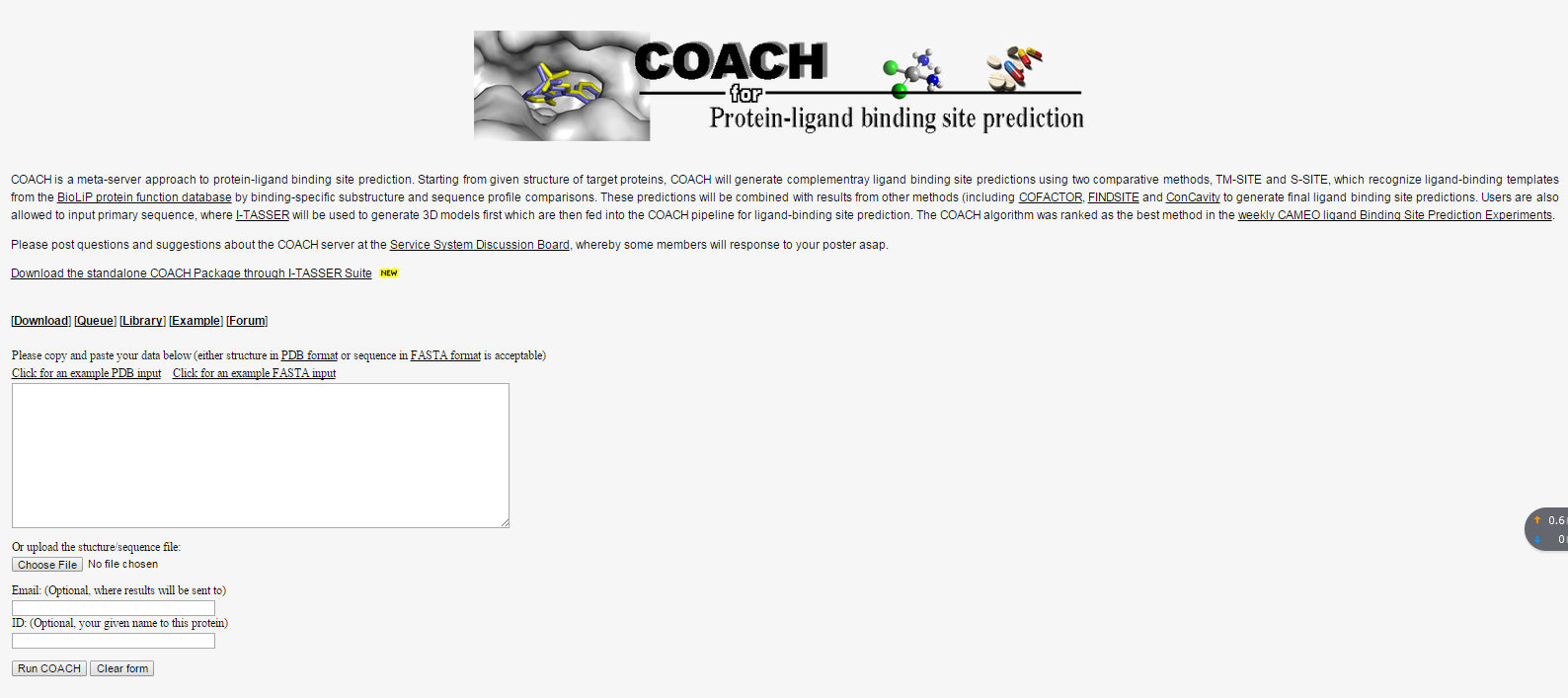


**Description:** There is no version information about COACH. There is no parameter setting for COACH, too. So we just copy and paste the protein sequence in the box and then run COACH.

## S5.4: LibDock module of DS 3.5 parameter setting


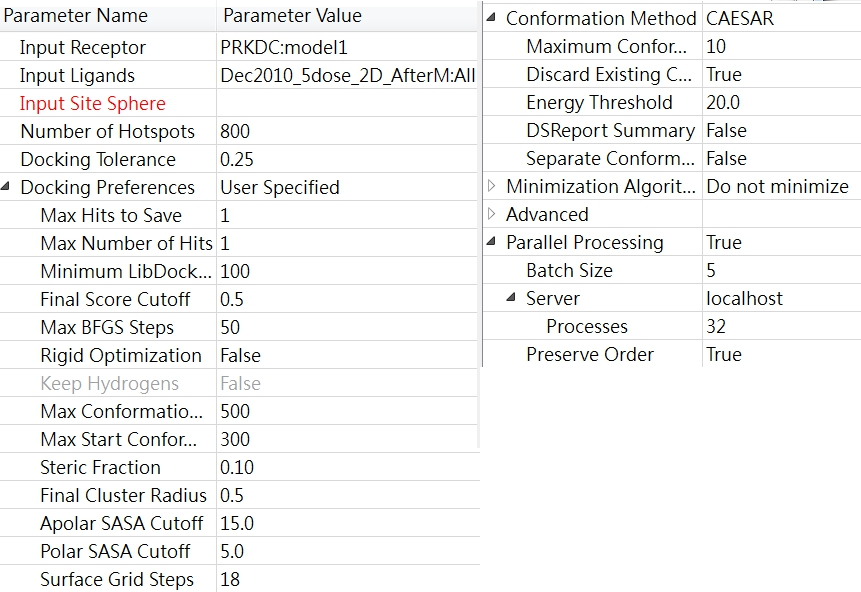


**Description:** Above is the detailed parameter setting of DS 3.5 Libdock module in the case of protein PRKDC and NCI anti-cancer drugs. Parameters shown in this table are the real parameters used in our docking experiments, and they are not the default parameters.

## S5.5: HYPOGEN (Pharmacophore) module of DS 3.5 parameter setting


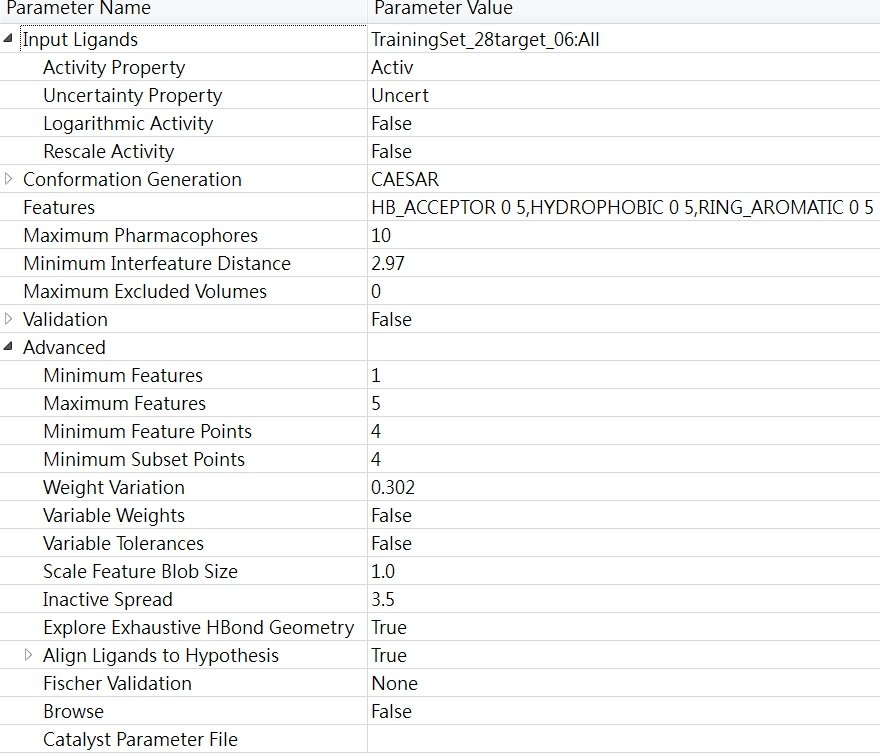


**Description:** Above is the detailed parameter setting of DS 3.5 HYPOGEN module in the case of our sixth training set. Parameters shown in this table are the real parameters used in our pharmacophore building experiments, and they are not the default parameters.

## S5.6: PharmGist user interface


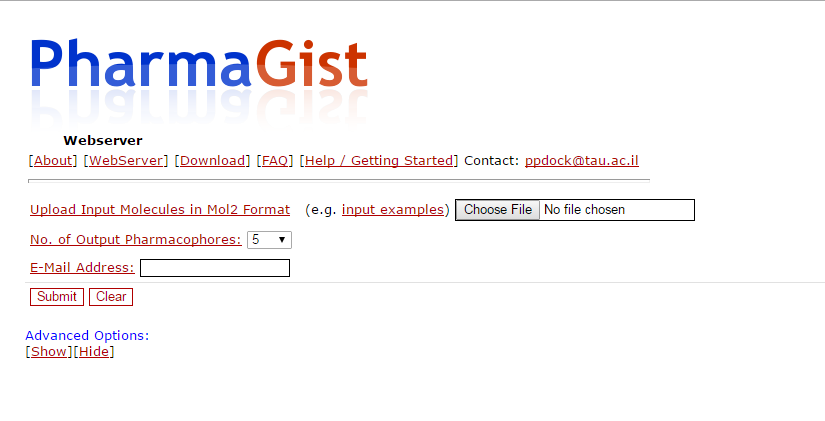


**Description:** We use the default parameter to build the pharmacophore model by PharmGist
