# Supplementary material for: Multiple target drug cocktail design for attacking the core network markers of four cancers using ligand-based and structure-based virtual screening methods
Source: BMC Med Genomics. 2015 Dec 9;8(Suppl 4):S4. doi: 10.1186/1755-8794-8-S4-S4 (PMC4682379; doi:10.1186/1755-8794-8-S4-S4)
Supplement: Additional file 6 — new 7: Mathematical foundation of Metacore. [file 1755-8794-8-S4-S4-S6.docx]

## Additional File 6

## Mathematical foundation of Metacore

1. Shortest paths:

Builds a network consisting of shortest paths between pairs of initial objects in each direction, using standard Dijkstra’s shortest paths algorithm. There are “from” and “to” lists of objects; by default the whole list of seed nodes is taken for each of them. Canonical pathways are considered by this algorithm as a single step and are used for network building only if the list of objects used for network building contains both "from" and "to" of a given pathway. The **Z-score, G-score and p-Value** are three different scoring functions used to rank the small networks created by the network building algorithm named “Analyze Network”. When viewing the most relevant networks list, you can sort the networks by a desired score, the actual size of the network, or the target size of the network by clicking on a column header.

1. Z-score:

Each subnetwork is associated with a Z-score which ranks the subnetworks according to saturation with the objects from the initial list of seed nodes. The Z-score ranks the subnetworks of the analyze network algorithm with regard to their saturation with genes from the experiment. A high Z-score means the network is highly saturated with genes from the experiment.

Z-score formula is given by

$\frac{\boldsymbol{r-n}\frac{\boldsymbol{R}}{\boldsymbol{N}}}{\sqrt{\boldsymbol{n}\left( \frac{\boldsymbol{R}}{\boldsymbol{N}} \right)\left( \boldsymbol{1-}\frac{\boldsymbol{R}}{\boldsymbol{N}} \right)\left( \boldsymbol{1-}\frac{\boldsymbol{n-1}}{\boldsymbol{N-1}} \right)}}$

**where**

N—total number of nodes in MetaCore™ database;

R—number of the network objects corresponding to the genes and proteins in your list;

n—total number of nodes in each small network generated from your list;

r—number of nodes with data in each small network generated from your list.

1. G-score

The G-score modifies the Z-score based on the number of Canonical Pathways used to build the network. If a network has a high G-score, it is saturated with expressed genes (from Z-score) and it contains many Canonical Pathways. Sorting the table by this value essentially enables you to sort the table by two factors at once.

1. p-Value

The p-Values throughout MetaCore™—for maps, networks and processes—are all calculated using the same basic formula for hypergeometric distribution. The p-Value essentially represents the probability for a particular mapping of an experiment to a map (or network, or process, etc.) to arise by chance, considering the numbers of genes in experiment versus the number of genes in the map (resp. network, process, etc.) within the “full set” of all genes on maps (resp. networks, processes, etc.).

***p*-Value formula**

$p-Value=\frac{R!n!\left( N-R \right)!\left( N-n \right)!}{N!}\sum_{i=max(r,R+n-N)}^{min(n,R)} \frac{1}{i!\left( R-i \right)!\left( n-i \right)!\left( N-R-n+i \right)!}$

where

N—total number of nodes in MetaCore™ database;

R—number of the network objects corresponding to the genes and proteins in your list;

n—total number of nodes in each small network generated from your list;

r—number of nodes with data in each small network generated from your list.
