# Supplementary material for: Multiple target drug cocktail design for attacking the core network markers of four cancers using ligand-based and structure-based virtual screening methods
Source: BMC Med Genomics. 2015 Dec 9;8(Suppl 4):S4. doi: 10.1186/1755-8794-8-S4-S4 (PMC4682379; doi:10.1186/1755-8794-8-S4-S4)
Supplement: Additional file 7 — new 5: Binding site information for the 28 proteins. [file 1755-8794-8-S4-S4-S7.docx]

## Additional File 7

## Binding site information for the 28 proteins

| **NO.** | **Protein** | **PDB**  **code** | **Site Sphere** | | | | **Predict** |
| --- | --- | --- | --- | --- | --- | --- | --- |
|  |  |  | **X** | **Y** | **Z** | **Radius (Å)** |  |
| **1** | BRCA1 | 4IFI | -15.5 | 17.3 | -23.6 | 13.2 | X |
| **2** | CDK2 | 3QQK | 66.6 | 152.6 | -48.3 | 8 | X |
| **3** | CEBPB | 2E42 | 37 | 34.8 | 43.3 | 15.6 | O |
| **4** | CREBBP | 4A9K | -18.3 | 27.5 | -0.1 | 8 | X |
| **5** | CTNNB1 | 3TX7 | -8.8 | -6.1 | -39.1 | 12.8 | X |
| **6** | CUL1 | 4F52 | 4.5 | -26.2 | 24.3 | 21.8 | O |
| **7** | CUL3 | 4EOZ | 108.5 | 45.5 | 44.6 | 13.2 | O |
| **8** | EP300 | 4BHW | -33.7 | -23.1 | 47.2 | 16.4 | X |
| **9** | ESR1 | 1UOM | -2.2 | 52.2 | 24.2 | 9.0 | X |
| **10** | HDAC1 | 4BKX | -45.0 | 17.2 | -5.9 | 13.6 | O |
| **11** | HDAC2 | 4LY1 | 22.2 | -18.4 | 0.67 | 9.7 | X |
| **12** | HDAC4 | 2VQM | 19.2 | -7.8 | -4.9 | 10.1 | X |
| **13** | IRAK4 | 2NRU | 29.2 | 7.36 | -4.79 | 11.5 | O |
| **14** | ISG15 | 3SDL | -4.9 | -38.0 | 109 | 16.1 | O |
| **15** | KIAA0101 | * | -1.4 | 14.4 | -10.4 | 10 | O |
| **16** | MDM2 | 4MDN | -17.7 | -4.78 | 1.8 | 12.4 | X |
| **17** | MYC | 1NKP | 74.1 | 86.2 | 32.5 | 20.6 | O |
| **18** | PCNA | 3WGW | -45 | -3.6 | 43.2 | 7.6 | X |
| **19** | PRKDC | 3KGV | 6.6 | 15.8 | -22.5 | 16.6 | O |
| **20** | PSMA3 | * | -3.4 | 7.1 | -2.9 | 12.8 | O |
| **21** | RB1 | 3POM | -24.1 | 3.2 | -19.6 | -19.6 | O |
| **22** | SRC | 2SRC | 17 | 20 | 58.8 | 8.1 | X |
| **23** | TERF1 | 3BQO | 47 | 124.1 | -19.1 | 17.5 | X |
| **24** | TP53 | 1TSR | 58 | 35 | 58.3 | 13.6 | O |
| **25** | TRAF2 | 1D0A | 24.3 | 38.5 | 63.1 | 10.7 | X |
| **26** | UBC | 4FJV | 5.5 | 9.2 | 1.4 | 13.7 | O |
| **27** | XRCC6 | 1JEQ | 17.4 | 11.5 | 117 | 23.3 | O |
| **28** | YWHAZ | 4HKC | 13.6 | 5.4 | 16.1 | 11.8 | X |

1. Binding site center and radius

X: The ligand is embedded. We use it as the binding site.

O: The binding site is predicted by COACH.

1. The 3D structure and corresponding key residues of binding site.

| 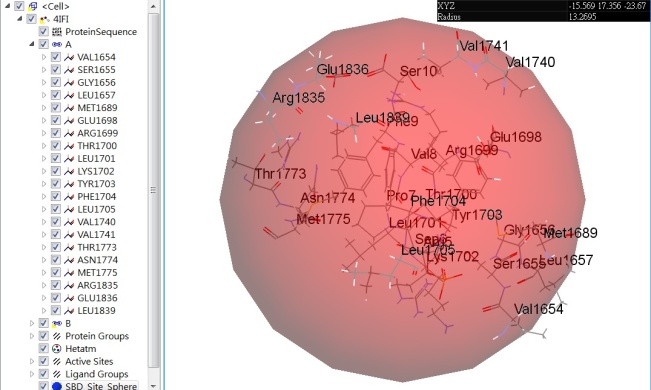 | 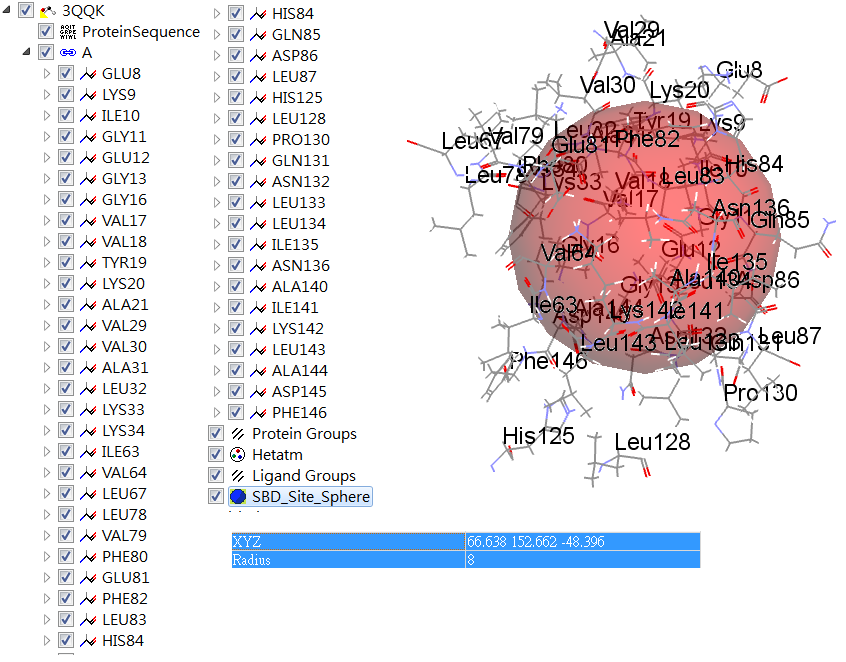 |
| --- | --- |
| 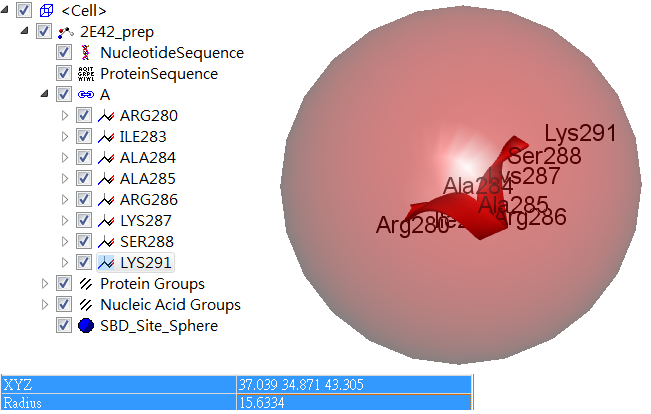 | 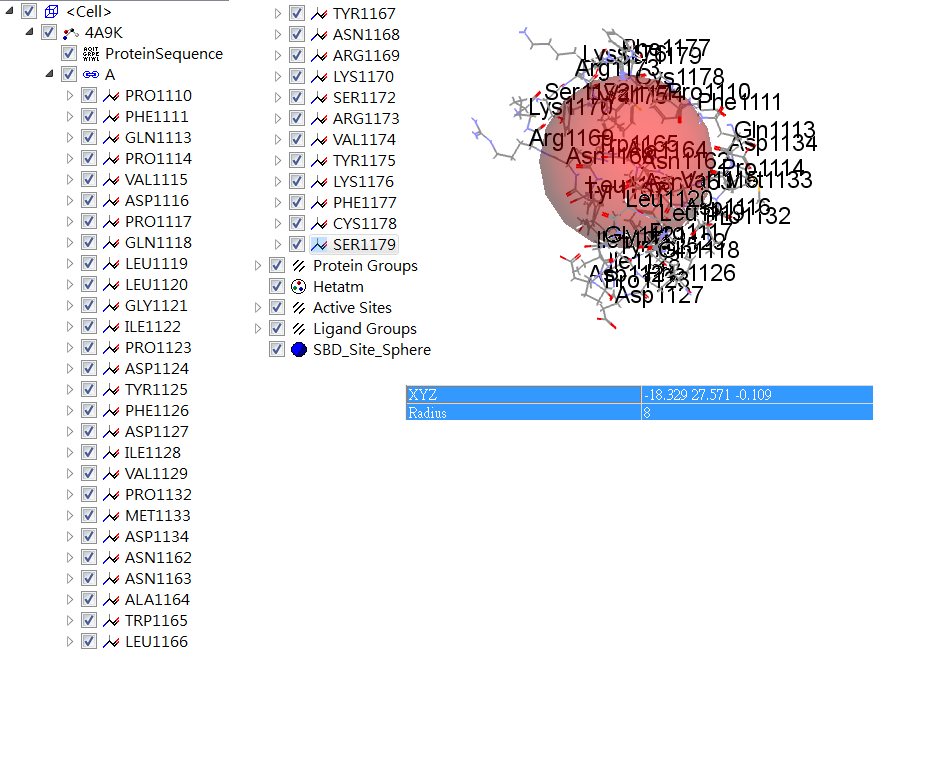 |
| 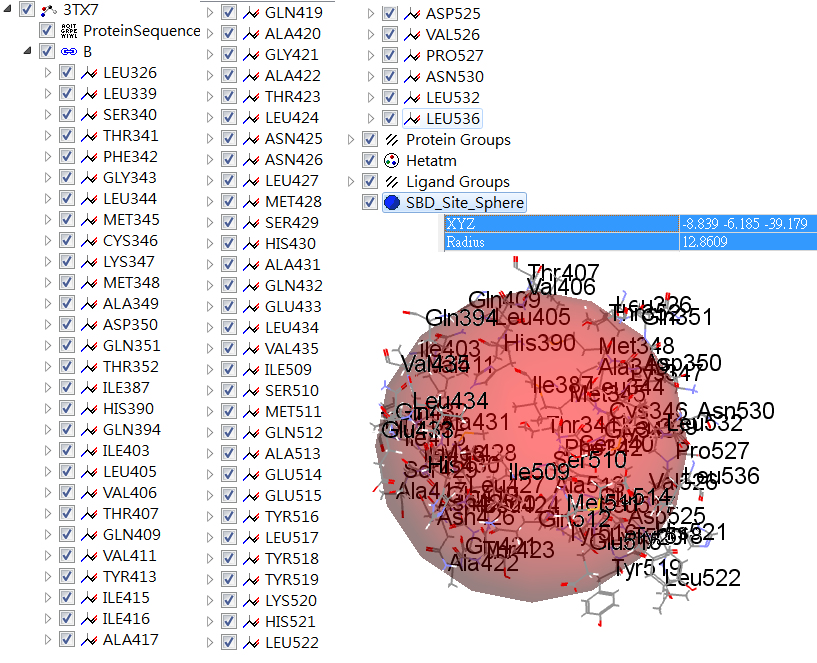 | 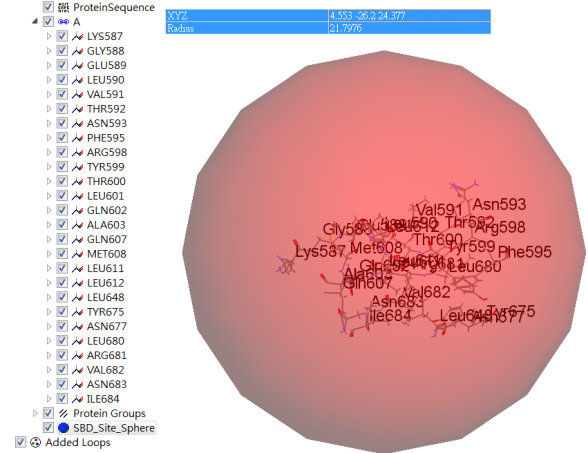 |
| 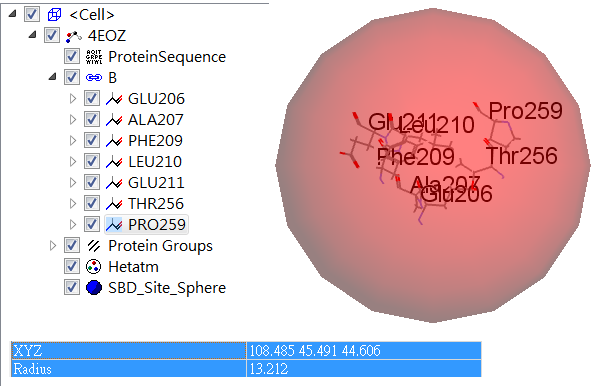 | 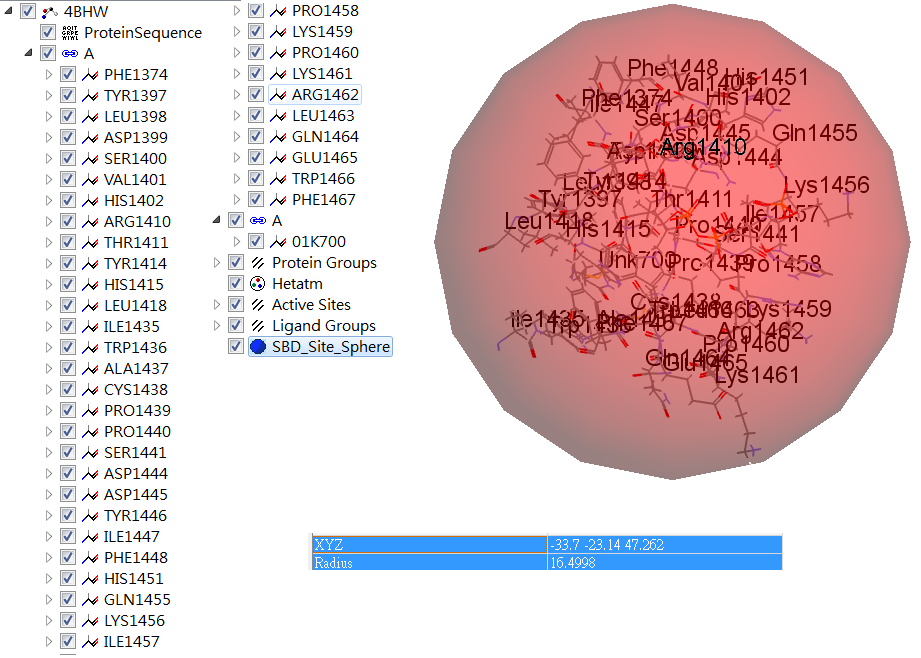 |

| 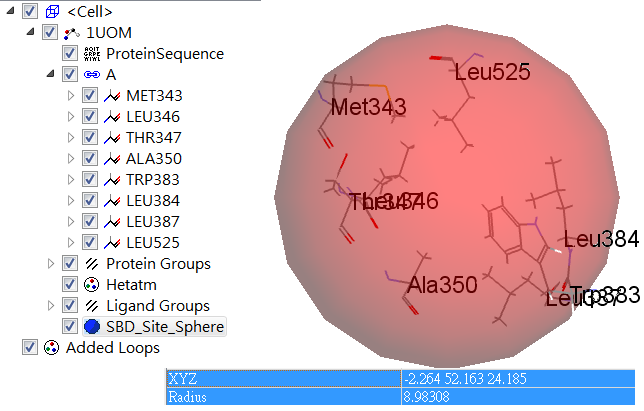 | 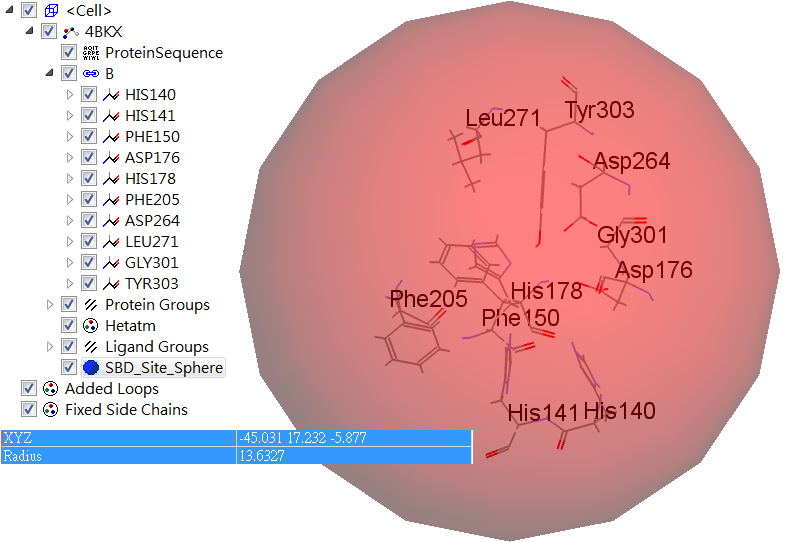 |
| --- | --- |
| 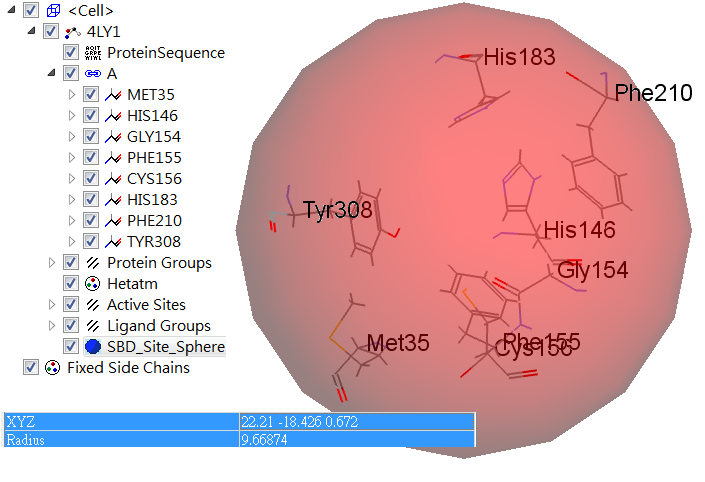 | 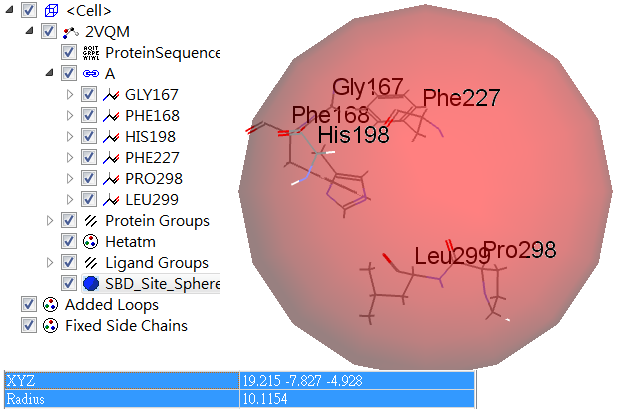 |
| 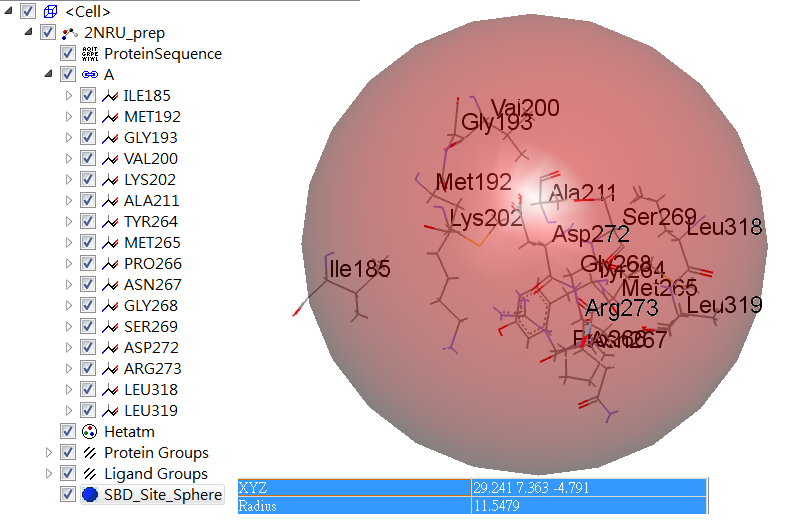 | 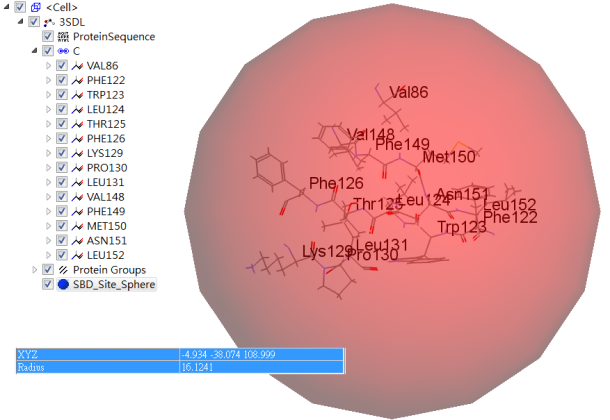 |
| 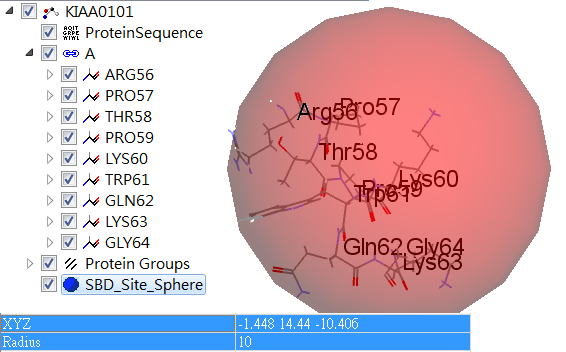 | 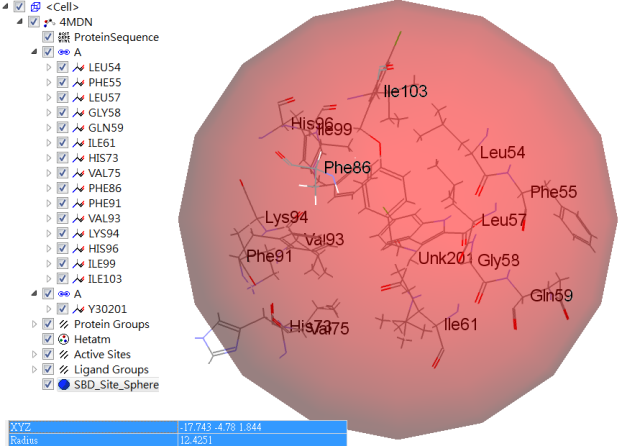 |

| 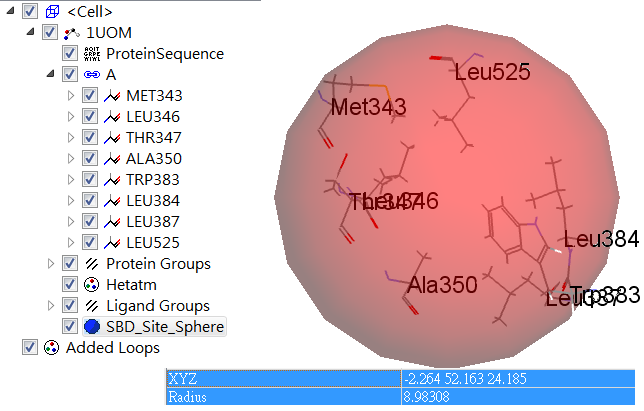 | 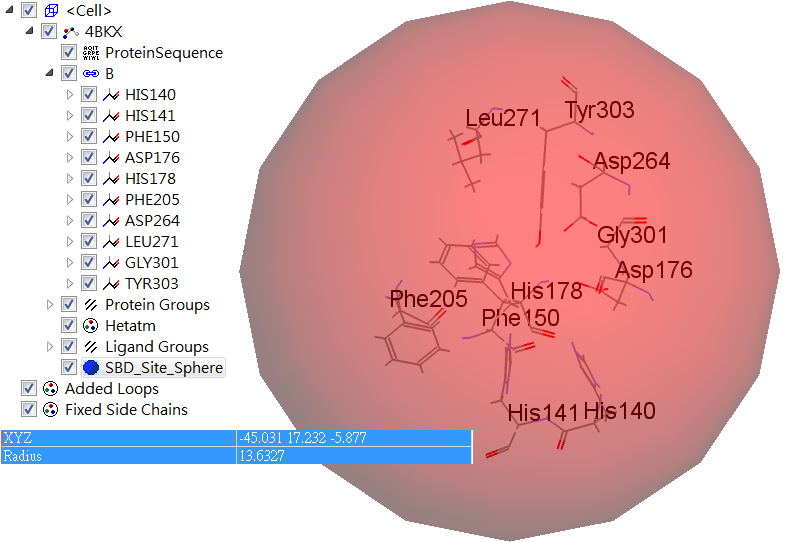 |
| --- | --- |
| 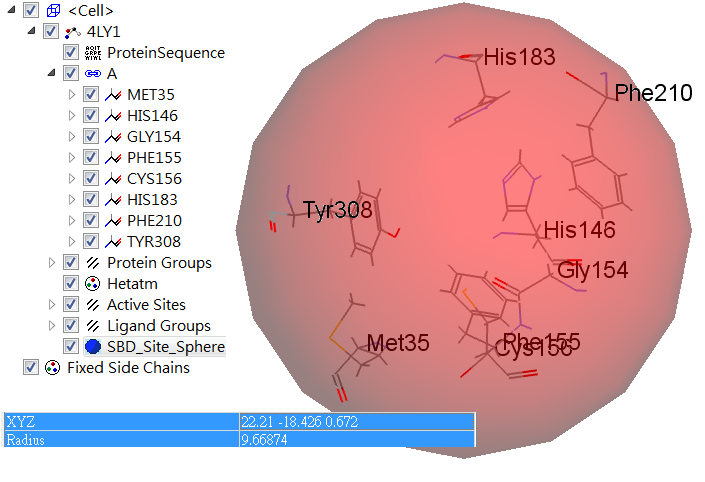 | 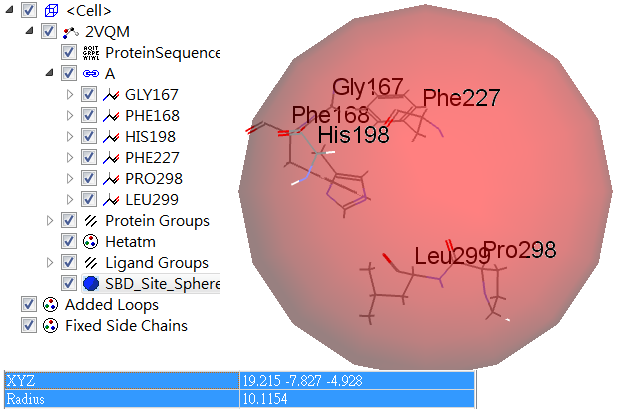 |
| 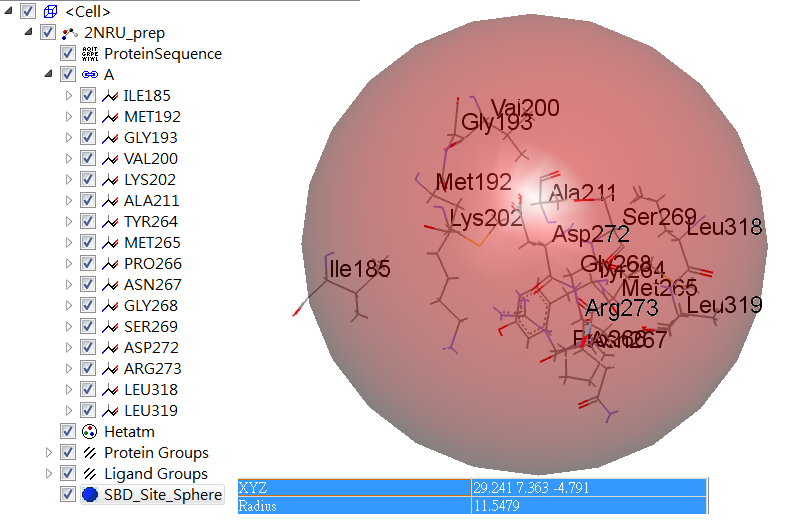 | 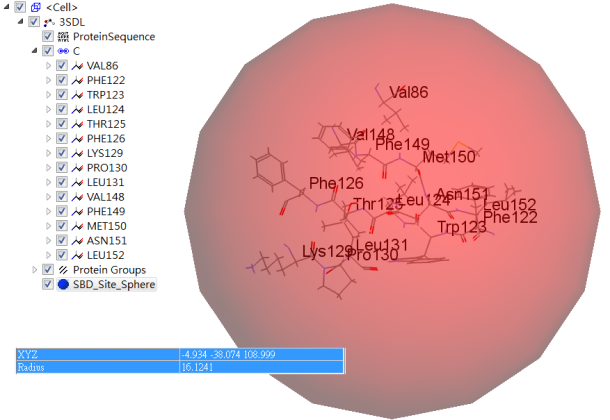 |
| 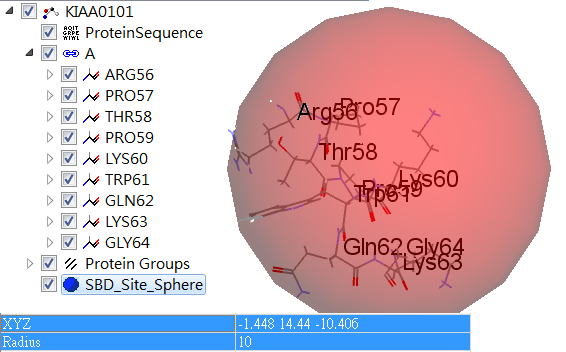 | 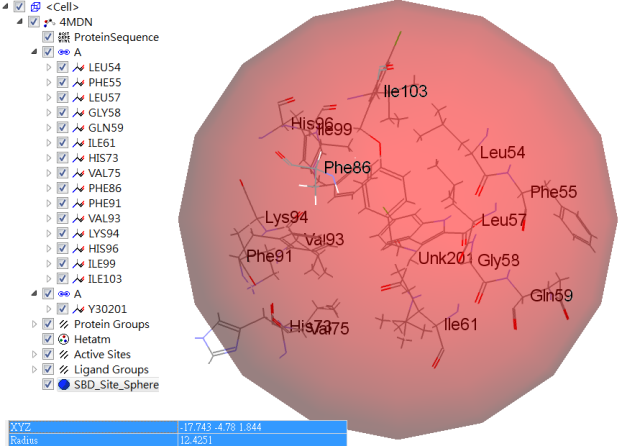 |

| 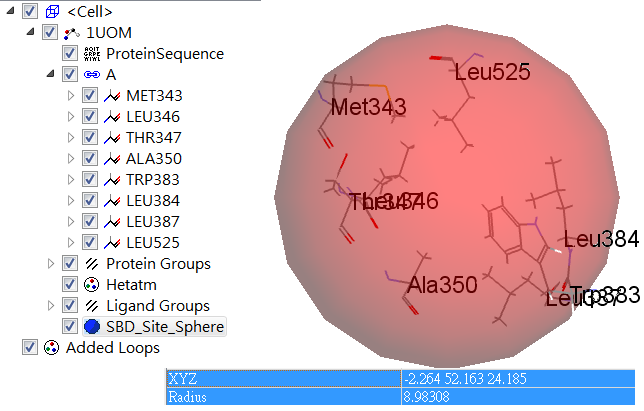 | 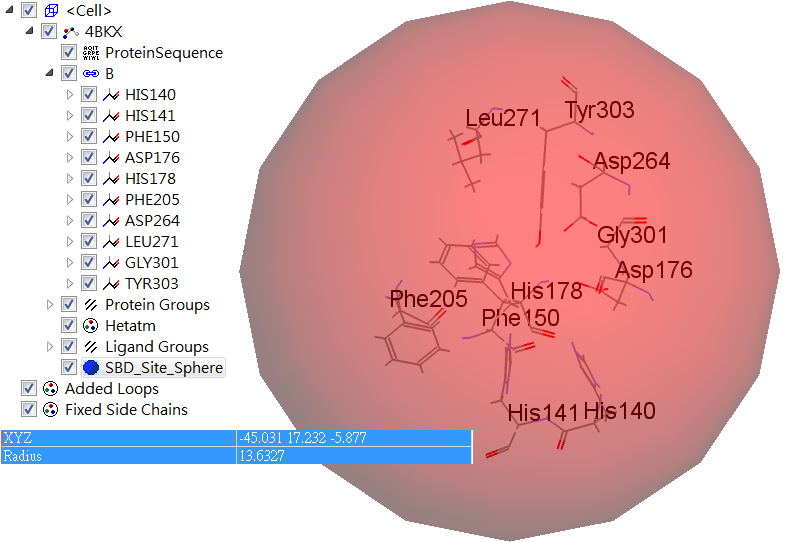 |
| --- | --- |
| 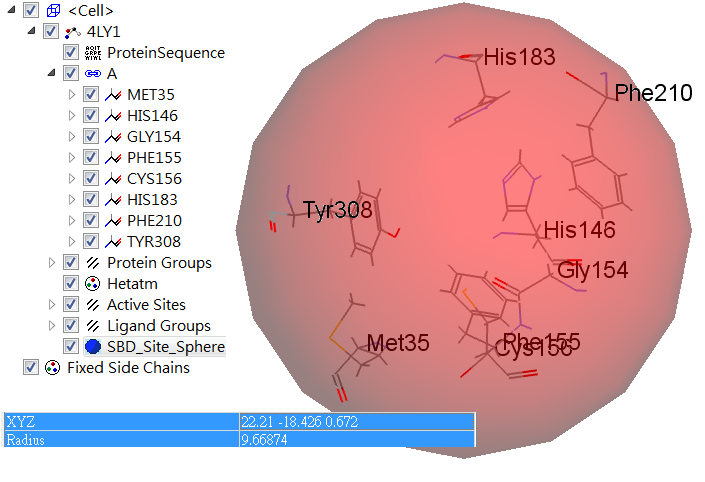 | 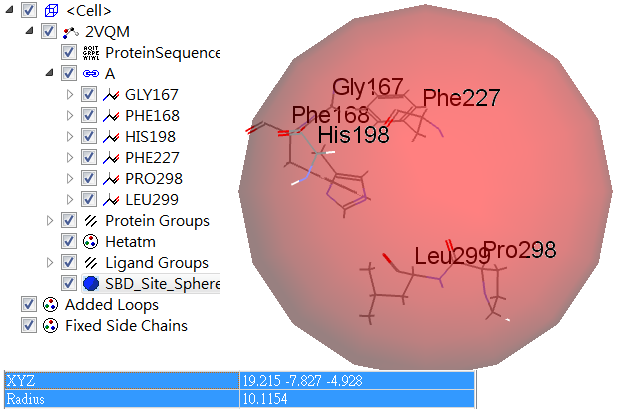 |
| 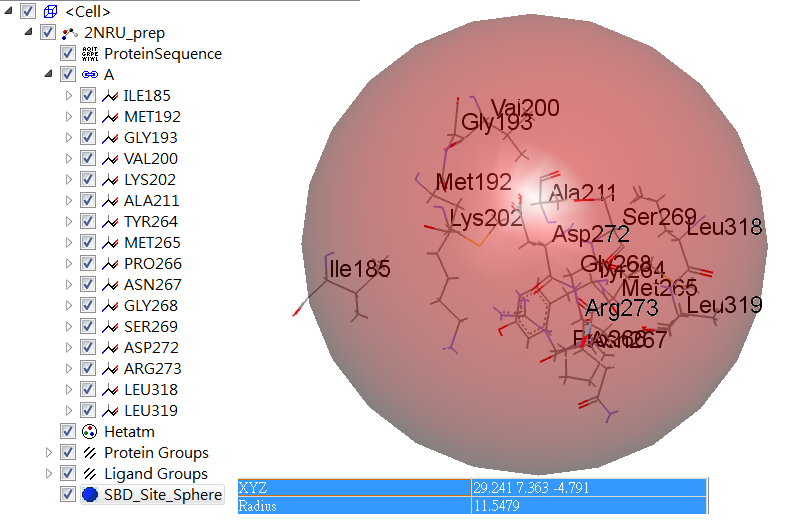 | 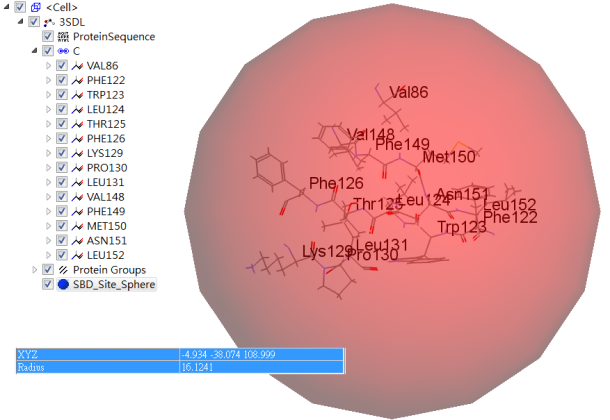 |
| 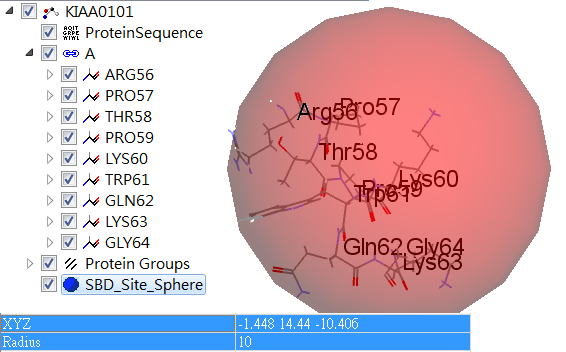 | 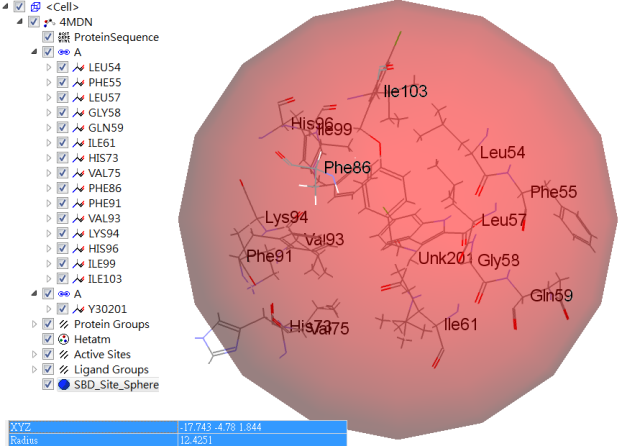 |
