# Supplementary material for: Multiple target drug cocktail design for attacking the core network markers of four cancers using ligand-based and structure-based virtual screening methods
Source: BMC Med Genomics. 2015 Dec 9;8(Suppl 4):S4. doi: 10.1186/1755-8794-8-S4-S4 (PMC4682379; doi:10.1186/1755-8794-8-S4-S4)
Supplement: Additional file 8 — new 10: Biological experimental validation. [file 1755-8794-8-S4-S4-S8.docx]

## Additional File 8

## Biological experimental validation

| 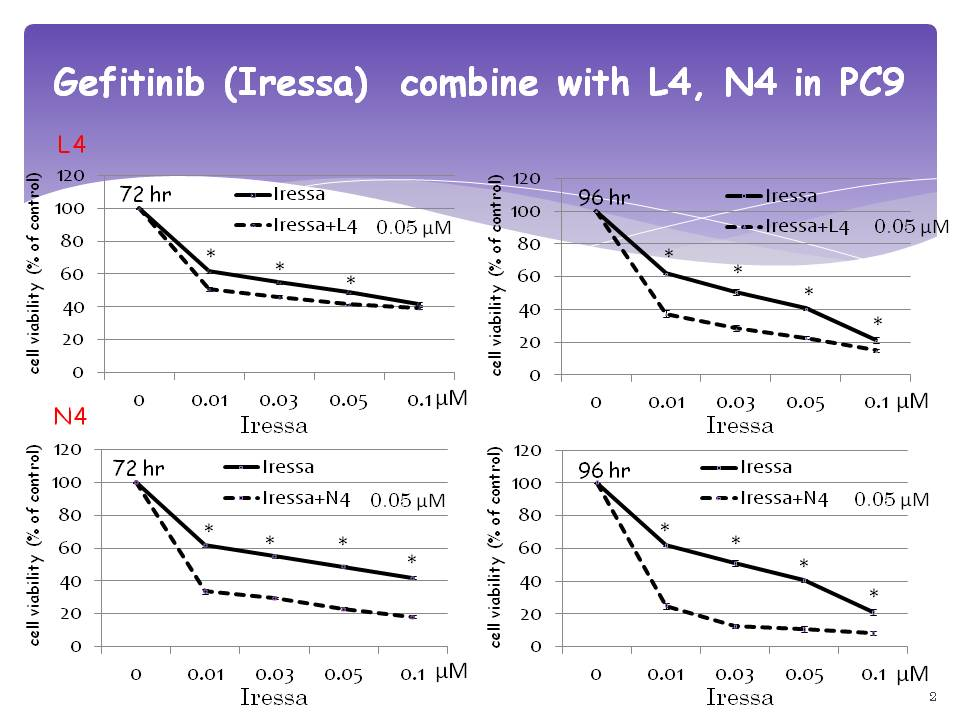 |
| --- |
| Figure S8(a): Gefitinib (Iressa) combine with L4 and N4 in PC9. |
| 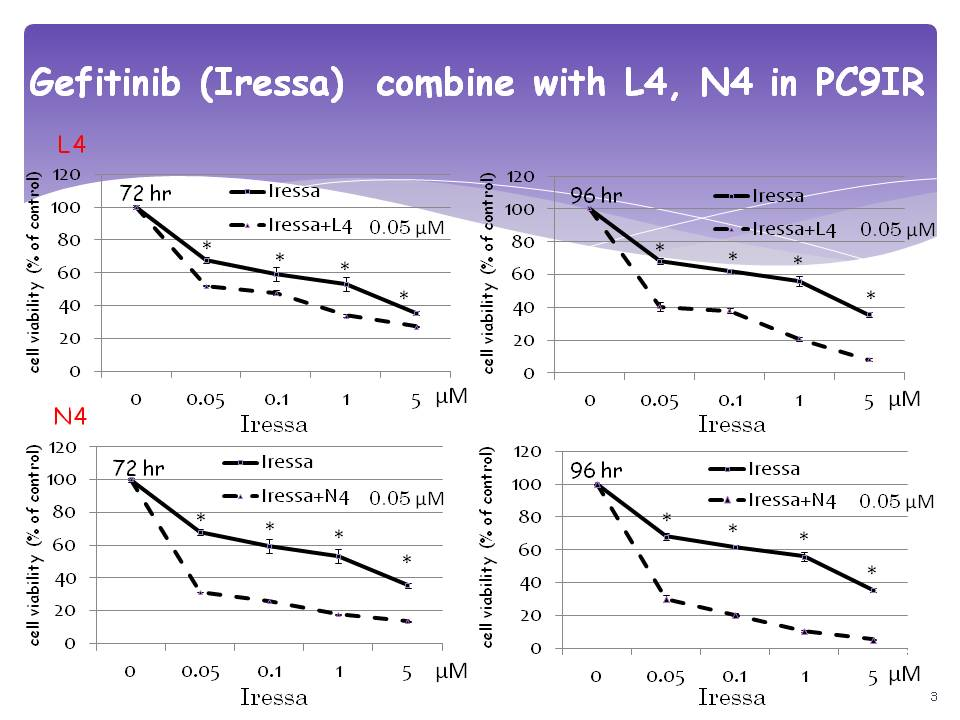 |
| Figure S8(b): Gefitinib (Iressa) combine with L4 and N4 in PC9IR. |
| 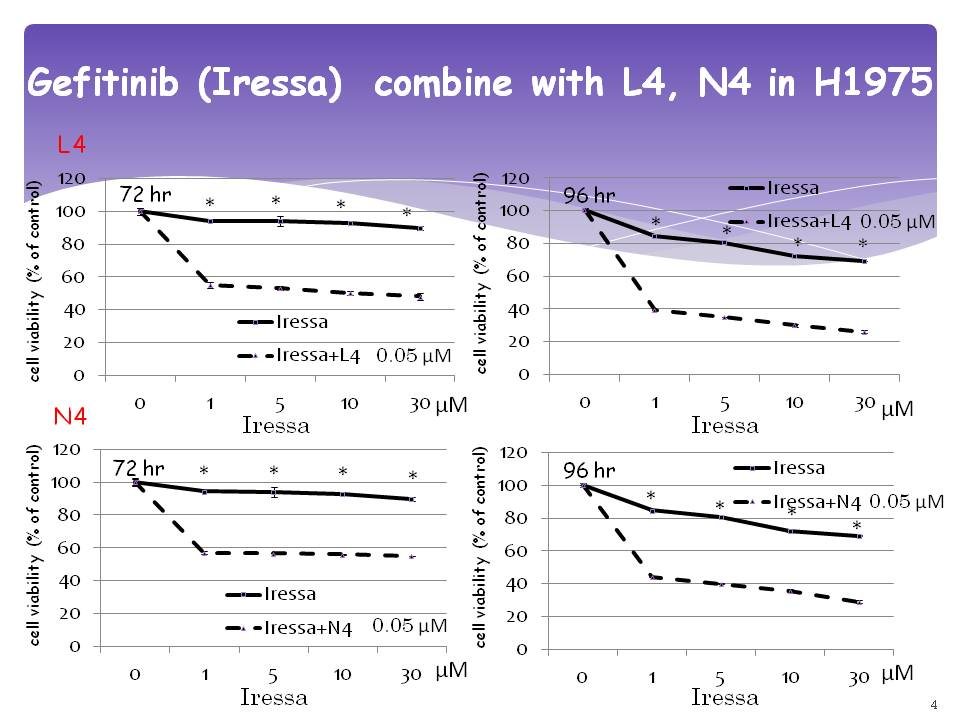 |
| Figure S8(c): Gefitinib (Iressa) combine with L4 and N4 in H1975. |

Figure S8: Drug combination- Gefitinib (Iressa) combine with L4 and N4 in three different cell lines. In the three cell lines, they show that combination drug always get a better efficiency than only single Iressa.
