# Supplementary material for: Multiple target drug cocktail design for attacking the core network markers of four cancers using ligand-based and structure-based virtual screening methods
Source: BMC Med Genomics. 2015 Dec 9;8(Suppl 4):S4. doi: 10.1186/1755-8794-8-S4-S4 (PMC4682379; doi:10.1186/1755-8794-8-S4-S4)
Supplement: Additional file 9 — new 8. Docking pose analysis of 28 core proteins. The docking poses analysis shows the key residues of the proteins which interact with the ligands. The analysis reveals more mechanisms to help us design de novo drugs. [file 1755-8794-8-S4-S4-S9.pdf]

Additional File 9: Docking pose analysis of 28 core proteins

The docking poses analysis shows the key residues of the proteins which interact with the ligands. The analysis reveals more mechanisms to help us design *de novo* drugs.

01. BRCA1 (4IFI)

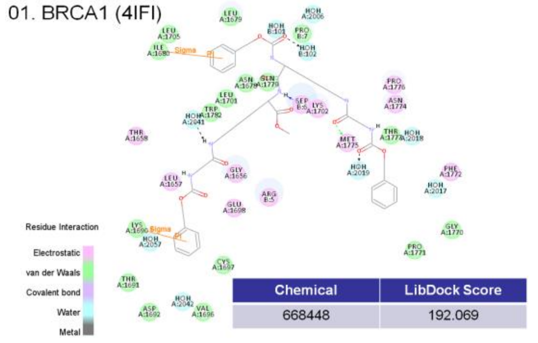

Chemical	LibDock Score
668448	192.069

02. CDK2 (3QQK)

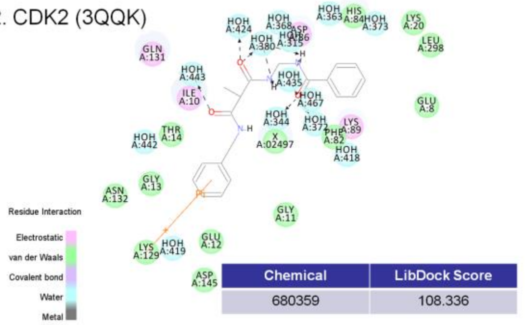

Chemical	LibDock Score
680359	108.336

03. CEBPB (2E42)

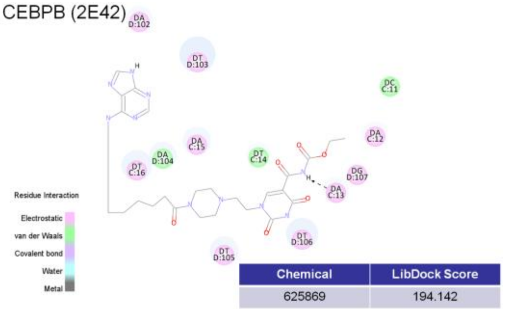

Chemical	LibDock Score
625869	194.142

04. CREBBP (4A9K)

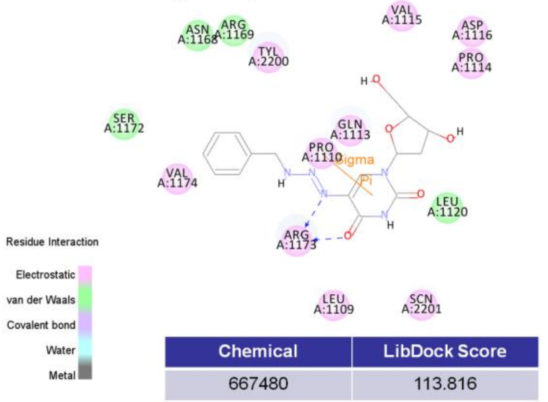

Chemical	LibDock Score
667480	113.816

05. CTNNB1 (3XT7)

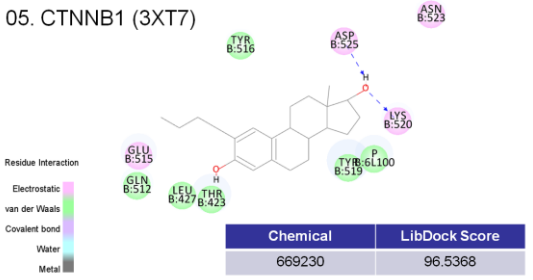

Chemical	LibDock Score
669230	96.5368

06. CUL1 (4F52)

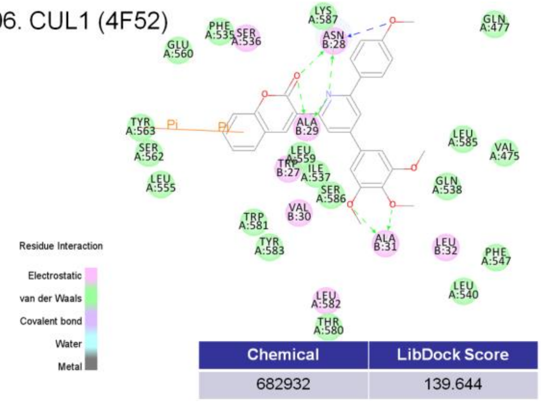

Chemical	LibDock Score
682932	139.644

07. CUL3 (4EOZ)

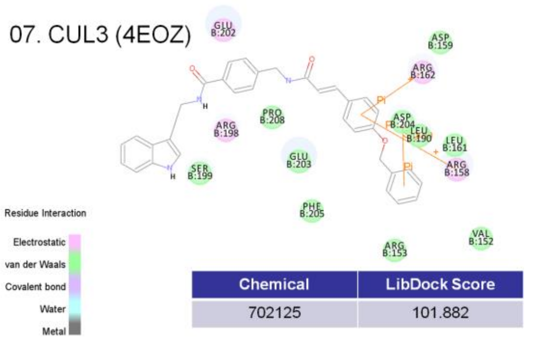

Chemical	LibDock Score
702125	101.882

08. EP300 (4BHW)

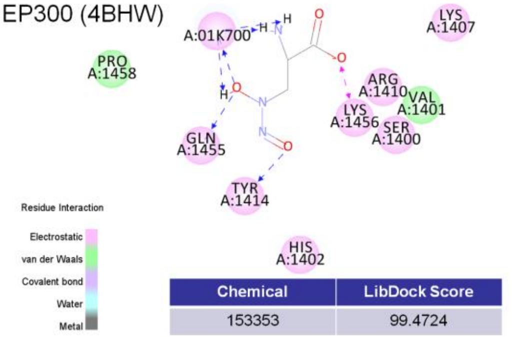

Chemical	LibDock Score
153353	99.4724

09. ESR1 (1UOM)

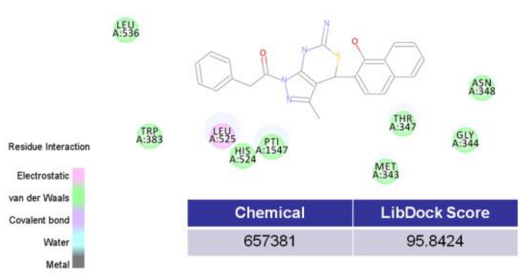

Chemical	LibDock Score
657381	95.8424

10.HDAC1 (4BKX)

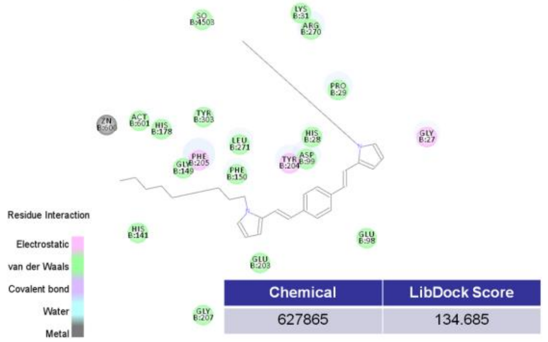

Chemical	LibDock Score
627865	134.685

11. HDAC2 (4LY1)

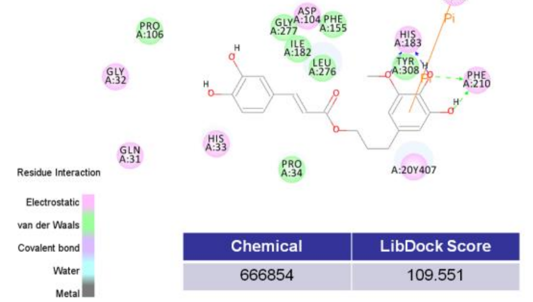

Chemical	LibDock Score
666854	109.551

12.HDAC4 (2VQM)

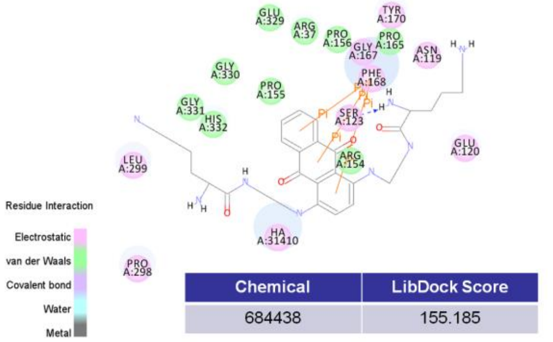

Chemical	LibDock Score
684438	155.185

13. IRAK4 (2NRU)

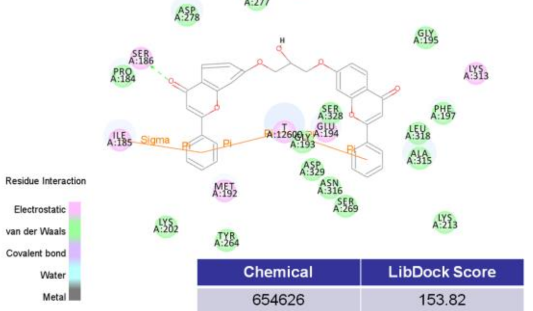

Chemical	LibDock Score
654626	153.82

14. ISG15 (3SDL)

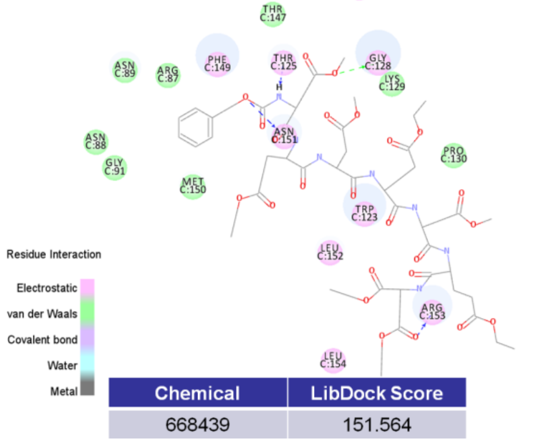

Chemical	LibDock Score
668439	151.564

15. KIAA0101

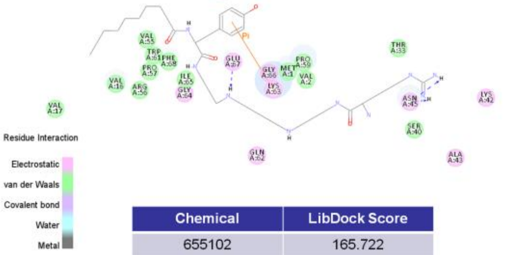

Chemical	LibDock Score
655102	165.722

16. MDM2 (4MDN)

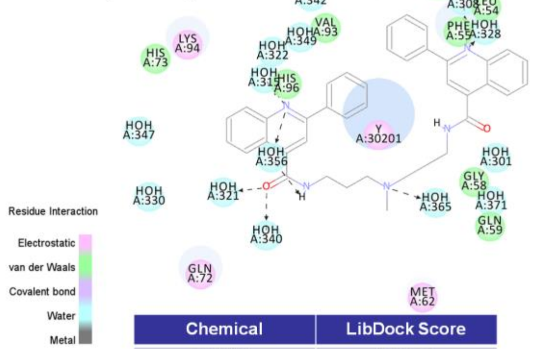

Chemical	LibDock Score
674086	136.098

17. MYC (1NKP)

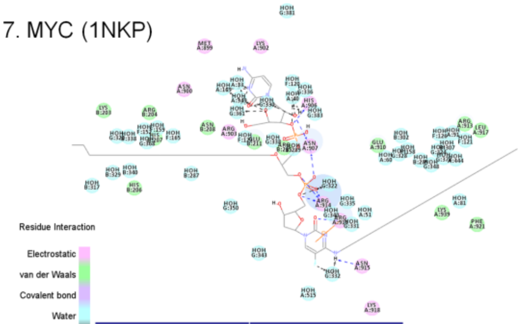

Chemical	LibDock Score
698686	194.107

18. PCNA (3WGW)

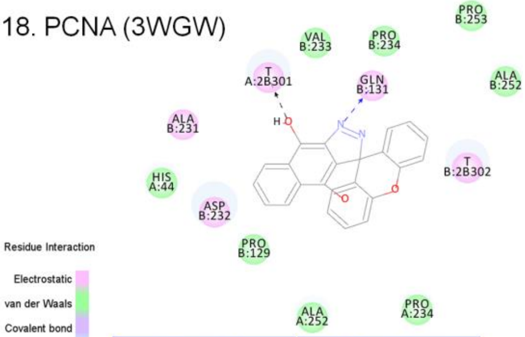

Chemical	LibDock Score
406433	122.435

19.PRKDC (3KGV)

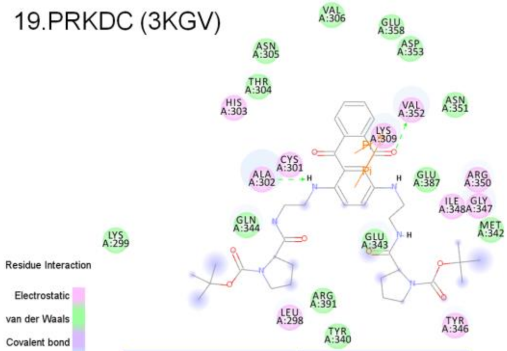

Chemical	LibDock Score
684443	144.774

20.PSMA3

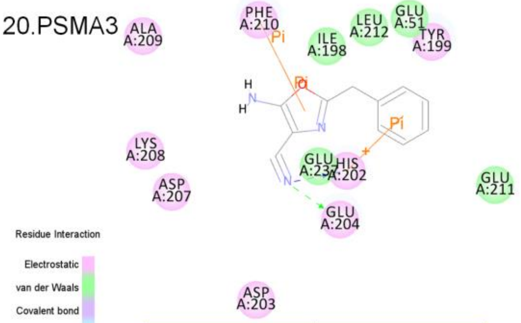

Chemical	LibDock Score
685116	100.962

21.RB1 (3POM)

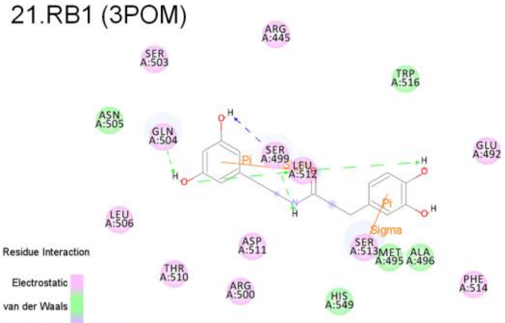

Chemical	LibDock Score
686584	130.737

22. (2SRC)

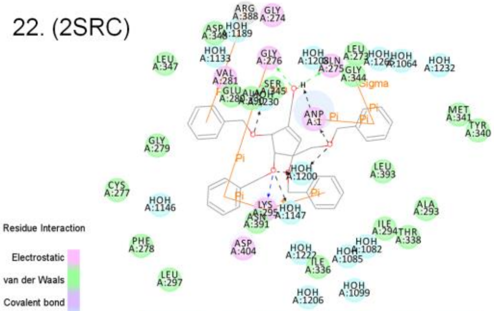

Chemical	LibDock Score
718308	153.089

23. TERF1 (3BQO)

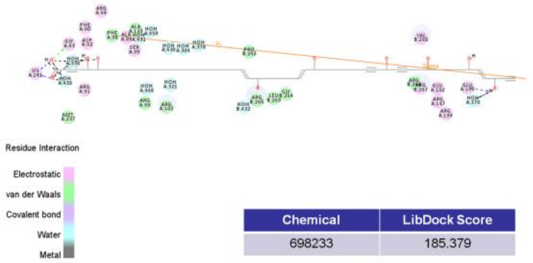

Chemical	LibDock Score
698233	185.379

24. TP53 (1TSR)

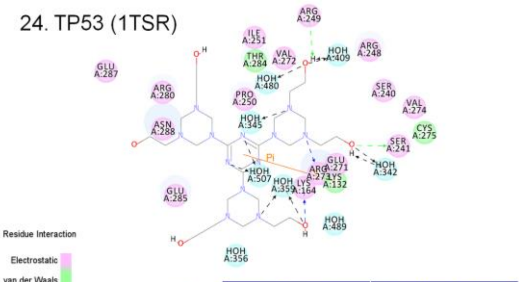

Chemical	LibDock Score
673172	126.112

25. TRAF2 (1D0A)

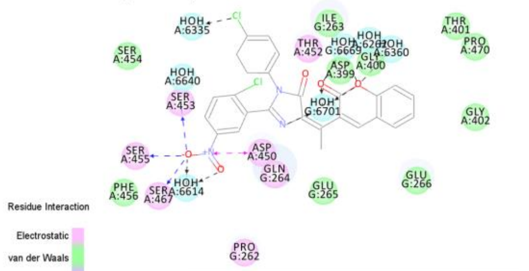

Chemical	LibDock Score
643737	95.6762

26. UBC (4FJV)

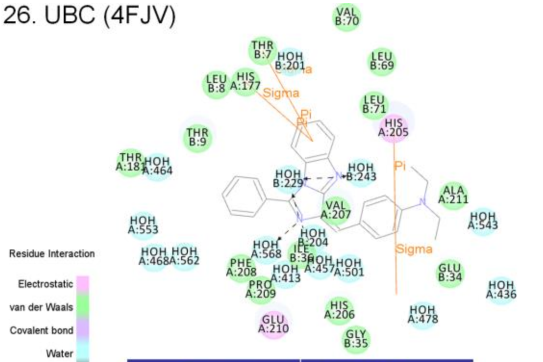

Chemical	LibDock Score
719481	122.887

27:XRCC6 (1JEQ) :Top 2 Ligand

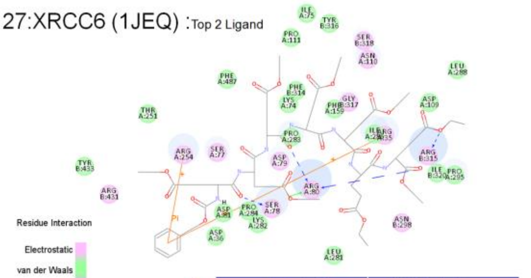

Chemical	LibDock Score
668449	241.902

28.YWHAZ (4HKC)

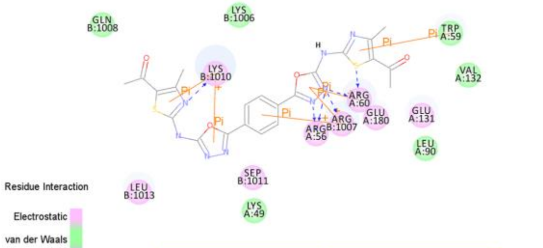

Chemical	LibDock Score
645900	116.939
