# Supplementary figures and images for: Multiple target drug cocktail design for attacking the core network markers of four cancers using ligand-based and structure-based virtual screening methods
Source: BMC Med Genomics. 2015 Dec 9;8(Suppl 4):S4. doi: 10.1186/1755-8794-8-S4-S4 (PMC4682379; doi:10.1186/1755-8794-8-S4-S4)

**Additional File 10. 54 ligands used for developing the Hypogen model**


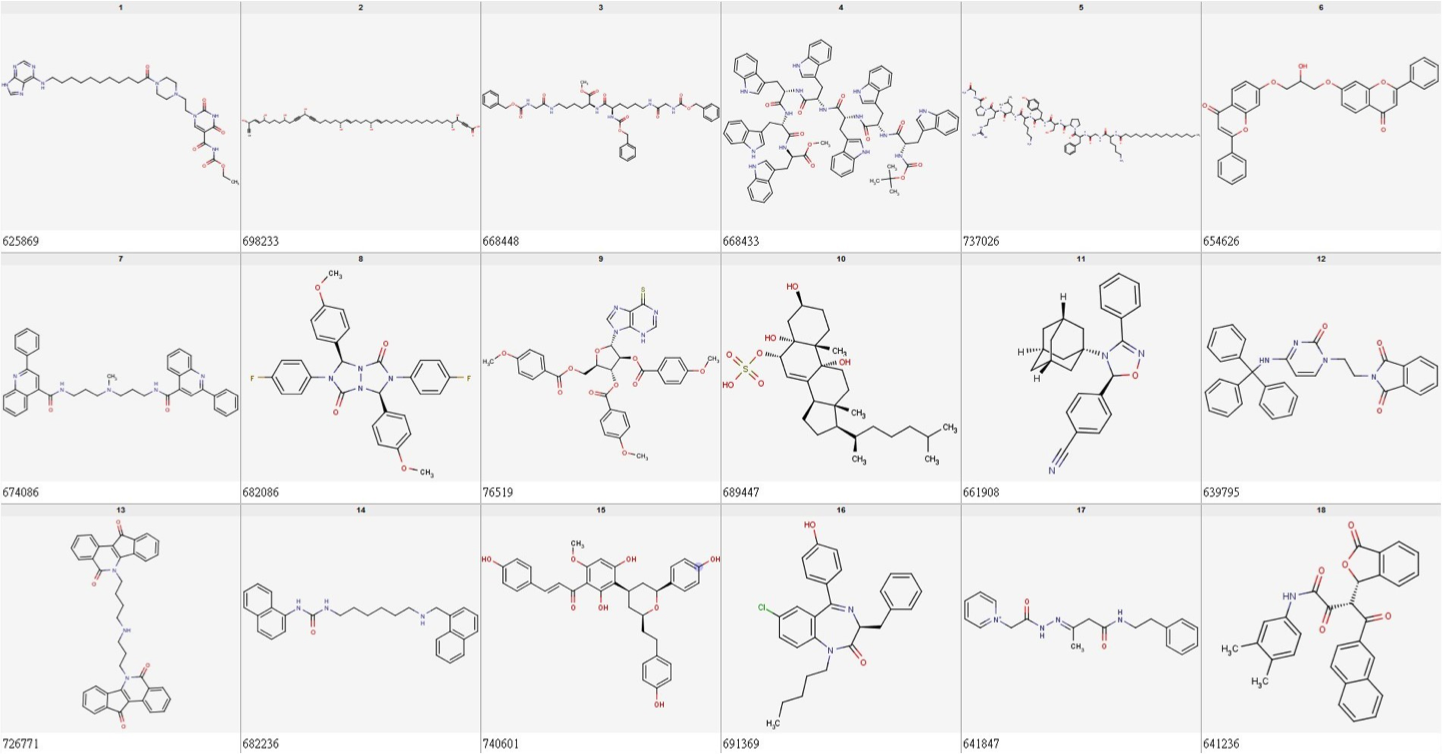


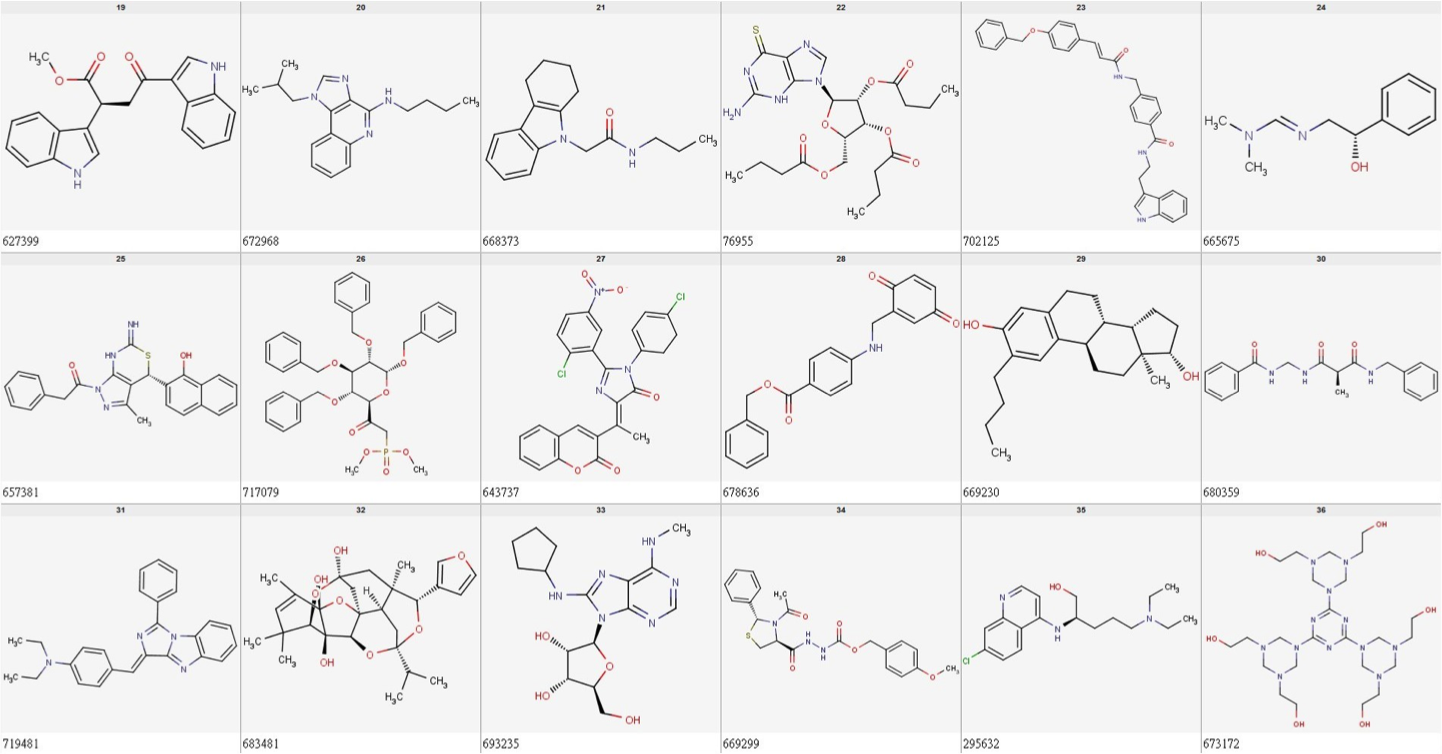


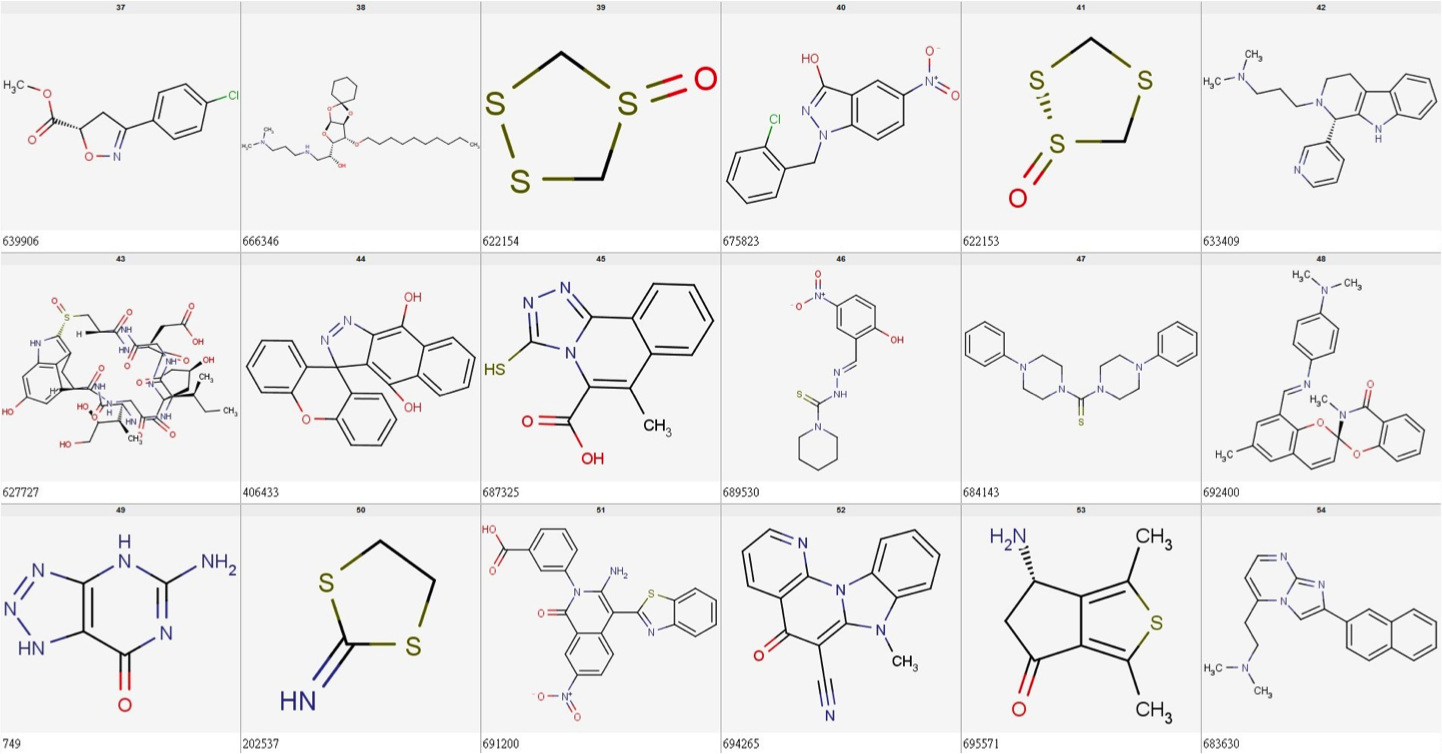

Supplement: Additional file 10 — new 9. 54 ligands used for developing the Hypogen model. [file 1755-8794-8-S4-S4-S10.docx]
